# Infectious Uveitis in Horses and New Insights in Its Leptospiral Biofilm-Related Pathogenesis

**DOI:** 10.3390/microorganisms10020387

**Published:** 2022-02-07

**Authors:** Bettina Wollanke, Hartmut Gerhards, Kerstin Ackermann

**Affiliations:** Equine Clinic, Ludwig-Maximilians-University, 80539 Munich, Germany; gerhards@lmu.de (H.G.); kerstin.ackermann@pferd.vetmed.uni-muenchen.de (K.A.)

**Keywords:** equine recurrent uveitis (ERU), *Leptospira* spp., biofilm infection, amyloid, neutrophil extracellular traps, Goldmann–Witmer coefficient, local antibody production, aqueous and vitreous samples, vitrectomy

## Abstract

Uveitis is a sight-threatening eye disease in equids known worldwide that leads to considerable pain and suffering. By far the most common type of uveitis in Germany and neighboring countries is classical equine recurrent uveitis (ERU), which is caused by chronic intraocular leptospiral infection and is the main cause of infectious uveitis in horses. Other infectious causes are extremely rare and are usually clinically distinguishable from ERU. ERU can be treated very effectively by vitreous cavity lavage (vitrectomy). For proper indications of this demanding surgery, it is necessary to differentiate ERU from other types of uveitis in which vitrectomy is not helpful. This can be conducted on the basis of anamnesis in combination with ophthalmologic findings and by aqueous humor examination. During vitrectomy, vitreous material is obtained. These vitreous samples have historically been used for numerous etiologic studies. In this way, a chronic intraocular leptospiral infection has been shown to be the cause of typical ERU and, among other findings, ERU has also been recognized as a biofilm infection, providing new insights into the pathogenesis of ERU and explaining some thus far unexplainable phenomena of ERU. ERU may not only have transmissible aspects to some types of uveitis in humans but may also serve as a model for a spontaneously occurring biofilm infection. Vitreous material obtained during therapeutically indicated vitrectomy can be used for further studies on in vivo biofilm formation, biofilm composition and possible therapeutic approaches.

## 1. Introduction

As in other species, uveitis in horses can be caused by various etiologies. Equine recurrent uveitis (ERU) is the main form of uveitis occurrence worldwide and is still the most common cause of blindness in equids [1,2,3,4,5,6,7,8]. The incidence of uveitis is 7–10% in Germany [9,10] and is reported to be 2–25% in the United States [5]. More than half of horses suffering from chronic uveitis will develop unilateral or bilateral blindness over time if only conservative therapy is performed [1,5,11], and many horses even have to be euthanized in the course of the disease [12]. For these reasons, uveitis in horses also has significant economic importance to the horse industry [2,4].

The term “ERU” is not used consistently in the literature. Many authors also refer to the chronic insidious uveitis of leopard coat pattern horses as “ERU” [5,7,13,14], whereas other publications distinguish between classic “ERU” and “leopard coat pattern uveitis”, not only because of the ophthalmological findings and the course of the disease, but also because the etiology is different for each type of uveitis [15,16]. In horses suffering from recurrent episodes of typical uveitis attacks in central Europe, an intraocular leptospiral infection is almost always detectable [16,17,18,19,20,21]. Herein, the term ERU will therefore be used exclusively for leptospiral-induced uveitis, which is the main representative of infectious uveitis in horses.

Often, previous reports, the patient’s signalement (e.g., age, breed, and coat color), the general examination (evidence of general disease, possible septicemia), and ophthalmologic examinations will indicate which form of uveitis is involved [22]. By far the most common types are equine recurrent uveitis (ERU) [1,5,7,11,12,15,16,23,24,25,26,27,28,29] and “leopard coat pattern uveitis” [5,12,15,30]. Less commonly, possible etiologies include traumatic uveitis [5,18], phacogenic uveitis [18,31], chronic iritis (similar to human Fuchs’ heterochromia iritis) [18,32], kerato-uveitis in severe corneal disease and co-reaction of the uvea [5], septicemia-accompanying uveitis [18,19,28,33,34,35,36,37,38,39,40], uveitis induced by intraocular tumors [5], and endophthalmitis caused by pyogenic bacteria [5]. Very rarely, therapy-resistant uveitis results from infection with *Halicephalobus* (syn. *Micronema*) *deletrix* [41,42]. Parallel testing of numerous equine uveitis patients for *Borrelia* spp., *Toxoplasma*, herpesviruses, and bornavirus revealed no evidence that these pathogens cause recurrent uveitis in horses [35,43,44,45]. Glaucoma may occur as a sequela of uveitis or may cause uveitic irritation only in the course of the disease, but it is usually an independent clinical picture and has very different underlying causes [5,18,19,46,47,48].

A genetic predisposition to uveitis has long been suspected [29,49,50,51] and has further been investigated for both Appaloosas (belonging to leopard coat pattern horses) [1,7,52,53,54] and warm-blooded horses [55,56]. Genetic factors predisposing to uveitis include immune system characteristics (e.g., the composition of major histocompatibility complexes (MHCs), immunocompetence with respect to defense against infectious agents, or favoring an autoaggressive response) as well as the anatomic configuration of the eye, particularly of the lens [3,5,18].

Due to the discovery of intraocular biofilm formation of leptospires in ERU eyes [57], the aim of this review was to differentiate infectious uveitis from noninfectious uveitis, to characterize leptospiral induced ERU, and to highlight the possible pathogenesis of ERU with respect to intravitreal in vivo leptospiral biofilm production. Realization that ERU is a biofilm infection explains many characteristics of ERU that have been mysteries until recently: the recurrent episodes of uveitis, the unsatisfactory efficacy of antibiotics, the failure of the immune response to eliminate the pathogen, the accompanying autoimmune phenomena, and the difficulties in detecting the causative agent.

## 2. Equine Leptospirosis

Serologic surveys indicate that infection with leptospires is common in horses, but systemic leptospirosis is almost always subclinical and therefore is unnoticed [58,59]. The main carriers are small rodents, which excrete pathogenic *Leptospira* spp. in their urine as renal carriers and thus contaminate the environment, including horses’ drinking water and food [59,60]. In particular, water from standing sources and wet or swampy pastures present a risk for equine infections [60,61,62]. Oral mucous membranes, conjunctiva, nasal mucous membranes, and skin lesions are considered sites of entry for the pathogens [59,63].

Numerous leptospiral serovars from different serogroups can lead to infections in horses [58,59,64,65]. The dominant serovar in Germany and many neighboring countries is Grippotyphosa, followed by the serovar Bratislava [19,66,67,68,69]. In countries further east and in the United States, infections with serovars from the Pomona serogroup are described in particular [19,58,63]. The geographical region in which the horses are located and which carriers shedding leptospires are present is decisive for which serovar leads to infection [13].

In rare cases where horses develop clinically apparent leptospirosis during systemic infection, the symptoms are similar to those of other species, such as humans or dogs (among others: disturbed general condition, fever, anemia, jaundice, hemoglobinuria, and impaired renal function). Clinically apparent courses mainly, but not exclusively, affect foals and young horses [40,59,63,70,71,72,73].

Leptospirosis should also be considered in abortions [70,74,75,76,77,78]. After leptospiral-induced abortion, leptospires are detectable in placenta and fetal tissue [75,79,80,81,82]. In addition, leptospires appear to be detectable in the uterus for at least several months after these abortions, although serum tests for anti-*Leptospira* antibodies are of no value in this context [83].

After leptospires are eliminated from well-vascularized structures, they can survive in immune-privileged sites (e.g., proximal renal tubules and vitreous cavity) for extended periods of time [63], and in the eye for many years [19,57]. Studies have shown that leptospires may also be detectable in the kidneys of healthy horses that have been slaughtered [84,85,86] and that between 8% and 35% of horses without an impaired general condition (e.g., mares after abortion, horses after flooding events, and horses with ERU) can excrete leptospires in the urine [64,87,88]. The importance of horses as shedders (e.g., via urine or placenta) for the infection of other horses is difficult to assess thus far.

The numerically and economically most important sequela of leptospirosis in horses is ERU, which, similarly to leptospiral uveitis in humans [89,90,91,92,93,94,95], is a late sequela of systemic infection, and typically occurs many months to years after acute infection [13,18,19,58,96,97,98,99,100,101,102,103].

## 3. Historical and Preliminary Remarks on the Etiology of ERU

Symptoms of uveitis in equids have been known and feared for centuries. Especially at times when people were still dependent on the labor of horses for working the fields, locomotion, and conflict, their welfare was of paramount importance. Diseases and the blindness of horses played a significant economic role. In this context, symptoms of uveitis in horses are said to have been described by Pliny the Elder (23 to 79 AD) in his *Historia Naturalis*. Later, a description of uveitis symptoms was found, among others, in the *Mulomedicina Chironis* (an equine medical treatise from the 4th century AD, book II, chap. 77, VII) (citation in [104]). The well-known Scottish human surgeon James Wardrop successfully competed for a prize offered by the Board of Agriculture in 1819 for the best “Essay on the Diseases of the Eye of the Horse, and on Their Treatment” [105,106]. Since the end of the 19th and the beginning of the 20th century, very characteristic descriptions of recurrent uveitis bouts in horses have been published repeatedly, and different infectious causes for this equine ocular disease have been postulated by various authors (numerous citations in [107,108,109,110]).

As early as the end of the 19th century, the occurrence of recurrent uveitis in horses was associated with keeping horses in humid regions, summers with high precipitation, clayey and marshy soil, and floods [111,112,113], which was confirmed in later publications [50,51,58,103,114,115,116,117].

In the beginning of the 20th century, Bayer (Vienna, Austria), in his detailed chapter on moon blindness or periodic eye inflammation of his book *Augenheilkunde* (*Ophthalmology*), among other things, details the causes of the disease and discusses its possible heredity. At that time, this had been resolutely asserted by some authors, and just as resolutely denied by others. Bayer summarizes the knowledge of the international literature at that time in such a way that the more frequent occurrence of the disease with descendants of ill parent animals is to be explained with bad soil conditions of the respective studs, because the descendants inevitably stayed on the same terrain as the parent animals. Where the overall incidence of the disease is low, offspring are also less likely to contract the disease. Bayer also reported on a breeding experiment with a stallion that was moonblind on one side and a mare that was moonblind on both sides. The foals were born with healthy eyes and did not become blind. Humid climates and marshy, boggy pasture soils or low-lying horse pastures with flooded meadows, as well as “wet years”, favored the development of ERU, whereas keeping horses in dry areas with limestone soils served to prevent the disease [51].

In 1906, Bayer considered different infectious agents, mostly bacteriae, incorporated by contaminated food and water, to be the main cause of ERU [51]. Notably, he had stated that ERU had to be considered an infectious disease. It should be termed “a non-purulent panophthalmitis, induced by microorganisms”, although the infectious agent had not yet been identified at that time. Bayer thought that some microorganisms could reside in an encapsulated condition in the body or in the eye until being reactivated under favorable circumstances. Furthermore, in an epidemic of “jaundice” described by Bayer, which had been observed in Lorraine in 1874, symptoms consistent with acute leptospirosis and uveitis in horses were observed.

However, a link between ERU and leptospiral infection was first described in 1946 by Gsell (Switzerland), when very high anti-*Leptospira* antibody titers were found in the aqueous humor of a horse suffering from ERU [89]. Repeated detection of anti-*Leptospira* antibodies in aqueous humor and vitreous fluids from ERU eyes supported an intraocular leptospiral infection [118,119,120,121,122,123], and there were a few reports of the pathogen being cultured from aqueous humor samples [25,124].

After both spontaneous [70,96,97,98,125] and experimental [97,100,101,126] infections with leptospires, the occurrence of ERU has been described in up to 100% of horses over the following months and years. However, many attempts to detect the pathogens in intraocular specimens from affected horses have failed [96,101,118,122,126,127,128,129,130], and ERU can occur months to years after the systemic infection. It was finally assumed that leptospiral infections somehow trigger ERU, but the pathogens themselves are no longer present when episodes of uveitis occur, and that ERU is a postinfectious uveitis, which is triggered by various mechanisms and immune reactions [96,101,128,131,132,133,134]. This autoimmune pathogenesis is still held by many authors, possibly also because, especially in the United States, no differentiation is made between “ERU” and uveitis in horses with leopard coat patterns. In the United States, many Appaloosas, which belong to leopard coat pattern horses, and which predominantly do not suffer from infectious uveitis, are included in the investigations (Section 4.2).

A connection between leptospiral infections and ERU is now generally accepted by most equine ophthalmologists; however, for a long time, it remained unclear how a systemic leptospiral infection could lead to uveitis attacks years later [1,3,5,13,14,63,135,136,137]. The apparent absence of the pathogens from the eyes is irritating and seems to lead to a commingling of various immune responses [6,101,133,138,139,140,141,142,143,144] with an “autoimmune disease” [5,145].

In particular, a research group around Parma has intensively studied cross-reactions, immunologic mimicry, and specifically, the occurrence of similar epitopes of leptospires and equine corneas [146,147,148,149,150,151]. Cross-reactions between leptospires and the uvea [126,152], cross-reactions between leptospires and lens proteins [153], and epitope spreading have also been reported [154]. In a subsequent study, at least for *L. grippotyphosa*, no cross-reactions with proteins of the cornea, lens, vitreous, and retina could be detected [45].

Altogether, many of the immunological processes described for human autoimmune diseases [155,156] have also been found in horses suffering from ERU [133,134,142,157,158,159,160,161,162,163,164,165,166,167,168,169,170,171,172]. Only a few authors considered that, in addition to autoimmune etiology, the permanent presence of leptospires in an “atypical” form could also be considered as a cause of recurrent episodes of uveitis [63,173,174]. In addition to the primary trigger for uveitis, environmental conditions, and especially the individual genetic make-up, are considered important in the development of ERU [175].

### 3.1. Leptospirosis and Autoimmune Disease

An autoimmune disease usually implies that no causative therapy is possible and that symptoms can only be alleviated by medical therapy. It is also known that various infectious agents can contribute to the development of autoimmune diseases, but that the autoimmune-related inflammation eventually persists independently of the infection as postinfectious autoimmunity [155,176,177,178,179,180,181,182].

In contrast, in ERU, further episodes of uveitis can be prevented very convincingly by the removal of vitreous material and flushing the vitreous cavity (vitrectomy) [16]. However, because vitrectomy only removes vitreous material and leaves the intraocular retinal antigens and lens untouched, the unusually high percentage of postoperative recurrence-free cases can be explained only by removal of intraocular leptospiral infection in the vitreous cavity [19]. An “autoimmune disease” would not explain the good prognosis of vitrectomy [183]. Although irrigation of the vitreous cavity temporarily relieves autoimmune uveitis by removing the “immunologic memory” from the vitreous cavity [184,185,186,187,188,189,190]; in these cases, it does not have the lasting and significant effect that it has in ERU [16,191]. Thus, the autoimmune reactions in equine eyes are only immune phenomena accompanying the causative leptospiral infection. Once the infection is eliminated, autoimmune reactions do not cause any further problems.

Autoaggressive immune responses are detectable (e.g., autoantibodies and autoantigen-specific T cells); therefore, ERU is nevertheless referred to as an “autoimmune disease” in many papers. Thereby, it seems to be irrelevant what causes the autoimmune reactions [163,169,192]. However, studies of the working group of Deeg [157,158,159,165,170,193] were performed with samples from horses in which a chronic intraocular leptospiral infection was present [17,18,19,20,194,195,196,197,198,199] and in which no further inflammation occurred after vitrectomy [16], i.e., no further autoimmune disease occurred either. For this reason, it would be preferable to refer to “autoimmune reactions accompanying infections” rather than “autoimmune disease” [19,45].

One argument by supporters of the theory that ERU is an autoimmune disease is that uveitis improves with treatment with corticosteroids and nonsteroidal anti-inflammatory drugs [145,158,200]. However, this disregards the fact that, in principle, treatment with corticosteroids and nonsteroidal anti-inflammatory drugs is indicated for almost every case of uveitis, regardless of its etiology [189,201,202,203,204,205,206]. In human leptospiral uveitis, treatment with corticosteroids is also indicated [92,207]. Thus, improvements in acute inflammatory episodes under topical treatment with corticosteroids do not contradict the presence of a chronic infection.

Immune responses which are independent of the etiology of uveitis always include autoimmune responses, which are detectable in any disruption of the blood–ocular barrier. The presence of pathogenic *Leptospira* spp. is a good explanation for the autoimmune reactions detected in ERU eyes, which cease after elimination of the infection [16,19,208]. Autoimmune reactions are a well-known phenomenon in chronic infections, and there are several mechanisms that can lead to autoimmune reactions in chronic infections [178,209,210,211,212,213,214,215].

## 4. Types of Equine Uveitis and Differential Diagnosis

In many cases of uveitis, the etiology, and thus, also the therapy, can be determined based on the clinical and ophthalmologic examinations. However, sometimes it is only during the course of the disease that it becomes clear which type of uveitis is present—or additional laboratory tests, possibly also the examination of aqueous humor, are indicated in order to decide on the further course of action. In rare cases, an etiological classification of the uveitis is not possible.

### 4.1. Equine Recurrent Uveitis (ERU)

ERU is by far the most common form of uveitis in horses in central Europe. ERU is a sero-fibrinous, although rarely sero-hemorrhagic inflammation of the uvea, which occurs acutely, is chronically recurrent, and can lead to blindness due to progressive destruction of the intraocular structures [28]. In about one-third of equine patients, both eyes are affected, often with a time lag of a few weeks to about 2 years [18,51,216].

The disease has also been termed periodic ophthalmia, although it had long been clear that, generally, the symptoms did not reoccur periodically. Often, very painful episodes of uveitis occur at varying intervals, and the inflammation-free intervals between episodes can be less than 2 weeks or up to more than 1 year. In most cases, recurrences occur within several weeks to several months, the intervals between relapses usually become shorter over time, and subsequent episodes of inflammation often increase in severity [19,217,218]. The typical symptoms of ERU have been described repeatedly and in broad agreement [5,11,18,23,24,25,26,27,28,107,219].

(a) Acute uveitis

The first two points are non-specific, but prompt horse owners to request a veterinary examination:

- Epiphora, blepharospasm, and photophobia;
- Concomitant conjunctivitis;
- Gossamer to smoky corneal opacity (Supplementary Material S1, Figures S1 and S2);
- After a short time, circular vascular ingrowth into the cornea may begin (Supplementary Material S1, Figures S1–S4);
- Inflammatory products in the anterior chamber of the eye (positive Tyndall effect, fibrin, leukocytes and/or erythrocytes) (Supplementary Material S1, Figures S3–S5);
- More rarely, also a hypopyon (Supplementary Material S1, Figure S3);
- Pain-related and often complete miosis; mydriasis can often only be achieved with a striking delay, and possibly incompletely with medication (Supplementary Material S1, Figures S1–S4);
- Diffuse vitreous haze (Supplementary Material S1, Figures S6 and S7);
- In rare cases, rubeosis iridis (Supplementary Material S1, Figure S8).

After mild inflammation and immediate rigorous conservative treatment of acute uveitis, the eye may still look unchanged ophthalmoscopically in the inflammation-free interval after the first episodes, or only very subtle changes may be left that are only noticed by veterinarians experienced in equine ophthalmology and with a maximally dilated pupil. The more uveitis episodes which have passed, the more changes can be seen in the course of time. even in the inflammation-free interval; thus, the diagnosis “ERU” can even be reliably made between acute inflammatory episodes. The findings therefore include:

(b) Chronic recurrent uveitis, inflammation-free interval

- Atrophy of the globe (often resulting in the development of a so-called “3rd corner of the eye” occurring on the upper eyelid, Supplementary Material S1, Figure S9);
- Chronic keratitis;
- Posterior (more rarely, also anterior) synechiae, sometimes only iris residues on the anterior lens capsule (Supplementary Material S1, Figures S10 and S11);
- Vesicular cataract, typically in the periphery of the posterior surface of the lens (Supplementary Material S1, Figure S12);
- Cataract formation (cataract typically originating from the lens capsule: either as a result of posterior synechiae or as a result of inflammatory products adhering to the posterior surface of the lens) (Supplementary Material S1, Figures S8–S11);
- Vitreous liquefaction;
- Sometimes chronic diffuse vitreous haziness, which does not completely resolve in the inflammation-free interval (Supplementary Material S1, Figures S10 and S13);
- Over time, increasing accumulation of dense cloudy to membranous inflammatory deposits in the vitreous body (“floaters”), which are not resorbed in the inflammation-free interval, typically starting high dorsal in the liquefied vitreous, floating with eye-movements (Supplementary Material S1, Figure S13);
- Centrifugal retinal folds surrounding the optic nerve disc, bullous, planar, or complete retinal detachments (Supplementary Material S1, Figures S14 and S15);
- More rarely also lens (sub-)luxations,
- In later stages, significant bulbar atrophy, corneal opacities due to collapsed anterior chamber and extensive contact between iris and corneal endothelium, phthisis bulbi (Supplementary Material S1, Figures S9 and S11);
- Very rarely: glaucoma (e.g., in later ERU stages after cataract formation and subsequent subluxations or luxations of the lens).

In the case of an initial uveitis flare-up, blunt trauma is sometimes considered as a differential diagnosis. After the acute uveitis has subsided, a careful ophthalmoscopic examination should be performed in mydriasis to detect inflammatory deposits dorsally in the vitreous. If vitreous deposits are present, there is strong evidence that the acute inflammation was a first episode of ERU. If the eye is inconspicuous again after the episode has subsided, the diagnosis of “ERU” may not be made unless another uveitis episode occurs. In horses in Germany and neighboring countries, the symptoms described here are highly specific for chronic intraocular leptospiral infection leading to the uveitis episodes [16,17,18,19]. In human *Leptospira*-induced uveitis, which, as in horses, is a late consequence of systemic infection, ophthalmologic examination (and, among other findings, the presence of vitreous opacities) is also considered very sensitive and specific to establish an etiologic diagnosis [220].

Most eyes exhibit classical symptoms of ERU, with recurrent painful attacks. However, in about 3% of eyes, chronic inflammation of the posterior segment (vitreous cavity, ciliary body, and choroid) dominates the disease [15]. In these cases, uveitis causes less pain in the beginning of the disease (until the anterior segment is also affected—which happens in most eyes after a while), and eventually lead to blindness (retinal detachment and/or cataract formation) before the owner notices any problem. These cases of intermediate and/or posterior uveitis are also highly specific for leptospiral uveitis in horses without a leopard coat pattern [17,18,19,217].

If repeated inflammations have occurred without leaving ophthalmoscopically recognizable sequelae, it is advisable to re-examine the horse in an acute episode, e.g., to differentiate between recurrent keratitis and uveitis. Alternatively, an aqueous humor examination could be performed [132] (Section 7). If leopard coat pattern horses are presented with symptoms consistent with ERU, aqueous humor analysis is also indicated. The results can then be used to decide on further therapy [20,221]. In cases of positive leptospiral findings, the prevention of recurrence can be achieved by vitrectomy [16,19,222].

ERU is by far the most common form of equine infectious uveitis and, with early and correct treatment, now has a good prognosis for both permanent absence of recurrences and preservation of vision [16,191].

### 4.2. “Leopard Coat Pattern”—Uveitis

The term “insidious ERU” is widely used for uveitis in leopard coat pattern horses, but it is misleading and a contradiction in terms. In leopard coat pattern horses, because there are no typical recurrent episodes of uveitis, but uveitis is distinguished by a persistent low-grade intraocular inflammation with a gradual and cumulative destructive effect, rather than outwardly painful episodes of uveitis [4,5,7,12,13,15,52,54,175], it would be more appropriate to differentiate between “ERU” and “insidious uveitis” rather than use the term “Insidious ERU”, which is an oxymoron. In addition to the fact that horses with leopard coat patterns typically do not exhibit classic painful recurrent episodes of uveitis, most often, there is no infectious etiology of their uveitis either [1,15,16,197,223]. For this reason, and to clearly differentiate this form of uveitis from ERU, the term “leopard coat pattern uveitis” was introduced [15].

However, in many studies in the United States, Appaloosas, as the main representative of leopard coat pattern horses, comprise a large proportion of ERU patients [4,5,12]. There are no painful uveitis attacks; therefore, the veterinarian is often only consulted when the owners have noticed opacities of the cornea or lens, bulbar atrophy, or buphthalmos (due to glaucoma), or when an impairment of vision is suspected. At this point, irreversible changes are often present, and the further course of the disease can hardly be influenced [5,12].

Appaloosas not only have an eightfold increased risk of developing uveitis compared with other horses [1,15,30], but the course of uveitis is also particularly unfavorable and both eyes are usually affected, often meaning that both eyes lose vision in the course of the disease [5,7,12,15,30].

A lens pathology is often the primary condition in leopard coat pattern horses (Supplementary Material S1, Figure S16). Relatively often and early in the course of the disease, a (sub-) luxation of the lens and/or a cataract occur [1,15,30,153,224,225,226,227]. Lens changes are often present at very early stages of disease (even without concomitant synechiae). The lens changes occur without an antecedent painful iritis and without inflammatory products on the posterior surface of the lens; therefore, the etiology in these patients could be autoimmune-related inflammation, e.g., due to protracted leakage of lens proteins through an abnormally configured lens capsule (Section 4.4). Amyloids can be formed by refolding of the lens proteins in the inflamed milieu [228], and amyloids are detectable in leopard coat pattern horses in the chamber angle and trabecular meshwork, especially in eyes with a lens pathology [224].

A relatively large number of horses with leopard coat patterns (Knabstruppers more often than Appaloosas) develop glaucoma [30], some develop phthisis bulbi (Appaloosas more often than Knabstruppers), and somewhat less frequently, vitreous opacities are also present [5,7,12,13,30].

Sometimes, ophthalmoscopic findings are present in leopard coat pattern horses (e.g., hypotony of the globe, synechiae, cataract, vitreous opacities) that cannot be clearly differentiated from ERU. Since leopard coat pattern horses may also have intraocular leptospiral infection in some cases, aqueous humor analysis may be indicated [15,16,30,198,199,223]. Only if the aqueous humor analysis shows evidence of a leptospiral etiology the infection can be eliminated by vitrectomy, and by this, vision may be preserved permanently—provided there is no additional leopard coat pattern uveitis [15].

### 4.3. Traumatic Uveitis

Both blunt trauma and injury to the sclera or cornea may be followed by concomitant uveitis due to transient damage to the blood–ocular barrier and disturbance of the immunologic balance in the eye [229]. The following inflammation is immediately downregulated by the effective immunosuppressive mechanisms in the eye, and the basic immunosuppressive setting of the eye is restored as far as possible [139,230,231]. If corneal or scleral injury is evident when uveitis occurs, the traumatic etiology of this uveitis is obvious. After blunt trauma, significant hyphemia in the anterior chamber of the eye or lesions around the eye or on the eyelid skin may be evidence of trauma. However, sometimes it is difficult to decide whether the first noticed ocular inflammation is a result of trauma or a severe sero-hemorrhagic uveitis in terms of ERU [5,28,232] (Supplementary Material S1, Figure S17).

This makes no difference for the initial treatment of acute uveitis. If the uveitis improves satisfactorily with conservative treatment, and if there is no ophthalmoscopic evidence of a previous uveitis event after the inflammation has subsided, mydriasis has been achieved, and the hemorrhagic effusion has resorbed, it is possible to simply wait and observe the eye for another inflammation. If uveitis recurs, then trauma is unlikely to have been the cause of inflammation and ERU can be assumed as the most likely diagnosis [28].

If the blood does not reabsorb satisfactorily from the anterior chamber, and therefore either a fibrinolytic is injected or the blood has to be removed mechanically from the anterior chamber, an aqueous humor sample can be taken during the procedure and tested for anti-*Leptospira* antibodies and the *LipL32* gene [28].

Rarely, foreign bodies remain in the eye after perforating injuries, which then either leads to therapy-resistant uveitis or the uveitis immediately recurs after the discontinuation of anti-inflammatory treatment. If the foreign body can be removed, this also resolves the uveitis. In other cases (e.g., the foreign body cannot be removed or endophthalmitis has developed after perforating bulbar trauma), enucleation is indicated [28].

### 4.4. Phacogenic Uveitis

In phacogenic uveitis, there is a leakage of lens proteins through the lens capsule, resulting in an autoimmune reaction [226,233]. This can be the case both with a congenital weakness of the lens capsule and as a result of trauma with damage to the lens capsule. Often, the ophthalmologic findings are indicative of whether the condition is phacogenic uveitis or not. Especially in phacogenic uveitis after trauma, the leakage of lens protein is recognizable. In cases of congenital weakness of the lens capsule, the configuration of the cataract also often indicates that the uveitis is not ERU. Congenital or juvenile phacogenic uveitis is often milder than typical ERU attacks and may be self-limiting. If bleb-like structures are visible in the center of the anterior surface of the lens, hernia-like lens parenchyma protrudes here and iris residues may also adhere; this cannot reliably be differentiated from ERU, because such changes may not only be the cause, but also the consequence, of uveitis [5] (Supplementary Material S1, Figure S18). In these cases, when in doubt, an aqueous humor analysis with regard to a leptospiral infection should be considered [18,28].

### 4.5. Chronic Iritis, Similar to “Fuchs’ Heterochromic Iritis” in Humans

Chronic equine iritis, which resembles Fuchs’ heterochromic iritis described in human medicine [234,235,236], is usually insidious–progressive and is not accompanied by painful inflammatory episodes. Horses are typically not referred until corneal edema has developed, which is recognized by the owners. If the interior of the eye is still visible, irregular minor depigmentation of the iris, and sometimes also posterior synechiae, may be recognizable (Supplementary Material S1, Figure S19). A comparison with the second eye can reveal a diffuse loss of the normally saturated iris coloration. In the posterior segment of the eye (posterior surface of the lens, vitreous cavity and fundus), typically, no changes are present. Chronic iritis causes precipitates to sediment on the corneal endothelium, sometimes leading to corneal edema without an increase in intraocular pressure. However, over time, the iridocorneal angle and the trabecular meshwork may also become obstructed with inflammatory products, resulting in an increase in intraocular pressure [18,32]. The clinical course and the ophthalmologic findings enable differentiation from ERU in most cases; thus, only rarely is aqueous humor analysis indicated. Compared with uveitis in leopard coat pattern horses, the course of chronic iritis is milder and less devastating in most cases.

### 4.6. Uveitis during Septicemia

In severe general infections, sero-fibrinous uveitis may occur [51], which then typically affects both eyes [237] (Supplementary Material S1, Figure S20). Most commonly, this septicemia-accompanying uveitis occurs in foals with rhodococcosis [36,38,219,238]. However, it may also occur in neonates in which other bacteria are causative for septicemia [37,39,239,240]. Whether septicemia-associated uveitis is an immune-mediated uveitis comparable to septicemia-associated non-septic and presumably immune-mediated synovitis, or whether the pathogens reside in the eye and are eliminated from the eyes as septicemia recedes, remains unclear without aqueous humor analysis [241,242]. In any case, to the best of the authors’ knowledge, foals that survive septicemia are not expected to experience recurrent episodes of uveitis. Septicemia-associated uveitis, in which pathogens may also enter the eye, is thus not an ERU. Foals up to 6 months of age affected by uveitis typically have septicemic uveitis and no ERU. ERU only occurs in horses older than 6 months of age [18].

There are also case reports of uveitis in the context of general infections with spirochaetes in adult horses—especially in severe and fatal courses. These include Lyme disease [33,34], as well as leptospirosis [40,70].

### 4.7. Uveitis Accompanying Intraocular Tumors

Intraocular tumors can lead to uveitis by mechanical irritation and/or immune responses. In human ophthalmology, the term “pseudouveitis” was coined for tumors which clinically mimic an inflammatory disease [243]. Intraocular tumors include melanomas [244,245,246,247], medulloepitheliomas [248,249,250,251,252,253] (Supplementary Material S1, Figure S21), and rarely lymphomas [219,254,255,256]. Intraocular tumors can often be detected by ophthalmologic examinations based on non-physiologic tissue formations; in cases of media opacity, sometimes only with the help of ultrasound examination. The distinction from ERU is clear when tumor tissue is visible or can be visualized by ultrasound. More rarely, general diseases indicate a tumor and involvement of the eye in the form of more or less therapy-resistant uveitis. This often results in considerable media opacities, in which etiological assignments of the uveitis are not reliable. In cases of doubt, aqueous humor analysis is indicated to exclude infectious uveitis and to perform cytological diagnostics.

### 4.8. Uveitis during Parasitic Infections

Relatively rarely, *Halicephalobus* (syn. *Micronema*) *deletrix* infections can cause therapy-resistant uveitis [41,42,257]. If there is no satisfactory improvement on the known therapy of acute uveitis, it is advisable to consider a general disease and, if necessary, to initiate blood tests including electrophoresis. If micronematosis is suspected (clearly impaired general condition, poor nutritional status, very high total protein in the blood, ß-peak in electrophoresis), ultrasound examination of the kidneys and urinalysis would be indicated, among other tests, because the urinary apparatus is often involved in this infection. Thus far, there are only reports of fatal courses of this disease [258,259,260,261,262,263,264,265,266,267,268,269,270,271,272,273,274,275,276,277,278,279]. In early stages, and when the general disease is not yet in the focus, uveitis caused by these nematodes may not be reliably distinguished from ERU—even if the striking resistance to therapy of the uveitis is not consistent with ERU (Supplementary Material S1, Figure S22). Therefore, in cases of doubt, an aqueous humor analysis is indicated.

Additionally, infections with *Setaria* spp. are rarely described; they can lead to anterior uveitis and corneal opacities [280]. Usually, the parasites are then visible in the anterior chamber of the eye or can be visualized by ultrasound. Ophthalmologic symptoms are easily distinguishable from ERU.

### 4.9. Uveitis Accompanying Severe Keratitis

In cases of severe keratitis or infections reaching deep into the cornea, concomitant hypopyon uveitis may develop [5,219]. This may be a warning signal that endophthalmitis may be developing, and subsequently, a surgical intervention is required. Corneal disease dominates the ophthalmological findings, and, in most cases, this is easy to clinically distinguish from ERU (Supplementary Material S1, Figure S23).

### 4.10. Endophthalmitis

Endophthalmitis is usually the result of bulbar lesions or deep and infected corneal disease. More rarely, endophthalmitis is iatrogenically caused after intraocular surgery. Endophthalmitis caused by Staphylococcus spp. or other bacteria is accompanied by a highly disturbed general condition and fever. In early stages, irrigation of the anterior chamber, vitrectomy, and intraocular antibiotics can be used to try to eliminate the infection [281]. In advanced stages, enucleation is indicated. Visual function is lost; therefore, there is a risk of ascending encephalitis and phthisis would develop within a short period of time (Supplementary Material S1, Figure S24). Both the significant disturbance of the general condition and the ophthalmologic findings enable easy differentiation from ERU [5,28,219].

### 4.11. Autoimmune Uveitis and Uveitis of Unknown Etiology

In rare cases, also in horses that have no leopard coat pattern, the cause of uveitis cannot be determined. In these cases, aqueous humor testing is indicated to rule out an infectious and treatable cause [16]. If no intraocular infection and no causative disease for the uveitis is detectable, only symptomatic therapy is possible. After exclusion of the described types of uveitis and an infectious etiology (including other bacteria than *Leptospira* spp. and more exotic pathogens, such as viral, fungal, and protozoal infections involving the uvea), the suspicion of an “autoimmune disease” might be justified in single cases (Section 3.1).

## 5. Therapy

### 5.1. Acute Uveitis

Conservative therapy of acute uveitis—regardless of its etiology—always includes:

- Topical mydriatics, as often as needed for dilation of the pupil to prevent posterior synechiae and thereby cataract formation (in equids, atropine is by far the most effective substance for this purpose, and to the best of the authors’ knowledge, atropine can be used as frequently as needed without any concern (Supplementary Material S2));
- Topical treatment with corticosteroids to reduce inflammation (most effective are ophthalmic ointments containing dexamethasone)—if corneal defects are present, these are a contraindication for ophthalmic ointments containing corticosteroids, because otherwise the development of serious corneal ulcers may be favored;
- Systemic administration of non-steroidal anti-inflammatory drugs;
- Palliative measures: the avoidance of bright light, limited exercise, or even stall rest;
- Systemic administration of antibiotics can be considered in severe cases when a bacterial infection is involved (e.g., hypopyon–keratitis or ERU with severe haziness of intraocular fluids); thus, this is rarely indicated.

If the eye does not improve satisfactorily with this treatment, additional measures may be required. If diffuse vitreous opacification is persistent and significant, systemic administration of prednisolone may be considered. In eyes in which mydriasis is not achieved satisfactorily or in which the inflammatory products have not reabsorbed within 1–2 weeks, surgical procedures may be required (e.g., synechiolysis, the injection of fibrinolytics into the anterior chamber, or the mechanical removal of coagula from the anterior chamber of the eye) [5,28,216,219].

### 5.2. Chronic Course of Uveitis

Both leopard coat pattern uveitis, which is chronically destructive and not accompanied by painful episodes, and uveitis, which proceeds with recurrent painful episodes, lead to progressive damage to the intraocular structures; therefore, the goal is always to contain the inflammation as much as possible to prevent blindness or delay the process as long as possible.

In leptospiral-induced ERU, vitrectomy, which was established in horses more than 30 years ago [282,283], is the most effective therapy [67,68,284,285,286,287,288,289,290,291], even if not 100% of the vitreous humor can be removed (“subtotal vitrectomy”, because preventing retinal detachment and damage to the posterior lens capsule are crucial). After successful surgery, up to 97% of operated eyes do not develop recurrent uveitis episodes [2,16,191]. In this way, vision can be preserved in many horses—provided that the operation is performed before irreversible damage to the lens and/or retina has occurred [16,191,288]. Vitrectomy not only stops ERU and often prevents blindness [2,191], but by removing vitreous opacities, it can actually improve vision [16].

Vitrectomy is a highly specialized surgery that requires a high level of experience and skill on the part of the surgeon and a well-trained surgical team for optimal results. Any mishap by anyone involved and any malfunction of the equipment can be fatal to vision or eyeball preservation. In addition, specific equipment, such as custom-made instruments and reliable machines adapted to the dimensions of equine eyes are required, which represents a substantial investment and is only worthwhile if larger numbers of patients are to be expected. This could explain why vitrectomy in horses is still performed predominantly in specialized ophthalmological clinics in Germany and has not yet gained equal acceptance in other countries or continents [13,16].

In recent years, an injection of 4–6 mg gentamicin (which is really gentamicin sulfate, but is dosed according to the gentamicin content), preferably a preservative-free solution [292], into the vitreous cavity has been described as a less demanding therapeutic method for ERU [292,293,294,295]. This does not require specialized instruments and equipment, and performing the injection is easier and less expensive than vitrectomy [296]. These injections are not only reported to be effective for leptospiral uveitis, but are also discussed to reduce uveitis via immunomodulatory effects; therefore, surprisingly, gentamicin injections are not recommended exclusively for infectious uveitis [292,296]. However, there are only single studies on gentamicin injection in comparatively few patients, and there are still scant conclusive data on long-term outcomes. Disturbingly, the relatively high gentamicin dose administered exceeds concentrations largely considered “safe” to prevent retinal damage by almost threefold (Supplementary Material S3). None of these studies included ERG studies to monitor retinal function. In addition to intravitreal gentamicin injection, the intravitreal injection of a third-generation cephalosporin has also been mentioned but has not gained acceptance thus far [297].

Another treatment approach is systemic treatment with antibiotics that are effective against leptospires and achieve a sufficient therapeutic level in the intraocular fluids. For this purpose, the intravenous injection of enrofloxacin has been proposed [298]. In a later study, it was shown that although this treatment results in therapeutic levels in the eye (higher than the minimum inhibitory concentration (MIC) for leptospires), elimination of leptospires from the eye is not reliable. Without enrofloxacin administration, leptospires were culturally detected in 54% of vitreous samples from eyes affected with ERU; after several days of intravenous enrofloxacin administration and with drug levels in vitreous samples above the MIC, the culture was still positive in 30% of vitreous samples [196,299]. It can be concluded that systemic antimicrobial therapy, at least with enrofloxacin, is not very effective in eliminating the organism from the eye [63].

Unlike in horses, leptospiral uveitis in humans can be self-limiting [207]. Although recurrent episodes may occur and can be sight-threatening, leptospiral uveitis is reported to respond well to the systemic administration of antibiotics, and generally has a good prognosis [116,300,301]. Among other causes, this may be because the human vitreous body has a volume of only about 4 mL, whereas that of the horse is about 28 mL. The immunological niche is therefore much larger in horses. This might be an explanation why the elimination of the infection is more problematic. It is also possible that the equine vitreous is more susceptible to biofilm formation for other reasons.

In uveitis that does not have an infectious etiology, such as uveitis in leopard coat pattern horses, only symptomatic anti-inflammatory treatment, not causal therapy, is possible. In addition to the prolonged administration of NSAIDs and, if necessary, systemic corticosteroids, other therapeutic methods have been described to be effective locally. This includes the intrascleral or suprachoroidal implantation of cyclosporine devices, which deliver active substance over 1–2 years and counteract the intraocular inflammation [5,218,302]. Only a few publications distinguish between infectious and noninfectious uveitis and use cyclosporine devices exclusively for noninfectious uveitis [68].

Cyclosporine implants, unlike vitrectomy, are only effective for about 2 years and may then need to be replaced. In addition, the suppression of chronic uveitis with cyclosporine devices is less effective than vitrectomy for ERU [191,218,291,302,303]. It must be considered that the implants are not legally available in the European Union (any import into the EU from non-EU countries is prohibited by law). Moreover, the implants had predominantly been inserted incorrectly (episclerally instead of deep intrasclerally or subsclerally) in horses that had nevertheless somehow received implants [303]. This could explain the lack of effectiveness in several cases.

Other authors describe intravitreal [304] or suprachoroidal [8] injections of triamcinolone. However, thus far, there are only results from 36 eyes of 29 horses with a follow-up of merely 3 months. Long-term therapy with acetylsalicylic acid (25 mg/kg 1 × daily), which was recommended decades ago [11], has not yielded any long-term results, to the best of the authors’ knowledge, and this therapeutic method has not gained acceptance. Horses under treatment with anti-inflammatory drugs are not allowed to compete in equestrian sports (doping regulations), on animal welfare grounds.

In leopard coat pattern horses, progression of the disease can be effectively prevented by vitrectomy if only intraocular leptospiral infection is involved. In cases of significant inflammatory vitreous deposits, vitrectomy may be considered—even independently of a leptospiral infection in the eye—to improve vision and prevent the vitreous membranes from attaching to the lens. However, a further course of the disease cannot be stopped by this, only delayed. Otherwise, only symptomatic antiphlogistic and, if necessary, pressure-lowering therapy, is possible. Here, cyclosporine implants, for example, would be suggested to make the further course of the disease less severe and to delay blindness [218,302]. However, pharmaceutical regulations must be respected, because the import of cyclosporine devices into the European Union is prohibited or may only be carried out with special authorization [303].

## 6. Diagnostic Value of Testing Serum Samples for Anti-Leptospira Antibodies in Horses Suffering from ERU and in Horses with Healthy Eyes

Many studies have assessed serum samples from horses with ERU and horses with healthy eyes, and most often, the sera were analyzed by microscopic agglutination tests (MATs) [1,9,17,19,43,305,306,307]; ELISA tests for the detection of anti-*Leptospira* antibodies were used less frequently [194,195,198,308,309,310]. In some studies, no significant differences were detectable between groups of these horses; however, in other studies, differences were detected [1,9,43,135,305,306]. It is largely agreed, however, that serum assays using MAT and various ELISA tests are inappropriate for diagnoses of intraocular leptospiral infections in an individual animal [17,18,19,45,194,195,307,311,312,313,314,315,316]. Among other reasons, this can be explained by the fact that leptospiral infections are endemic in many regions and a high background level is present; thus, a large number of horses with healthy eyes display MAT titers [17,19,84,85,307,313,315,316,317,318,319,320,321,322,323,324,325,326]. Therefore, as in human leptospiral uveitis [327], MATs using sera are not diagnostic of leptospiral uveitis when only serum is examined [17,18,19,45,307,308,314,328].

MAT titers detectable in serum are mainly influenced by the type of horse stabling, its location, geographical region, climatic conditions, and age of the tested horses [59,320,322,323,325,326,329,330,331,332,333,334]. Laboratory diagnostics (MAT titers considered “positive” and serovars available) may have also influenced the results of these studies [308]. All of these factors are more decisive for the presence of agglutinating antibodies detectable in MAT than the presence of leptospiral uveitis.

It should also be noted that ERU is a late consequence of systemic leptospirosis. Episodes of uveitis often occur months and often even years after systemic infection; therefore, MAT titers in the blood may have already decreased or may no longer be detectable by the time the eye disease becomes evident. For chronic local infections in immunologic privileged sites, blood sample testing is generally unreliable. This applies not only to intraocular infections [95,335,336,337,338,339], but also to infections of the CNS or renal tubules. For example, in various animal species in which indications of shedding were detected by urinalysis (culture and/or PCR), very often, no antibody titer was detectable in the blood by the MAT [340,341,342]. Furthermore, it is important to note that in about 10% of 189 ERU horses from which *Leptospira* spp. were cultured from vitreous samples, MAT titers in the serum were negative (titer <1:100) [18,19]. MATs with serum are considered useful for assessing the degree of infestation and the infection pressure of a livestock, and thus also for identifying the risk of disease; therefore, it is not very meaningful for diagnostics in individual animals [18,19,342]. If anti-*Leptospira* antibodies are present in serum samples, one cannot be certain whether they are coincidental or related to an intraocular infection.

In a recent study, the detection of anti-*LipL32* antibodies with an on-site rapid test (SNAP Lepto, IDEXX company) has been shown to be a useful screening method to diagnose chronic intraocular leptospiral infection [308]. This SNAP Lepto is serovar and species non-specific, but it is a relatively inexpensive and easy to perform ELISA test that provides a result in 10 min. *LipL32* is present on the sample spot as well as in the conjugate in this rapid test; thus, any anti-*LipL32* antibody that has at least two binding sites can be detected. This test allows veterinarians with less experience in ophthalmology to perform blood tests in practice and thus check the probability of the presence of an intraocular leptospiral infection. Specificity, positive predictive value, and kappa-value with respect to the diagnosis of ERU are significantly higher for the SNAP Lepto than for the MAT when serum is used. However, although diagnostics have improved with the detection of antibodies directed against *LipL32* in serum, testing of intraocular specimens is still the most reliable for the detection of ERU [308].

## 7. Examination of Intraocular Specimens in Horses Suffering from ERU

To determine the etiology of uveitis, examinations of intraocular samples (aqueous or vitreous humor) are much more informative than blood tests [18,19,58,335,343,344,345]. Intraocular specimens can generally be examined for various antibodies, by means of PCR as well as cultures for various infectious agents. In addition, protein determinations, electrophoresis, and cytological examinations (e.g., in the case of suspected tumors) are also possible.

The collection and testing of aqueous humors can provide substantial information for the diagnosis of unclear intraocular inflammation. This applies to infectious and some noninfectious uveitis cases [346,347]. Therefore, in different species, the collection of aqueous humor, or even vitreous specimens and the analysis of these intraocular samples by laboratory diagnostics, is often an excellent way not only to assign the uveitis etiologically [348,349,350], but also to carry out the best therapy [16,345]. Correct indications and the adequate processing of ocular samples are decisive and determine the quality and reliability of laboratory results [346].

Extractions of aqueous humors are less invasive and associated with fewer complications than extractions of vitreous material, and aqueous humor examinations can provide satisfactory information for many questions [16,199,337,347,351]. Aqueous humors are also often informative for the diagnosis of posterior uveitis and allow for etiological diagnoses [19,337,347,351,352,353,354,355,356].

In human medicine, only about 0.1–0.2 mL of aqueous humor can be safely collected [344,345,346,357,358,359]. This is why the number of diagnostic tests that can be performed with this small aqueous humor volume is limited. However, if the history and clinical examinations have raised a certain suspicion, the low aqueous humor volume is often sufficient for a specific diagnosis [347,352,360]. If further laboratory tests need to be carried out and the small aqueous humor volume is not sufficient, intraocular sampling by vitrectomy can be performed to obtain a larger sample volume [351,353,361,362,363,364]. Approximately 0.5–0.7 mL [345,365], or even 1–2 mL [366] of undiluted vitreous humor, may be carefully aspirated at the beginning of a vitrectomy. More tests can then be performed, and there is a better chance of determining the etiologic diagnosis of uveitis.

In horses, on the other hand, 1 mL of aqueous humor can be collected safely, so that good preconditions are given to perform various laboratory tests [132,221,367]. The reports in which intraocular samples from ERU eyes were used are far fewer in number than studies using serum samples. One reason for this is probably that although the collection of aqueous humor has been described and is also considered a safe method under short anesthesia [132,221,222,367], many veterinarians hesitate to collect aqueous humor in still-seeing eyes and, in addition, some ophthalmologic experience is needed for paracentesis.

The number of intraocular specimens from ERU eyes examined in each case is also very low in some reports from recent decades. Sometimes, only 1 or 2 aqueous humor samples were examined [313,368]; in other reports, about 50 samples [316,328]; and in additional studies, the sample size was in between [14,306]. Some studies describe aqueous humor collection in horses under general anesthesia [132,221,222], but others describe the procedure as occurring under sedation and local anesthesia [14,369]. Some investigators differentiated the procedure and only performed anterior chamber puncture in eyes that were already blind in standing position, and otherwise under general anesthesia [328].

However, the collection of intraocular samples from equine eyes that have not yet suffered much damage is often difficult to justify if it does not result in a therapeutic consequence. For this reason, many of the intraocular specimens reported to have been examined in recent years were obtained from eyes that were blind and in the final stages of ERU. Intraocular sampling was then performed after the enucleation of blinded eyes or after euthanasia of the horses due to bilateral blindness [174,307,316,368,370]. By this time, the eyes show considerable atrophy or even phthisis and recurrent inflammation in the sense of ERU typically does not occur then. This means that intraocular samples from eyes in the final stage of ERU are no longer representative of the condition in the eye that existed during acute uveitis episodes. Reasons for the enucleation of blinded eyes include an overly exposed conjunctiva that is chronically irritated, leads to secretion and subsequent dermatitis, or an entropion that leads to persistent pain.

Reports of equine vitreous samples taken for diagnostic purposes are scarce; samples were mostly obtained after the enucleation, euthanasia, or slaughter of the horses [306,316,369]. It was not until vitrectomy was routinely performed, with vitreous material as a waste product of surgery and available for examination, that equine vitreous samples could be examined on a larger scale [16,18,19,66,69,315].

The greatest likelihood of detecting evidence of intraocular infection on the examination of intraocular specimens is generally whenever multiple testing procedures are used [345]. For example, when an aqueous humor sample is collected from horses for a preoperatively indicated diagnostic procedure, the sample should be examined as comprehensively as possible. Various antibody tests (MATs, in-house ELISA tests and SNAP Lepto) and PCR should therefore be performed routinely, because in some samples, antibodies are not detectable, but PCR is positive, and vice versa [19,66,68]. Since PCR has become available, the expensive and often prolonged cultural detection of leptospires in intraocular specimens is very rarely requested for routine diagnostics.

### 7.1. Breakthrough with Vitrectomy

Originally, the surgery was performed primarily to remove opacities from the vitreous cavity and to improve vision [282,283]. However, it very soon became apparent that after surgery, chronic recurrent inflammation also ceased in most eyes [67,68,284,286,287,288,289]. The increasing experience with the clinical and ophthalmoscopic examination and the correct indication, as well as the optimization of the operation itself—including the adaptation of the instruments to the horse eyes and the CO_2_ laser-assisted sclerotomy—made vitrectomy a relatively low-complication surgery with an astonishingly good prognosis. Indications for vitrectomy could be made with increasing precision, as examinations of numerous vitreous specimens and assignment of clinical findings to “*Leptospira*-positive eyes” have led to increasingly accurate classification of ophthalmologic findings [2]. This, in turn, has brought vitrectomy to the attention of horse owners and it has been requested with increasing frequency [16,191,288].

Due to the routine surgery of ERU-affected eyes, sterilely obtained vitreous material from thousands of eyes was available for various investigations. Vitrectomy was performed exclusively in the inflammation-free intervals of the disease [2,191]. Most of the eyes had suffered only two or three episodes of uveitis, and most of them were still visual [16]. Therefore, vitrectomy enabled the examination of numerous intraocular specimens from eyes that had little damage and were still in a florid stage of the disease, unlike the end-stage ERU eyes.

It was not until these numerous vitreous samples were examined that it became clear that a chronic intraocular leptospiral infection was the cause of ERU in almost all horses with typical clinical and ophthalmological signs [17,18]. In intraocular specimens obtained from vitrectomized eyes sometime after surgery (e.g., horses euthanized for colic, fractures, or other reasons), intraocular antibodies have been shown to continuously decrease over time postoperatively, and culture and PCR are then negative (Supplementary Material S4, Table S1 and Figure S25). It was concluded that the leptospiral infection is localized in the vitreous cavity, and that vitrectomy, in which only vitreous material is removed, also eliminates the infection [18,19,371]. The chronic leptospiral infection in the vitreous cavity explains why vitrectomy is so highly effective in horses [16].

Initially, the entire volume of liquid collected during vitrectomies was used for antibody and pathogen detection. Although vitreous material in the surgical lavage fluid was diluted approximately 10-fold, high antibody titers were often detectable using MAT [314]. In addition, 0.08 mg/mL gentamicin was added to the vitrectomy infusion solution for the prophylaxis of postoperative endophthalmitis [45,191]. In vitro, this concentration was 100-fold higher than the MIC for standard WHO strains of pathogenic *Leptospira* spp. [372]. Despite this, *Leptospira* spp. could still be cultured from gentamicin-containing lavage samples in some cases [373,374]. After optimization of the sampling technique, 2–3 mL of undiluted vitreous material could be collected at the beginning of surgery [2,191], which has significantly improved laboratory diagnostics with these samples, especially with regard to the culture of *Leptospira* spp. [19].

Other investigators did not use undiluted vitreous samples but collected the samples at the beginning of the surgery, so they were diluted as little as possible [66,67,68,288]. The varying results of the examination of the intraocular specimens (Supplementary Material S5, Table S2) can be explained through specimen collection during vitrectomy, further handling of the specimens, different laboratories with different serovars used for MAT, and the respective test procedures requested. Presumably for budgetary reasons, often not all available tests were applied. In addition, some laboratories declared low titers as “negative”, but did not take into account the specific knowledge from ophthalmology that any titer can be an indication of an intraocular leptospiral infection. However, a crucial factor is also the indication for vitrectomy (typical ERU) and the selection of suitable patients, because critical inclusion criteria play a decisive role for the success of the surgery [16] as well as for the intraocular fluid analysis [358].

Sometimes horses are referred for vitrectomy in which neither the history nor the ophthalmoscopic findings clearly indicate an ERU, and thus, an indication for vitrectomy. In these cases, it is indicated to first perform paracentesis of the anterior chamber of the eye (Section 4.1) and to test the aqueous humor for anti-leptospiral antibodies (MAT and ELISA tests) and for 16S rRNA and/or the *LipL32* gene. If one of the above tests is positive, there is an indication for vitrectomy [16,19,21,199]. On the other hand, if all tests come back negative, the indication for vitrectomy must be critically reconsidered.

SNAP Lepto for the on-site detection of anti-*LipL32* antibodies is not only suitable for the analysis of serum [308] (Section 6), but also for the analysis of intraocular samples [199]. The SNAP Lepto result is available after 10 min; therefore, the horse can be left under general anesthesia. If the SNAP Lepto result is positive, vitrectomy can be performed immediately. This avoids another recovery, which can cause injury to horses, and poses higher risks than general anesthesia itself. If the rapid test is negative, the horse must recover, and the aqueous humor sample is sent to an external laboratory for further diagnostics (MAT and PCR) [199]. If then in the aqueous humor either anti-*Leptospira* antibodies are detectable and/or the PCR is positive, vitrectomy must be performed under a second general anesthesia.

Since the investigations of vitreous samples obtained during vitrectomies had brought the leptospiral infection in ERU back into focus [17,18,19,45,314,373,374], and since the establishment of PCR for leptospiral diagnostics, investigations of intraocular samples with regard to leptospiral infection have also been performed by other working groups that did not have vitreous material obtained during vitrectomies at their disposal (Section 7.2, Section 7.3, Section 7.4, Section 7.5 and Section 7.6).

In parallel, vitreous material obtained during vitrectomies was used to intensively investigate the (auto)immune reactions accompanying the infection [55,154,158,159,165,192] (Section 3.1) and studies on drug transfer into the vitreous cavity could be performed without the need for animal experiments [196,299,375].

### 7.2. Antibody Detection in Aqueous Humor and Vitreous Samples

As for the examination of serum samples, most often, MAT was used for antibody detection for the examination of intraocular specimens [18,19,66,67,68,69,315]. In contrast to the examination of serum samples, MAT is highly specific when examining intraocular samples. The most common leptospiral serovar detected in equine eyes was *L. grippotyphosa*, but several other serovars from different serogroups can also cause ERU [19,20,66,69,315,376]. In about 90% of undiluted vitreous samples from ERU eyes, agglutinating antibodies are detectable by MAT (titer 1:100 or higher) [19,122,373]. Intraocular antibody titers often considerably exceed serum titers (Supplementary Material S6, Tables S3–S5 and Section 7.3). This phenomenon can also be observed without exact measurement of the titer when evaluating the SNAP Lepto: the sample spot is usually stained considerably weaker when examining serum compared with the examination of vitreous humor from the same horse [308] (Supplementary Material S6, Figure S26).

In a study of 724 serum samples from horses affected with ERU, the highest MAT titer measured in serum samples was 1:25,000 in a single horse (0.1%). In 426 vitreous specimens obtained at vitrectomy, the highest MAT titer was 1:3,267,800. In total, 62 of the 426 vitreous samples (15%) had an MAT titer of 1:25,000 or higher [18] (Supplementary Material S6, Table S4). However, when examining intraocular samples from healthy eyes, positive results in MAT or PCR are only found in exceptional cases (Section 7.11). Therefore, it cannot be ruled out that asymptomatic courses of intraocular leptospiral infection occur. However, it is possible that the *Leptospira*-positive eyes could also have developed ERU sometime in the future.

The sensitivity of leptospiral serology performed on intraocular samples from ERU eyes can be further increased by complementary ELISA tests. In addition to antibodies of immunoglobulin class G (IgG), IgA antibodies are of particular importance in intraocular infections [377,378,379,380,381], as well as in other local and especially biofilm-associated infections [382,383,384,385]. On rare occasions, in-house ELISA tests were used in addition to MAT, which were both immunoglobulin- and serovar-specific and enabled the detection of IgA antibodies, and were highly sensitive for the detection of an intraocular infection [194,195,198,386]. However, these tests are not routinely available. Examination of aqueous humor from ERU eyes in one study also revealed increased antibodies to LruC, a protein from the outer membrane of pathogenic *Leptospira* spp. [174]. However, to the best of the authors’ knowledge, no routine test is currently available for the detection of this protein.

In recent years, the serovar- and species-unspecific SNAP Lepto has proven its value for the examination of intraocular specimens (Section 6 and Section 7.1). When examining intraocular specimens, the sensitivity and specificity of the SNAP Lepto are equivalent to the MAT. SNAP Lepto and MAT provide predominantly, but not always, matching results when examining intraocular specimens [198,199]. High MAT titers correlate with a more intense blue coloration of the sample spot in the SNAP Lepto [198]. In individual intraocular samples (especially in very early ERU stages), only one of the two tests may react positively. The immune response of the individual patient, as well as the stage of the disease and different antibody dynamics (agglutinating antibodies versus anti-*LipL32* antibodies), may play a role here. Therefore, in case of a negative result of the first test, it is useful to perform another one for the detection of other anti-*Leptospira* antibodies [68,195]. Rarely, no antibodies at all are detectable in culture- and/or PCR-positive intraocular specimens [17,18,19,57,66].

In aqueous humor and vitreous humor of healthy eyes of humans and animals, normally, no antibodies are present [18,19,199,386,387] and their protein contents are very low (up to about 0.2 g/L) [43,119,189,192,344,388,389,390,391]. The average total protein content in samples from eyes affected with ERU in one study was 6.7 g/L which, although higher than that in healthy eyes, was, on average, only 1/10 of the total protein content of serum samples [18] (Supplementary Material S6, Table S3).

#### Blood–Aqueous Barrier and Intraocular Protein Content

Whenever the uvea is irritated, the barrier between the blood and the intraocular media (blood–aqueous barrier and/or blood–retinal barrier) is temporarily damaged and the passage of serum components or even cells into the eye may occur [132,158,345,392,393]. Some studies have described the collection of aqueous humor from acutely inflamed equine eyes [306,313,316,368]. In acute inflammation, the blood–ocular barrier is always disrupted, and intraocular samples contain more proteins than in the inflammation-free interval [132]. It should also be taken into account that, after euthanasia or enucleation, the blood–ocular barrier breaks down rapidly and the albumin content in aqueous humor and vitreous body multiplies within a short period of time. This can lead to falsified results when examining intraocular samples from euthanized horses or enucleated eyes [132].

Some authors point out that leakage from the serum into the eye persists for a long time after an acute uveitis episode has subsided [101]. However, in the event of leakage, the albumin content in the intraocular fluids would be expected to rise before the gamma globulin content. In fact, however, the albumin content in vitreous samples obtained during vitrectomies is comparatively low. In the serum samples, the albumin content is usually only slightly below the gamma globulin content, but in the corresponding intraocular samples, the albumin content is mostly very significantly below the gamma globulin content. Sometimes the gamma globulin content is several times greater than the albumin content. If, exceptionally, the albumin content is relatively high in an intraocular sample, the antibody titer measured therein is typically so high that it cannot be explained by leakage at all (Supplementary Material S6, Table S3).

Due to the highly effective clearance in the eye several days after trauma, no agglutinating antibodies are detectable in the aqueous humor of horses with traumatic uveitis and hyphemia, even if the serum of these horses reacts positively in MAT. In samples from eyes with a persistent, although slight disturbance of the blood–aqueous barrier (e.g., phacogenic uveitis, glaucoma, leopard coat pattern uveitis, and chronic heterochromic iritis), typically, no anti-*Leptospira* antibodies are detectable in the aqueous humor either, although MAT titers are present in the serum [15,16,18,19,45,69]. Furthermore, in horses suffering from ERU, the testing of corresponding serum and intraocular samples often reveals different serovars in MAT [18,45], so that even in these horses, no leakage may have led to the antibody titers in the eye (Supplementary Material S6, Figure S27). In horses in whose vitreous samples pathogenic *Leptospira* spp. could be detected by culture, the serovar (in the case of multiple serovars, the one with the highest titer) detected in the vitreous sample by MAT basically matched the serogroup of the culture. In contrast, MAT titers of other serogroups often dominated in the corresponding serum samples [18,20] (Supplementary Material S6, Figures S28 and S29).

Examination of numerous specimens from eyes affected with ERU has shown that antibody titers measured in MAT increase with the severity of ERU. In particular, the degree of diffuse vitreous opacification correlates positively with the titer level in intraocular samples [18]. In contrast, MAT titers are typically rather low in eyes with little damage, in which an aqueous humor examination may even be indicated before vitrectomy because no clear ophthalmoscopic changes are present and the indication for vitrectomy is therefore questionable. Based on decades of experience and thousands of test results, the authors meanwhile consider MAT titers of 1:50 in aqueous humor samples from these ophthalmoscopically unchanged eyes as “positive” and as an indication for vitrectomy, because the protein content in these eyes, just as in healthy eyes, is typically <0.2 g/L [43]. Thus, leakage with passage of antibodies from the blood into the eye can be almost completely ruled out.

Notably:

- Vitrectomy is performed exclusively in the inflammation-free interval, not during an acute attack of uveitis;
- A disturbed blood–ocular barrier especially plays a role during acute inflammation;
- Depending on the time of sampling and stage of ERU, the leakage of proteins from the blood into the intraocular fluids is of minor importance at the time of surgery (quiet interval and mostly an early stage of the disease);
- The immunoglobulin content in the serum is about ten times greater than the immunoglobulin content in the vitreous; nevertheless, the intraocular antibody titer is usually higher (Supplementary Material S6, Tables S3–S5);
- The more significant the diffuse haziness in the vitreous, the higher the protein content in the intraocular fluids, and the more obvious a “leakage” would be from the serum—but, in fact, the antibody titer in intraocular samples is then usually also significantly higher in relation to the antibody titer in the serum (Section 7.3) (Supplementary Material S6, Table S3);
- Low MAT titers in intraocular samples are mainly present when the eye ophthalmoscopically shows hardly any or no changes in the sense of ERU (especially no diffuse opacity of the intraocular fluids); then, the protein content in the intraocular samples is also very low;
- Electrophoresis shows that the eye usually contains less albumin than the immunoglobulin content would suggest; compared with serum samples, the albumin/immunoglobulin ratio in intraocular samples from eyes affected by ERU is shifted in favor of the immunoglobulin content;
- In “non-ERU” uveitis, glaucoma and a few days after damage of the blood–ocular barrier due to trauma with intraocular bleeding, typically, no intraocular anti-*Leptospira* antibodies are detectable even if MAT titers are detectable in corresponding serum samples.

### 7.3. Intraocular Antibody Production

Evidence for intraocular antibody production in ERU has already been provided by the extremely high MAT titers detectable in intraocular fluids described in the early studies (Section 3). At that time, the findings observed in horses served as a model for more sophisticated diagnostics in infectious uveitis in humans and the detection of local antibody production [394,395,396].

Intraocular antibody production is also supported by the detection of B cells in the uvea of equine eyes affected with ERU, which are not detectable in the uvea of healthy equine eyes [5,143,378,397]. In addition, the selective and marked increase in IgA content in samples from ERU eyes compared with corresponding serum samples suggests local antibody production in the eye [378].

The evaluation of intraocular antibody titers has been addressed several times since the middle of the last century and has been applied to various infections. In human medicine, toxoplasmosis plays an important role here [337,339,343,345,350,351,357,379,380,398,399,400,401,402], as well as viral infections [345,358,380,403,404]. The detection of intraocular antibody production for evidence of intraocular infection is easiest when intraocular antibody titers are higher than those in the corresponding serum samples, because in this case, intraocular antibody production is definitely present [313,395]. Otherwise, the intraocular titers could not be higher as in serum samples of the same individual. If the antibody titer in the eye is greater than the antibody titer in the blood, there may be intraocular antibody production, but it could also be a breakdown of the blood–ocular barrier and leakage of antibodies from the blood into the intraocular fluids. In this case, therefore, the protein content or another parameter in the paired aqueous humor and serum samples must be taken into account and set in relation to the respective antibody titer.

One possibility is to determine the total protein content in both samples [395]. Although the protein fractions have a different composition in the eye compared with the serum, the total protein value is still useful for calculations and is the simplest, cheapest, and fastest method which is easily available everywhere. Instead of total protein content, albumin content [344], total immunoglobulin content [335,343,395,400,403,405], total IgG content [344,350,380,401,404,406,407], or antibodies against another pathogen in the intraocular sample and in the serum can be used as reference [344]. Antibodies against the second pathogen must be present in the serum. If the ratio of the antibody titer in the intraocular sample to the antibody titer in the serum (titer intraocular/titer serum) is at least fourfold higher for the etiologically relevant pathogen in the eye than for the other pathogen, an intraocular antibody production is also proven. The most commonly used reference for antibody titers (specific IgG) is either the total globulin content or the total IgG content, if the laboratory techniques are available (e.g., electrophoresis or (in-house) ELISA tests).

#### 7.3.1. Calculation of the Goldmann–Witmer Coefficient (GWC)

When calculating the Goldmann–Witmer coefficient (GWC), it is sometimes misleading that the “antibody titer” should be used, but actually, the reciprocal titer should be inserted into the formula [18,19]. If this fact is not taken into account, “C” becomes smaller and smaller the higher the intraocular titer is. The calculation of the GWC would be correct as follows [18,19,335,343,344,345,391,400,408]:

(1) $$\begin{array}{cc} \mathrm{GWC} & =\left(\left(\mathrm{reciprocal}\;\mathrm{titer}\;\mathrm{intraocular}\;\mathrm{sample}\right)/\left(\mathrm{IgG}\;\mathrm{amount}\;\mathrm{intraocular}\;\mathrm{sample}\right)\right)\\ & /\left(\left(\mathrm{reciprocal}\;\mathrm{titer}\;\mathrm{serum}\;\mathrm{sample}\right)/\left(\mathrm{IgG}\;\mathrm{amount}\;\mathrm{serum}\;\mathrm{sample}\right)\right)\\ & =\left(\mathrm{reciprocal}\;\mathrm{titer}\;\mathrm{intraocular}\;\mathrm{sample}\right)/\left(\mathrm{IgG}\;\mathrm{amount}\;\mathrm{intraocular}\;\mathrm{sample}\right)\\ & \times \left(\mathrm{IgG}\;\mathrm{amount}\;\mathrm{serum}\;\mathrm{sample}\right)/\left(\mathrm{reciprocal}\;\mathrm{titer}\;\mathrm{serum}\;\mathrm{sample}\right) \end{array}$$

Instead of the IgG content, the total immunoglobulin content or, if necessary, the total protein content, could be used [395]. An example of the calculation could be as follows using the total immunoglobulin content if the intraocular titer is 1:100 and the serum titer is 1:200, and the serum immunoglobulin content is nine times greater than the immunoglobulin content of the intraocular sample:

(2) $$\mathrm{GWC}=\left(\left(100\right)/\left(4\;g\;\mathrm{per}\;L\right)\right)/\left(200/\left(36\;g\;\mathrm{per}\;L\right)\right)=\left(100\right)/4\times 36/200=4.5$$

#### 7.3.2. Interpretation of the GWC

In previous studies [335,343,395], intraocular antibody production was basically assumed at a coefficient >1 and then, to exclude errors and inaccuracies in the calculation, a coefficient of 3 [344] or >3 [400,404,408,409], respectively, was suggested as certain evidence for intraocular antibody production. In one publication, a coefficient of >8 was even required [189]; however, this has not prevailed. In more recent publications, intraocular antibody production is usually assumed from a GWC >3 [337,345,350]. When using the GWC, however, it must be noted that a high antibody titer in serum in combination with extensive breakdown of the blood–aqueous barrier may mask a positive coefficient, leading to a false negative result [345,400]. In addition, it should be noted that even with positive PCR or culture results, and especially in early stages of disease [380], sometimes there is no immune response resulting in intraocular antibodies [17,18,19,57,66]. Therefore, a negative GWC does not exclude intraocular infection [380,400,410].

When 426 undiluted equine vitreous samples obtained during vitrectomies and the corresponding serum samples were examined, the MAT titers in paired samples were compared without further breakdown of protein fractions. In 75.9% of the MAT titers determined parallel in serum and vitreous samples, the titer in the vitreous was higher than in the serum, so that intraocular antibody production was certainly present. In 10.3%, the MAT titers in the serum and the vitreous sample were the same which, with the average 10-fold higher total protein content in the serum, also supports intraocular antibody production, but would have to be verified in individual cases (e.g., with significant vitreous opacification) by calculation of the GWC. Only in 13.8% of the paired samples was the titer in the vitreous lower than in the serum. The mean MAT titer was 1:245 in the serum and 1:1680 in the vitreous sample [18] (Supplementary Material S6, Tables S4 and S5).

With 46 randomly selected corresponding vitreous and serum samples, electrophoresis was additionally performed. In 3 of the 46 paired samples, MAT was negative in the serum and positive only in the vitreous sample, so that intraocular antibody production was definitely present. In 7 of the 46 samples, MAT was negative with the vitreous material and positive only in the serum sample. Thus, no intraocular antibody production was present here. In the remaining 36 paired samples, an MAT titer ≥1:100 was detectable in both serum and vitreous, allowing calculation of the GWC. In 35 of the 36 paired samples (97%) the GWC was >3; in 34 of the 36 paired samples (94%) the GWC was also >8; and in 12 of the 36 paired samples (33%) the GWC was at least in the three-digit range, once even at 1792 [18] (Supplementary Material S6, Table S3). Thus, MAT titers are often several times higher in intraocular samples than in the corresponding serum samples and intraocular antibody production is regularly supported by demonstration of high Goldmann–Witmer coefficients.

#### 7.3.3. Misleading Interpretation of the GWC in Recent Publications on Equine Ophthalmology

In many recent papers describing the examination of equine intraocular samples, it appears that the γ-globulin fraction, the total globulin fraction, or the total protein value or any other reference value were not considered for the evaluation of antibody titers in the intraocular samples and the serum samples. The GWC is often cited, and the original paper is also referred to sometimes. The GWC and the so-called “C-value” are used synonymously. However, the calculation of the coefficient is not correct; simply, the intraocular titer is divided by the serum titer in each case [4,14,68,137,292,307,316,411]. The major problem with this simplified calculation is that it does not take into account the protein content, which in eyes affected by ERU is, on average, only 10% [18,19,391] or even less [390] of the protein content of the corresponding serum samples. Therefore, the result of this “C-value” does not equal the GWC. If the GWC is calculated correctly, intraocular antibody production can often clearly be detected even if the intraocular titer is lower than the serum titer (Supplementary Material S6, Table S3). By calculating the “C-value” without considering another reference value in the serum and intraocular fluid, the GWC is led ad absurdum.

The citations given for a calculation of the GWC, or “C-value” are often inappropriate or even wrong. In [14], for example, [412] is cited; in [410], however, no antibody determinations were performed in the intraocular samples. In [292], [67] is cited, but in [66], however, no antibody determinations were performed with the serum samples. For the calculation of the “C-value”, reference is often made to other studies in which the calculation is also not described, and further reference is made to other and often inappropriate papers (Supplementary Material S7, Figure S30). The impression arises here that some things have been adopted uncritically, too superficially, or even incorrectly from other studies, without the authors having thoroughly explored the GWC.

Finally, the “C-value” is considered “suggestive” or “suspicious” for intraocular antibody production in some papers with C > 1 [292,316]. In fact, however, the intraocular titer cannot be due to leakage from the blood at all if it is higher than the serum titer. Some publications consider C > 3 [292,316], C ≥ 4 [14,68], or even C > 4 [4,137,307] as confirmatory for intraocular antibody production. For this interpretation of the “C-value”, some papers are cited in which a consideration of the total immunoglobulin content or total IgG content is correctly described for the calculation of the GWC (e.g., [395,344，^350^]). The incorrect calculation of the GWC, in addition to the disease stage in which the sampled eyes were assayed not always being suitable (“endstage”), may be decisive reason why intraocular antibody production could only be detected in relatively few cases.

#### 7.3.4. Application of the GWC

Correct calculation of the GWC, when sampling from appropriate eyes and using a reference parameter in the serum and intraocular sample, reveals intraocular antibody production in the vast majority of paired samples from horses affected with ERU. The clinical diagnosis of “ERU” is very clear in most cases; therefore, a preoperative aqueous humor examination is not routinely required prior to vitrectomy when the risk/benefit ratio is considered but should only be performed when history and ophthalmologic findings do not clearly indicate ERU. In case of a preoperatively performed aqueous humor analysis, the GWC may be applied. Typically, however, these are eyes in a very early stage of disease, in which the GWC can be (falsely) negative despite the presence of the pathogen. Therefore, history, ophthalmologic findings, and GWC should not be evaluated in isolation, but always as a whole. In addition to antibody tests, PCR should be performed with the aqueous humor sample (Section 7.5), and culture of the pathogen (Section 7.4) may even be attempted.

### 7.4. Cultural Detection of Pathogenic Leptospira spp.

The detection of the pathogens in eyes affected by ERU was only sporadically successful (Section 3); therefore, the opinion had gained acceptance that leptospires are involved in the etiology of ERU, but that the recurrent inflammatory episodes and the anti-*Leptospira* antibodies present in intraocular specimens could no longer be explained by the presence of the pathogens. Thus, frustrating culture attempts were abandoned, or at least not reported, for some time.

Gaining convincing evidence of intraocular leptospiral infection was not possible until the establishment of vitrectomy in horses. With the vitreous material produced as a waste product of this operation, and the interest and commitment of S. Brem (head of the leptospiral laboratory in Oberschleissheim at the time), the collection and transport of samples could be optimized by successful cooperation. By taking 2–3 mL of undiluted vitreous material at the beginning of the vitrectomy and immediate sterile inoculation of vitreous material into a transport medium, pathogenic *Leptospira* spp. were cultured in increasing numbers of these undiluted vitreous samples [17,373,374]. One horse had suffered 21 apparently very well treated ERU episodes over 7 years (the eye was still visual), and in the vitreous sample from this eye, leptospires were still culturally detectable [18]. *Leptospira* spp. could also be cultured from vitreous samples from other horses that had suffered ERU relapses for many months or years.

With careful indication and optimized sampling, handling, and transport of vitreous specimens, leptospires could be culturally detected in >50% of vitreous samples [18,19,196,387]. This relatively high percentage of successful cultures of leptospires is surprising because, at the same time, anti-*Leptospira* anti-bodies were found by MAT in approximately 90% of these vitreous samples from eyes with ERU [17,18,19]. This means that the agglutinating antibodies had not resulted in the elimination of leptospires from the vitreous cavity and had not thwarted the culture of leptospires from these vitreous samples. Statistically, the chance for a positive culture was actually even greater the more substantial the diffuse vitreous opacity and the higher the MAT titer in the vitreous samples [18].

Since the turn of the millennium, other research groups have reported again on studies of intraocular specimens and the successful culture of leptospires from intraocular specimens [69,315,328,412]. The percentages at which the cultures were successful range from 16% to 21%. One reason for the varying results may be that, in some studies, aqueous humor samples were used for culture and the probability for a positive culture is lower when using aqueous humor compared with using vitreous material [18]. Some of the vitreous material obtained during vitrectomies was diluted, and gentamicin was added to the intraocular irrigation fluid [69], which also reduces the chance for a successful culture [45]. Additionally, the handling of the specimens, the timing, the sterile processing throughout, especially the inoculation into the culture medium immediately after collection, and the presence of suitable transport media for shipping play a very decisive role here, because leptospires are quickly overgrown by other bacteria and the culture can then not be set up further, but the positive culture is sometimes only detectable after months [374,413]. Finally, the indication for vitrectomy is handled inconsistently in different clinics and vitrectomy is not limited to typical ERU patients in all clinics [290].

Leptospiral serovars from different serogroups could be grown from ERU eyes, with the majority of isolates belonging to the serogroup Grippotyphosa [17,18,19,69,315,373,374,376] (Supplementary Material S6, Figures S28 and S29). Clinically, there was no detectable association between the course or severity of ERU and any particular serovar [18,19]. Therefore, the serovar causative of ERU appears to be determined primarily by the location of the horse and the vectors present there [58,59]. Uveitis has also been reported in humans following infections with various leptospiral serovars [89,90].

The demanding and expensive culture of leptospires, which often only yields a positive result after months, has become replaced in practice by PCR. Only for scientific questions, or if the owners want a stock-specific vaccine (Section 9), is the leptospiral culture still requested.

### 7.5. PCR

With the establishment of PCR for pathogenic *Leptospira* spp., another test method has been available since approximately the turn of the millennium, which is offered by almost all laboratories and is more economical than culture. In addition, sample handling and shipping are less complicated than for a culture of leptospires. PCR has proven to be a very sensitive and specific testing option for intraocular specimens [337,345,350,351,414]]. Finally, the results for PCR are available much faster and very small sample volumes are sufficient for an investigation. PCR has therefore significantly increased the informative value of the examination of intraocular samples in both human [337,346,351,352,356,360,366,407] and veterinary medicine [19,349,415] over the past decades.

In human medicine, the significance of PCR may be higher or lower than GWC depending on the type of infectious uveitis, the stage of disease, and the patient’s immune response. In immunocompromised human patients, PCR is superior, especially in diagnosing viral infections [416]. In toxoplasmosis, on the other hand, the detection of intraocular antibody production often plays a decisive role [416]. However, also in toxoplasmosis, other factors such as the age of the patients, immune status, duration since the onset of symptoms, and ophthalmologic findings influence the results of intraocular fluid sample examinations [417]. Especially in early disease stages, PCR may be more reliable than GWC. In principle, however, it is advisable to perform both the PCR and calculate the GWC in order to detect an infection as reliably as possible [337,345,350,402].

With the development of PCR for the detection of 16S rRNA and the *LipL32* gene of pathogenic leptospires, this meant a great improvement not only for the diagnosis of human leptospiral uveitis [91,94,207,327,418,419], but also for ERU. The demanding and often not very successful culture of leptospires had been replaced by PCR in practice and resulted in many positive detections in intraocular samples from horses suffering from ERU [16,66,68,69,195,199,328,369,412]. In approximately 70% of undiluted vitrectomy specimens from ERU eyes, *LipL32* genes or 16S rRNA were detectable by PCR [19,198,199].

In horses with ERU, PCR is not as sensitive as antibody detection in intraocular samples. Nevertheless, in some intraocular samples, only PCR reacts positively [19,57,66]; thus, PCR should be performed at least as a supplement if antibody tests are negative.

### 7.6. Comparison of Laboratory Results of Vitreous and Aqueous Humor Samples

Antibody titers in aqueous humor and vitreous samples are not significantly different from one another [18,221,222,420]. For the detection of intraocular anti-*Leptospira* antibodies in samples from eyes affected by ERU (about 90% positive), aqueous humor is easier to obtain and is of equal value to a vitreous sample.

Both cultural detections of leptospires and PCR detections of the *LipL32* gene or 16S rRNA are slightly less sensitive when examining aqueous humor as compared with vitreous samples [18,19,369], but diagnostic vitreous sampling would be disproportionately risky because the additional increase in information is not very large. In addition, PCR often yields positive results even when testing aqueous humor [18,19,222].

Therefore, the examination of aqueous humor is usually sufficient to diagnose ERU. In human medicine, it is also recommended to examine aqueous humor at least initially, because even in cases of posterior locations of inflammation, aqueous analyses often reveal an infectious cause [337,345]. For viral infections, it has also been described for PCR that aqueous and vitreous humor give comparable results [421].

Only in very rare cases, both antibody tests and PCR results of aqueous humor from ERU eyes are negative for leptospires. It is possible that in individual cases, the examination of a vitreous sample would then have yielded a positive result. If all tests are negative with aqueous humor, the eyes typically are still normal ophthalmoscopically (if there are clear findings in terms of ERU, examinations of intraocular specimens before vitrectomy would not be indicated anyway—Section 4.1 and Section 7.1). In these still largely healthy eyes, another consequently treated ERU episode is not expected to have vision-threatening consequences (Section 5.1). Especially in these ophthalmoscopically healthy eyes, taking a vitreous sample would not be justifiable with regard to the associated risks.

### 7.7. Ultrastructural and Histological Examinations of Vitreous Specimens

Vitreous material obtained during vitrectomies was processed for electron microscopy in a few studies. Transmission electron microscopy images reveal cells and vitreous structures from eyes affected by ERU [422,423]. These studies also succeeded in detecting leptospires in the vitreous samples [424]. When comparing the *Leptospira* spp. that had been present in eyes spontaneously infected with ERU and the *Leptospira* spp. from culture that had been experimentally injected into the eyes of euthanized horses, it was morphologically striking that only the leptospiral structures from the ERU eyes were coated by a granular layer (Supplementary Material S8, Figure S31). This layer was suggested as “masking” the bacteria from the immune system, possibly with host proteins.

In another study, phagocytosed leptospires were also visualized by transmission electron microscopy [425]. In this investigation, numerous very dense and roundish structures were present, which are not commented on in the publication (Supplementary Material S8, Figure S32). These round dense structures are morphologically very similar to the in vitro biofilm formation of leptospires and were exclusively detectable in vitreous samples from eyes affected with ERU; therefore, they were subsequently also discussed as biofilm structures. An older scanning electron microscopy image of a spherical structure with individual fibril-like structures protruding from it, some of which have hooks at the ends typical of leptospires, was retrospectively also discussed as a mature *Leptospira* biofilm structure [57,426].

Histological examination of enucleated eyes affected by ERU demonstrated that ERU leads to lympho-plasma cellular inflammation and the formation of lymphoid follicles [100,101,114,397]. B cell clusters have been detected in the center of these follicle [5,143,175], and the single-layered nonpigmented ciliary body epithelium contains numerous plasma cells, among others [427]. The lymphoid follicles and plasma cells are not present in healthy eyes and explain local antibody production in the eye. The ciliary body epithelium also contains hyaline membranes, the microscopic findings of which, along with the staining characteristics, indicated that the hyaline membrane has ambiguous morphologic features with qualities of both amyloid and collagen (12–13 nm filaments, Congo Red staining, spikes at 65 nm intervals, and blue staining with Masson’s trichrome stain) [427]. In another study, these membranes were classified as amyloid [224] (Supplementary Material S8, Figure S33).

Histological examinations of vitreous material are very rare. In “ERU-end-stage eyes” [370], the vitreous body is at least significantly altered or, as a result of phthisis, hardly or not at all present; thus, in these eyes, an examination of the vitreous body is not possible. In vitreous samples obtained during vitrectomies, amyloid has been visualized in the dense membranous floaters (Supplementary Material S8, Figures S34 and S35), which could be classified as AA amyloid by immunohistochemistry [428]. The fact that amyloid is detectable in the dense vitreous deposits in ERU eyes explains that these deposits are not resorbed, but increase over the course of ERU and become progressively visible on ophthalmoscopic examination during the clinically and ophthalmoscopically inflammation-free interval. In a recent study, for the first time, *Leptospira* spp. could also be clearly and reproducibly shown by immunohistochemistry in vitreous specimens obtained during vitrectomies [57] (Section 8.3). However, immunohistochemistry is unsuitable for routine diagnosis because it is extremely time-consuming and unreliable.

### 7.8. Cells and Cell Dynamics in Vitreous Specimens from ERU Eyes

In the mid-20th century, it was found that intraocular anti-*Leptospira* antibody titers measured by MAT decrease during the inflammation-free interval between uveitis episodes, and an acute ERU episode leads to an increase in intraocular antibody titers [115]. This is consistent with the fact that cytology studies of 74 undiluted vitreous specimens obtained during vitrectomies have shown that cell density in vitreous specimens is highest on the 8th to 16th day after acute uveitis and significantly decreases approximately 2 months after an acute episode [429,430].

However, even in the inflammation-free interval, there are always more cells present in eyes affected by ERU than in healthy eyes. The clinically quiet phase therefore does not mean an immunological quiet phase, but only a subclinical chronic inflammation. It has been suggested that the events between acute ERU relapses are controlled by T suppressor cells, which is clinically interpreted as an “inflammation-free interval” [429,430].

Independently of the time of sampling, the cell density in the vitreous samples has also been shown to correlate positively with the ophthalmologically determined degree of diffuse vitreous haziness [429,430]. Differentiation of cells in uvea tissues and vitreous samples from ERU eyes revealed that there were many lymphocytes and plasma cells in both the uveal tissue [134,143,200] and vitreous samples. In the vitreous samples, further characterization of the lymphocytes was repeatedly performed [169,193,431]. The presence of TH-1 cells, which increase the effectiveness of phagocytosing and cytotoxic cells [134], is also compatible with chronic intraocular leptospiral infection. In addition, autoreactive T cells are always present, which are activated by inflammatory reactions during infections (“bystander activation”) [210].

In eyes with high-grade vitreous haziness, it was noticeable that almost as many macrophages were present as lymphocytes. Plasma cells were seen relatively frequently in low-grade diffuse vitreous opacification. Cytological examination of vitreous samples was performed both after cytocentrifugation and after the embedding of macroscopically visible vitreous floaters. Regardless of the degree of vitreous haziness and the degree of vitreous floaters, the percentage of lymphocytes was significantly higher after cytocentrifugation (cytological examination) than in the histological examination. In contrast, the percentage of macrophages and plasma cells was significantly higher in the histological examination than after cytocentrifugation. It can be concluded that lymphocytes are mainly contained in the liquid phase of the vitreous, macrophages, and plasma cells rather than in the denser vitreous deposits which are visible ophthalmoscopically, and after vitrectomy, also macroscopically in the samples. These findings may indicate that leptospires primarily reside in the denser vitreous deposits [429,430].

### 7.9. Neutrophil Extracellular Traps (NETs)

Another phenomenon detected in vitreous samples from eyes affected by ERU is the presence of neutrophil extracellular traps (NETs) [432]. NETs are externalized DNA fibers from neutrophils which contain, among other substances, cytoplasmic proteins, chromatin, and antimicrobial peptides [433,434]. Scanning electron microscopy revealed that NETs contain smooth stretches with a diameter of 15–17 nm, globular domains of around 25 nm, and aggregated fibers with diameters of up to 50 nm [435].

NETs are able to entrap and kill bacteria [435] and other pathogens [436]. NETs are believed to be part of the innate immune system [437]. They can occur in all organs and are abundant at sites of acute inflammation [438].

NETosis refers to an active cell death of neutrophils, which perish with the release of NETs (“active cell death”) but can still kill bacteria through the excreted NETs even after their cell death [439]. However, it has been shown that neutrophils can also form NETs without dying in the process [440]. Some neutrophils only secrete mitochondrial DNA, sometimes but not always resulting in cell death [441,442]. Meanwhile, distinctions are made between “suicidal NETosis”, “vital NETs”, and “mitochondrial NETs” [441]. Components of NETs are non-specific, and a variety of stimuli can induce NET-release [443]. Among others, infections (e.g., presence of LPS) and pH changes (e.g., in inflammation) can induce the release of NETs and NETosis [438].

The antimicrobial potential of NETs is positive for the host. However, because NETs can also cause inflammation and tissue damage [441], not every minor milieu change or small amount of LPS should lead to the release of NETs [440]. The formation of excessive NETs and their tissue-damaging effects, caused both directly and via accompanying inflammatory reactions, have also been linked to the development of various autoimmune diseases [438,441,443,444].

Most impressively, it has been shown that neutrophils can sense the size of microbial antigens. For smaller antigens that can be phagocytosed, tissue-damaging NET formation is suppressed. In contrast, for larger pathogens that cannot be phagocytosed, NETs are released [436]. If, as suspected, the roundish structures on the electron micrographs of vitreous specimens from eyes affected with ERU (Supplementary Material S8, Figure S32) are indeed leptospiral biofilm, these structures would undoubtedly be too large for phagocytosis (“frustrated phagocytosis”). This would perfectly explain the NETs in the eyes affected by ERU. In addition, NET formation promotes an increasing inflammatory response, the inflammation attracts further leukocytes, and this, in turn, promotes tissue damage [445].

NETs, on the other hand, can also counteract inflammation and tissue damage. They eliminate pathogens and serve as a physical barrier that not only prevents further spread of bacteria or other pathogens, but also prevents tissue-damaging substances (e.g., proteases) from making contact with the host tissue [435]. In the areas of the conjunctiva and cornea, aggregated neutrophil extracellular traps prevent inflammation on the neutrophil-rich ocular surface [434,446]. In corneal infections with biofilm-forming *Pseudomonas aeruginosa* spp., NETs separate the biofilm from healthy tissue, for example, and thus work against further expansion of the infection [447]. Possibly with respect to the delicate balance in ocular immunity, “ocular neutrophils” are supposed to be less inflammatory compared with neutrophils in the blood [446]. This may explain why there are clinically inflammation-free intervals in ERU despite the presence of *Leptospira* spp. and NETs in the vitreous cavity.

Another interesting point is that the biofilm formation of bacteria and the formation of NETs promote one another. The bacteria “barricade” themselves in the biofilm, to be protected from the host immune system. The neutrophils, on the other hand, produce NETs to create a barrier between the biofilm and host tissue [447,448]. Fibrous material such as NET and fibrin is suggested to prevent the clearance of bacteria by phagocytic cells, and thus promotes the growth of bacterial clusters or biofilms. Fibrin and NETs are discussed to be important for the recurrence and chronicity of otitis media in children [449].

### 7.10. AA Amyloid

Serum amyloid A (SAA) is generally an indicator of chronic inflammation and infection, and its determination in blood is now part of routine diagnostics. In horses, SAA is considered to be the most sensitive and the only major acute-phase protein. The SAA content can increase by a factor of up to 1000 in the case of tissue trauma [450]. SAA increases within a few hours and also decreases rapidly when the inflammatory response decreases [451]; therefore, SAA measurements are an excellent method to quickly check the response to a therapy or antibiotic, for example.

In horses suffering from ERU, it has been shown that the eye disease has no effect on the SAA content of the blood [452,453,454]. In a recent study, this fact was also described for joint infections in horses [455]. Thus, local infection at immunoprivileged sites does not reliably affect blood SAA levels.

In contrast, examinations of intraocular samples showed that the SAA content in the vitreous samples obtained during vitrectomies of horses suffering from ERU is significantly increased and the level of MAT titer in the vitreous samples correlates significantly positively with the SAA content. In horses with leopard coat pattern uveitis, in which anterior uveitis dominates the clinical picture, an increased SAA content was detectable, especially in aqueous humor, but hardly in vitreous samples [453,454]. On the other hand, a significantly increased SAA content in vitreous samples indicates an ERU. Thus, unlike the comparable results in aqueous humor and vitreous samples when antibody titers are measured, SAA does not appear to yield comparable results in both compartments. The concentrations of SAA in eyes exhibiting non-uveitic related changes (e.g., keratitis, tumors, and ERU partner-eyes) were generally below the detection limit [453,454].

Sustained overproduction of SAA is a prerequisite for the development of AA amyloidosis [456]. AA amyloid has been detected in intraocular specimens from ERU eyes—both in the ciliary body area and in the “snowbanks” located on the ciliary body [224,427,457], and in the dense vitreous floaters of ERU eyes [428] (Supplementary Material S8, Figures S33–S35).

AA amyloidosis can have different etiologies. A major cause is chronic infection [458,459]. The amyloid deposits that develop as a result of chronic infections may occur generalized or localized [459]. In generalized AA amyloidosis, various organs are often affected (e.g., the kidney, liver, spleen, and gastrointestinal tract) and renal insufficiency is a particularly feared consequence in humans [456,459]. The most effective therapy is to eliminate the cause of chronically elevated SAA levels because treating the underlying disease can lead to subsequent reductions in acute-phase protein production, including the circulating serum SAA levels. However, there are no common therapeutic strategies for AA amyloidosis due to its diverse etiology [458].

There is no evidence of generalized amyloidosis in horses affected with ERU. AA amyloid has exclusively been detected intraocularly to date [428]. Intraocular amyloid formation also explains why, although diffuse opacification decreases in the inflammation-free interval of ERU, the cloudy or membranous vitreous floaters which are visible ophthalmoscopically increase over time and do not resolve (Supplementary Material S1, Figure S13 and Supplementary Material S8, Figures S34 and S35).

These ophthalmoscopically visible vitreous floaters, which can most reliably be seen using a direct hand-ophthalmoscope, provide strong evidence of ERU in non-leopard coat pattern horses during the inflammation-free interval when the eyes are otherwise still relatively undamaged. Even if there is no anterior uveitis or its sequelae and if no or hardly any diffuse vitreous haziness is present, these dense and therefore vitreous floaters which appear dark are easy to recognize in the ophthalmoscopic view [18,28,217].

However, in chronic infections, amyloid does not exclusively result from persistently elevated SAA levels. Chronic infections are often associated with biofilm formation. Various biofilm-forming bacteria, especially enterobacteriaceae such as *Escherichia* coli [460], are able to produce microbial amyloid themselves. Amyloid is found in many naturally occurring biofilms, and microbial amyloid can provide a support scaffold for biofilms [461]. In fact, amyloid fibers are actually found in most naturally occurring biofilms [462]. Microbial amyloid can occur in the form of fibrils and, in the case of aggregated fibrils, also in the form of fibers [462,463]. These functional amyloid fibers (FAPs), which include “curli fibers”, support the binding of bacteria to host proteins and promote biofilm formation [461,464]. In addition, the FAPs protect the bacteria in the biofilm from phagocytosis [465].

No FAPs were seen in in vitro studies of *Leptospira* biofilm formation [466], and to the best of the authors’ knowledge, nothing is yet known about whether *Leptospira* spp. are able to form FAPs in vivo. Independently, however, leptospires in the vitreous cavity might take advantage of intraocular AA amyloid generated by immune reactions for their biofilm scaffold. The amyloid fibrils and fibers, similarly to the vitreous collagen fibrils and fibers, could serve as surfaces for the attachment of *Leptospira* spp. and thus promote further biofilm formation as well as provide protection from the host immune system.

### 7.11. Samples from Healthy Eyes

Samples from clinically and ophthalmoscopically healthy equine eyes usually do not show any signs of intraocular infection with *Leptospira* spp. [17,18,45,195,199,314]. Systemic leptospirosis typically leads to clinical manifestations in the sense of ERU only after months or years; therefore, evidence of leptospiral infection can be expected in individual cases even in clinically healthy eyes. The incidence of ERU in some regions of Germany was 8–10% [9,10]; thus, in individual cases positive MAT and/or PCR results in intraocular specimens from apparently healthy eyes can be expected if enough specimens are examined.

In individual studies, specimens from anamnestically and ophthalmoscopically healthy eyes or in eyes that had disease other than uveitis were examined. An examination of specimens from 366 healthy eyes failed to detect anti-*Leptospira* antibodies by MAT in any of the intraocular specimens [122]. In another study, samples from 168 healthy eyes were examined and anti-*Leptospira* antibodies were detectable in only 1 sample (by both MAT and in-house ELISA). Culture yielded a negative result in all 168 sets. A total of 120 of these samples were additionally tested for 16S rRNA by PCR, and in this assay, 6 of the samples (5%) reacted positive. Control samples from 14 ERU-affected eyes all had anti-*Leptospira* antibodies by both MAT and in-house ELISA, and with 8 of the 14 samples (57%), the culture was positive [386,387].

In another study, no culture was attempted, but no MAT titers of 1:100 or higher were obtained in any of the 120 intraocular samples (100 vitreous samples and 20 aqueous humor samples), whereas a positive PCR result (detection of the *LipL32* gene) was obtained in only 1 of the 120 intraocular samples [199].

The samples from healthy eyes were taken for ethical reasons only after euthanasia of the [467,468] horses or after enucleation (for another reason than ERU); thus, the eyes could not be observed further. It is therefore unknown whether the eyes from which the samples with positive MAT or PCR results were taken would have developed ERU in the course of the following months and years, or whether it had been an asymptomatic infection.

## 8. Bacterial Biofilm

Individual (planktonic) bacteria are relatively unprotected and vulnerable to an attack. When it becomes “uncomfortable” for the bacteria, bacterial aggregates form, which can barricade themselves in a matrix and thus survive under adverse environmental conditions, both inside and outside a host. Biofilm is generally defined as “an aggregate of microbial cells surrounded by a self-produced polymer matrix” [469]. There are both mono- and polyspecies biofilms. Both in low-nutrient environments and in the presence of unfavorable factors harmful to bacteria (e.g., pH, low temperature, lack of nutrients, oxygen tension, UV light, antibiotics, host immune system, etc.), the biofilm represents an effective protective barrier [470]. Biofilms not only confer protection, but also exhibit a potential for homeostasis, thereby reducing maintenance energy [460]. The in vivo biofilm can also harness certain immune responses of the host and integrate them into the biofilm scaffold (e.g., NETs and fibrin fibers) [449].

Bacteria in biofilm are relatively tolerant to antibiotics which is not only due to the mechanical barrier that the biofilm represents; the ability of the antibiotics to diffuse through the biofilm depends largely on the particular bacterial strain, the type of antibiotic, and the growth conditions of the biofilm [471,472].

Depending on the pathogen species and the type of biofilm, it could be shown that the bacteria, as long as they are hidden in the biofilm, have a tolerance to antibiotics up to 1000 times higher than the same planktonic pathogens [473,474]. After detachment from the biofilm, the planktonic bacteria again exhibit normal sensitivity to antibiotics. For this reason, the term “tolerance” is preferred for bacteria in the biofilm, because the planktonic bacteria, after leaving the biofilm, exhibit the original sensitivity to antibiotics again and have thus not developed “resistance” [475].

The first observations of biofilms, even though this term did not exist at the time, were described in 1684 by Anthony van Leeuwenhoek for dental plaque [476,477]. The term “biofilm” has been used for about 60 years [476]. Reports of aggregates of pseudomonads classified as biofilm in cystic fibrosis (CF) patients [478], initial [479] and later updated [480] definitions of biofilm, and implications of biofilm formation have indicated the importance of infections associated with biofilm formation.

The growth of bacteria in biofilm initially appeared to be tied to surfaces, but it was later shown that attachment to organic or inorganic surfaces is not an absolute requirement for biofilm formation [477]. Biofilms can harbor different infectious agents simultaneously [481]. Interestingly, bacteria are able to adjust their metabolic processes to their environment. Bacteria within a biofilm show changes in gene expression, resulting in phenotypic heterogeneity within the biofilm [467,482], which can be interpreted as a specialization or division of labor [483].

For the regulation of processes in biofilms, cell-to-cell communication (quorum sensing, QS) takes place [482,484,485]. QS is made possible by signaling molecules, which are secreted to coordinate community behaviors and to defend against unfavorable environmental conditions [467,468]. Within the biofilm scaffold are water-filled channels through which nutrients, metabolites, and other secreted substances can be transported [466,485], and through which QS is performed [483,484,485]. QS and the resulting adaptation of the secretion systems of bacteria, as well as their expression levels of genes, are important for cells to adjust to their environment. In this way, the biofilm matrix can become more impermeable to antibiotics [468,472,486,487].

For an increasing number of infections, it has been recognized that biofilm formation of the causative pathogens is a major problem for sustained successful therapy; often, the infection appears eradicated with antibiotic treatment, but shortly after discontinuing therapy, the infection may recur because bacteria have survived in the biofilm. For infections, biofilm formation was defined as “A coherent cluster of bacterial cells imbedded in a matrix—which are more tolerant to most antimicrobials and the host defense, than planktonic bacterial cells” [476]. It is now believed that almost all bacteria can form biofilms [484,488] and that 60% of all infections and 80% of chronic infections in humans are biofilm-associated [485,489]. Biofilms in dental plaque [490,491,492] and in CF [385,482,493,494] have been particularly well investigated.

Other biofilm-associated diseases include wound infections [495,496], infected implants and bone sequestrum formation [497], osteomyelitis [498,499,500], chronic otitis [449,501], infectious endocarditis [502], and borreliosis [503,504,505]. Whenever possible, mechanical or surgical removal of the biofilm is considered the most effective therapy to eliminate the infection [480,506,507]. When mechanical or surgical removal is not possible, it is critical for successful therapy to know not only the environmental and in vitro biofilm formation, but more importantly, the in vivo biofilm formation and microenvironment of the infection, because the differences can be significant and crucial for successful therapy [508,509].

Depending on the phase of biofilm formation, different treatment strategies have been proposed [510], such as substances dissolving the biofilm matrix [489,511] or disrupting QS within biofilms [512,513,514]. Other approaches to attack biofilms include ultrasound-targeted microbubble destruction under antibiotic administration (UTMD) [515], targeting extracellular polymeric substances (EPS) (inhibiting EPS production or degrading EPS), or the use of bacteriophages [468].

### 8.1. Steps of Biofilm Formation and Dissemination of Infection

After bacteria have come into contact with a surface or with other bacteria and irreversible adhesion has occurred, cell division begins so that aggregates of many bacteria are formed. At the same time, EPS are secreted, which initially form a young biofilm matrix, and later also constitute a major part of the biofilm matrix. The biofilm matures further to a three-dimensional and well-organized entity. By means of QS, the biofilms mature in such a way that cell density and composition of the matrix are adapted to the respective environment of the bacteria. The matrix is continuously adapted to changing environmental conditions by QS [460,475,483,485,509,516]. Some bacteria stabilize the biofilm scaffold by self-produced amyloid fibers (curli fibers) (Section 7.10). In addition, fibrin fibers, NETs and other structures can be integrated into the biofilm scaffold (Section 7.9).

The host’s immune defenses often struggle unsuccessfully to eliminate the bacteria and biofilm. Antibiotics are also often unable to completely eradicate the infection. However, even if the elimination of the infection does not succeed (e.g., “frustrated phagocytosis” [383,445]), phagocytic enzymes are released, causing tissue damage around the biofilm [480]. Spread of infection within an organ or throughout the host can occur further along, both by individual planktonic bacteria emerging from the biofilm and by shedding of biofilm parts [480].

One cause that leads to the release of bacteria from the biofilm or the detachment of parts of the biofilm is an accumulation of metabolites in the biofilm which are toxic to the bacteria within the biofilm. At some point, this leads bacteria within the biofilm to start the self-dissolving program by secreting EPS-digesting hydrolases [468]. In particular, areas located at the periphery of the biofilm appear to break open after bacterial proliferation and production of saccharolytic enzymes, contributing to dissemination of the infection [485]. External influences that are favorable to the bacteria, such as a rise in temperature [517] or a more nutrient-rich environment [518,519], are also perceived by the bacteria in the biofilm and can trigger the release of bacteria from the biofilm. The planktonic cell–biofilm cycle explains the chronic recurrent course of inflammation in biofilm infections [468].

Considering the difficulties in eliminating biofilm-associated infections, prophylaxis is of great importance. In this context, the prophylactic administration of antibiotics during certain operations appears in a different light, because the infectious agents can be eliminated at an adequate level of action before biofilm formation can even begin [475,511,520].

### 8.2. Characteristics of Biofilm-Associated Diseases

Various characteristics of biofilm infections have repeatedly been described. The following points are particularly significant [385,508,521,522]:

- The direct examination of infected tissue reveals bacteria living in cell aggregates or microcolonies surrounded by an extracellular matrix;
- The infection is generally confined to a specific location or organ;
- The infection is impossible or difficult to eliminate using antibiotics, to which the responsible organisms are sensitive when in their planktonic or free-living state;
- Often, no organism can be cultured despite a strong presumption of infection with the pathogen of interest;
- Immune responses are ineffective, as evidenced by bacterial aggregates surrounded by inflammatory cells within host tissue.

In CF, the detection of specific IgG antibodies in the blood has been shown to be useful in the diagnosis of pneumonia, whereas the detection of specific IgA antibodies in the secretion has been shown to be valuable in the diagnosis of sinus infection [384,494]. Similarly, the detection of specific IgA antibodies in specimens from eyes affected with ERU is particularly sensitive for the detection of chronic intraocular leptospiral infection [194,195]. Apart from intraocular infections, chronic leptospiral infections have been described, especially for proximal renal tubules, and have also been associated with biofilm formation [523,524,525,526]. In cattle, the detection of urinary IgA specific for leptospires has recently been described as a “hallmark” to identifying reservoir hosts [527].

Further characteristics depend on the particular pathogen and the localization of the infection. In CF, for example, alginates have been described as a particularly immunogenic component of the *Pseudomonas aeruginosa* biofilm. In contrast, studies of a *Leptospira* biofilm grown in vitro could not identify alginates as a major component [466].

Biofilm-associated infections cause chronic inflammation in the tissue surrounding the biofilm and can also initiate autoimmune responses [528,529,530,531]. Inside the eye, any disturbance of the immunologic balance leads to autoimmune reactions anyway. Thus, the intraocular biofilm formation of leptospires is a good explanation for the infection-associated autoimmune phenomena detectable in ERU.

### 8.3. Biofilm Formation of Leptospira spp.

Biofilm formation by leptospires has gained interest for about 20 years. *Leptospira* spp. have been detected in dental care unit pipes [532], and it was found that *Leptospira interrogans* serovar Canicola can survive in distilled water for more than 3 months, whereas in a viscous medium, they can survive for over three times as long—whereby the viscous medium had clearly favored the formation of cell aggregates within 48 h [533].

Biofilm formation was observed in vitro in distilled water (low-nutrient medium) in different *Leptospira interrogans* strains [519], and morphologically demonstrated in different serovars and strains of pathogenic *Leptospira* spp. [534]. Meanwhile, further details of the morphology and composition of the leptospiral biofilm formed in vitro (*Leptospira interrogans* serogroup Pyrogenes serovar Manilae) are known [466]. In vitro studies of apathogenic and pathogenic leptospires have shown that aggregates of leptospires form within 1 day, and that biofilm formation begins within 2 days. Biofilm formations over 100 µm thick can develop within 3 weeks [466,534]. The ultrastructure of the three-dimensional biofilm, consisting of, e.g., *L. interrogans* with the organizational features therein (e.g., transport channels), is quite impressive [466].

In in vitro biofilms of *Leptospira interrogans* serovar Laiit, it could be shown that after the addition of nutrients (rabbit serum), free motile bacteria were increasingly found again over the course of 2 h [519]. Thus, more favorable environmental conditions (here: the addition of nutrients by rabbit serum) seem to be a signal for leptospires to leave the biofilm. It has also been shown for different pathogenic *Leptospira* spp. that the bacteria within the biofilm are more tolerant to antibiotics than the planktonic forms [535], although protection by the biofilm appears to be less effective compared with what has been described for *Pseudomonas aeruginosa* (Section 8).

In environmental multispecies biofilms obtained from rice fields, the *LipL32* gene of pathogenic leptospires was detected among other bacteria. Plant fibers in the standing waters of rice fields are surfaces where biofilm formation is favored [481]. There are also descriptions of environmental leptospiral biofilm formation that occurs in soil, which may, in addition to rats and mice, contribute to the numerous human cases of leptospirosis following floods [536,537]. *Leptospira interrogans* serovar Icterohaemorrhagiae can remain infectious for at least 20 months under unfavorable environmental conditions [538], which could be due to biofilm formation. Thus, *Leptospira* survival in the environment seems to be favored by biofilm formation, which could contribute to disease transmission not only after environmental disasters, but also among risk groups such as sewage and agricultural workers [518,533,539].

In environmental biofilms, the coaggregation of leptospires and other bacteria has been observed. These coaggregates have been discussed to be a key factor for the persistence and survival of *Leptospira spp.* [540]. Furthermore, the observed coaggregation of different Leptospira strains and *Staphylococcus aureus* might be important for leptospiral infections via skin lesions [541].

In vivo biofilm formation of leptospires in the proximal renal tubules of chronic shedders has been suspected for some time [523,534]; it has been demonstrated by electron microscopy after experimental infections [524,525], and recently in naturally infected *Rattus norvegicus* [526]. Experimental infections of mice were used to understand the in vivo immune evasion [542]. Another experimental study demonstrated that antibiotics administered within 1 day of infection work well, but the elimination of leptospires with antibiotics 3 days after infection is considerably more problematic, which was linked to biofilm formation in the proximal renal tubules [543].

**Figure 1:**
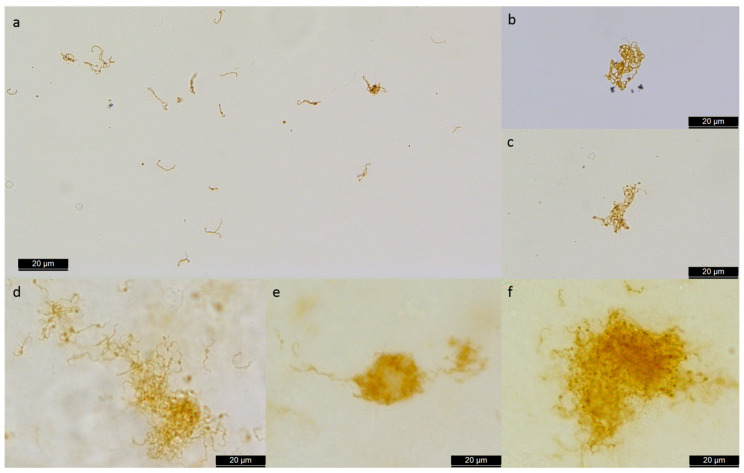
Immunohistology (IHC) with a vitreous sample from an ERU eye. IHC was performed with a polyclonal rabbit anti-*L. grippotyphosa* antibody from the Amsterdam UMC Leptospirosis Reference Centre. The method used was described in [57]. All photographs in this figure are from different slides from the same vitreous sample, which was PCR-positive for *LipL32* and Subclade P1 (16S RNA) and was MAT-positive (1:3200 *L. grippotyphosa*). (**a**) Single *Leptospira* spp., (**b**,**c**) small *Leptospira* aggregates, and (**d**–**f**) large aggregates of *Leptospira* spp. and the extracellular matrix around and in between the bacteria. The extracellular matrix indicates biofilm formation. Especially in images (**c**,**d**,**f**) there seems to be abundant membrane blebbing. (Photographs: K.A.).

Biofilm formation by leptospires as a pathogenic mechanism in ERU has been suspected in recent years [21,426,544], and it could also be demonstrated morphologically [57,545] (Figure 1). Previous work that did not yet use the term “biofilm” also showed evidence of leptospiral biofilm formation in vitreous samples from eyes affected with ERU during ultrastructural studies [424,425] (Supplementary Material S8, Figures S31 and S32).

Leptospirosis as a biofilm infection seems to be of great contemporary interest and has been investigated by different research groups. In particular, biofilms produced in vivo by leptospires need to be further analyzed and could yield interesting results. Presumably, the composition of biofilms produced in vivo differs significantly from biofilms produced in vitro, which may be crucial for their elimination.

### 8.4. Vitreous Structure, ERU, and Biofilm

The vitreous, at least in the horse, represents an ideal site for the biofilm formation of leptospires [426,544]:

- It consists of 98–99% water [11,546];
- Hardly any nutrients are present in the healthy vitreous body (Section 7.2);
- The collagen fiber scaffold in the vitreous body could serve as a surface, analogous to plant fibers [481], where biofilm formation begins; the vitreous fibrils in the horse [422] have a diameter of approximately 10–12 nm, as in other species [547], and several fibrils can attach to each other to form fibers [546];
- Some vitreous areas are very viscous (hyaluronic acid and collagen fibers), which also promotes biofilm formation [533];
- The equine vitreous represents an avascular “immunological niche” of approximately 28 mL [11], resulting in delayed and reduced host defense responses; thus, biofilm formation can occur before the immune defense has any chance to eliminate the infection;
- Intraocular immune privilege: the eye attempts to limit local immune and inflammatory responses to preserve vision (and thus enables antigens and histo-incompatible tissue to survive longer in the eye) [548,549], which also contributes to the delayed and insufficient elimination of leptospires;
- As in the renal tubules, there are fenestrated capillaries in the area of the Pars plicata of the ciliary body under the respective single-layered unpigmented and pigmented epithelium [5], which can promote passage of the leptospires into the vitreous cavity during hematogenous infection.

In the course of ERU, immune reactions occur, which are individually very different, but typically lead to the invasion of cells (Section 7.8), intraocular antibody production (Section 7.2 and Section 7.3), and various autoimmune phenomena (Section 3.1). In addition to the physiological collagen fiber scaffold, further fibrils and fibers are formed in the vitreous body, which, in turn, can promote biofilm formation:

- Fibrin: the fibrin scaffold looks very similar to the collagen fiber scaffold of the vitreous body by electron microscopy: see picture p. 10 in [550] (https://publications.rwth-aachen.de/record/466223/files/466223.pdf; accessed on 5 December 2021);
- NETs (Section 7.9, vivid picture on: https://scitechdaily.com/are-overactive-immune-cells-the-cause-of-COVID-19-deaths/; accessed on 5 December 2021);
- AA amyloid: amyloid fibrils are 8–15 nm in diameter [551], similar in thickness to those of the vitreous scaffold, and amyloid fibrils can also assemble into fibers; the curli fibers formed by some bacteria in the biofilm (Section 7.10) are only slightly thinner (6–12 nm in diameter) [552].

It is conceivable that this network of fibrous material becomes integrated into the leptospiral biofilm, making pathogen elimination increasingly impossible, similar to what has been described for chronic recurrent otitis in children [449]. It is therefore clear why vitrectomy is the most effective treatment option for typical ERU. Mechanical, or in this case, substantial surgical removal of these biofilm-fiber conglomerates is the only way to reliably eliminate the infection. In eyes, the infection is eliminated only in the context of high-grade atrophy of the globe or phthisis; then, the space of the vitreous chamber is considerably reduced and physiological conditions (e.g., the “immunological niche” and healthy or viscous vitreous material) are no longer present in the eye.

## 9. Prophylaxis

Regarding the two decisive triggering factors of ERU, leptospiral infection and genetic predisposition, starting points for a reasonable prophylaxis are also recognizable. Forty years after the Royal Commission for Horse Breeding banned stallions with cataracts from breeding, the incidence of ERU in the United Kingdom has dropped significantly [50]. Thus, with increasing knowledge of the genetic “make-up” associated with the incidence of ERU, further breeding selection would be prudent.

In addition, attention should be paid to the prophylaxis of infection. The higher the infection pressure in a herd, the higher the probability that many horses will become infected, and later, some of these horses will develop uveitis (Section 6). Thus, if possible, control of free-living small mammals should be implemented in the barn and its surroundings. If possible, grazing should take place on pastures that are as dry as possible and watering from stagnant water should be avoided in order to reduce the risk of infection.

Horses themselves can also shed leptospires after infection (Section 2), and could therefore also be a source of infection for other horses, at least temporarily. Thus far, this source of infection is generally considered to be of minor importance, and because of the predominantly subclinical course of acute leptospirosis in horses, excretors would only be detected by regular urine tests. However, this would be costly and time-consuming for horse owners and is therefore usually not considered.

Finally, vaccination is another option [553]. No vaccine is currently available for horses in Germany. Although a vaccine approved for dogs now covers, among others, the two serovars most commonly found in horses in Germany (Grippotyphosa and Bratislava [18,19,20]), the redesignation of the vaccine for use in horses is risky under pharmaceutical law.

A legal option for the vaccination of horses is the production of a herd-specific vaccine. Vaccine production would be useful if horses on a stud farm show an increased incidence of recurrent uveitis. For this purpose, however, *Leptospira* spp. must first be cultured with sample material from an animal from the farm (e.g., from small rodents or from an equine intraocular sample). If an intraocular sample from a horse is used, the horse should have been on the farm for at least two years before the first ERU bout.

When the culture is positive, it has to be enriched so that the production of a vaccine is possible. Vitreous samples obtained during vitrectomies or chronically infected rodents can be used for the culture. If the owners of the horses from a “problem stud farm” with many horses suffering from ERU take the trouble and costs upon themselves, this approach seems to be promising [19]. However, it is crucial that the vaccination takes place before the systemic infection, i.e., ideally so that antibodies are present in the colostrum and the foals are then vaccinated with the farm-specific leptospiral vaccine analogous to tetanus prophylaxis. If the leptospires are already in the eye, or ERU attacks may even have already occurred, vaccination is no longer useful [554].

In the United States, a vaccine for the serovar Pomona, which is dominant in many regions here, has recently been approved [63]. Whether and how effectively this vaccination prevents disease, whether the single serovar Pomona contained in the vaccine is sufficient, and the level of acceptance for vaccination among horse owners, remains to be seen.

## 10. Discussion

After centuries in which ERU has plagued horse owners and veterinarians, a very significant contribution to clarifying the etiopathogenesis of ERU has been made with the confirmation of the causative chronic intraocular leptospiral infection [18,19] and, most recently, with the demonstration of intraocular biofilm production [57]. ERU fulfils all the characteristics of a biofilm infection, and many of the previously difficult-to-explain characteristics of ERU are now evident:

- Clinically noticeable episodes of uveitis not until many months or even years after systemic infection;
- Cultural leptospiral detection is difficult and has often failed;
- Remarkably, positive cultures, despite high intraocular antibody titers and (although less frequently) antibiotic concentrations in vitreous samples that were well above the MIC for planktonic leptospires;
- Persistence of leptospiral infection in the vitreous cavity for many years;
- Chronic recurrent inflammatory episodes at unpredictable intervals;
- “End-stage ERU” or phthisis: when no vitreous cavity is left, the immunological niche and the infection are also eliminated, and no bacteria are detectable anymore [307,316];
- Outstanding success in terms of postoperative relapse-free outcomes after vitrectomy (mechanical removal of the accessible parts of the vitreous is the best way to eliminate chronic biofilm-associated infections) [480,506,507].

**Figure 2:**
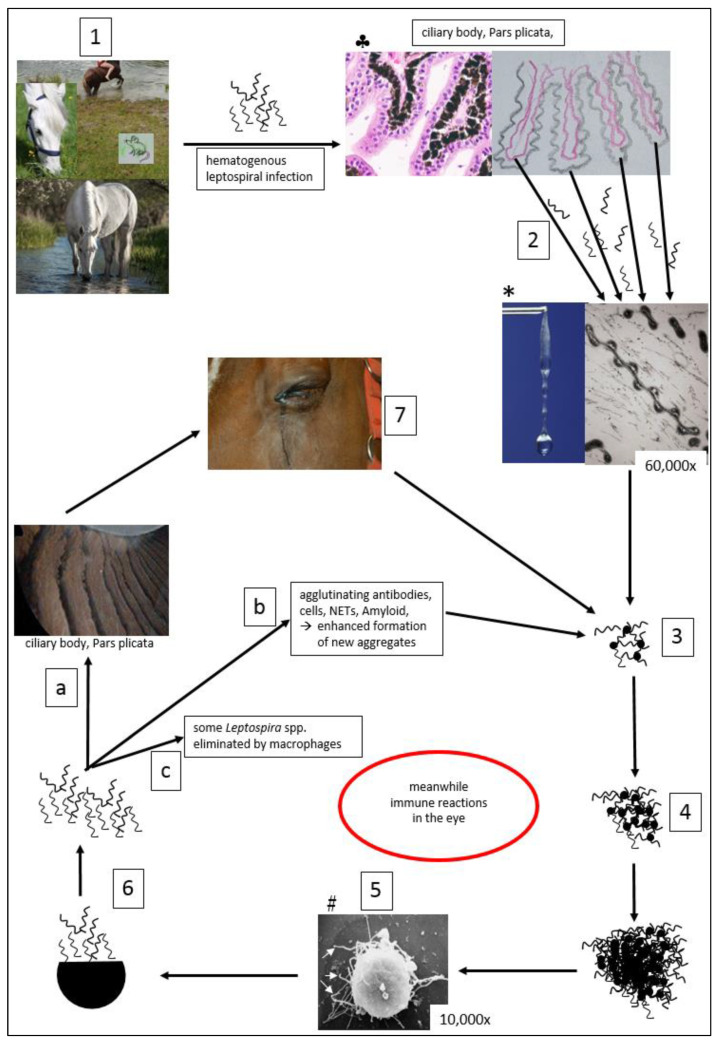
(**1**) Assumed infection route: humid pasture, field mice or other rodents, infection via oral mucous membranes, conjunctiva or skin lesions of the distal limbs; infection usually clinically inapparent [59]. (**2**) Hematogenous transport, possibly influenced by a high burden of *Leptospira* spp. or immune-impaired horse: *Leptospira* spp. leave the fenestrated capillaries of the ciliary body (Pars plicata) and settle in the vitreous chamber. The vitreous is a viscous and low-nutrient medium, and there are collagen fibers within the vitreous body [546]. These are ideal conditions for biofilm formation [544]. ♣ Ciliary body, Pars plicata, healthy equine eye (source: photographs courtesy of Lisa Madlener). * Left: Vitreous material from a healthy equine eye, aspirated with a syringe (photograph: H.G.). Right: *Leptospira* spp. from culture (WHO Grippotyphosa standard strain) within vitreous fibers, transmission electron microscopy (source: photograph courtesy of Gabriele Niedermaier). (**3**) *Leptospira* spp. adhering at vitreous collagenous fibers and sticking together, which immediately induces the production of the extracellular matrix and represents the first step of biofilm formation [485]. Often, “membrane blebbing” or outer membrane vesicles (OMVs) are visible [533]. (**4**) Biofilm formation step 2 [485]: microcolony formation, increasing the extracellular matrix. (**5**) Biofilm formation step 3 [485]: the development of dense round structures, which might be mature biofilm complexes (arrows: possibly hooked ends of *Leptospira* spp.). It is unknown how long the *Leptospira* spp. might stay inside these biofilm constructs, but certainly for several months, and possibly for several years, which can be concluded from experimental and environmental investigations [518]. # Suspected *Leptospira* biofilm in the vitreous samples from an ERU eye, scanning electron microscopy (source: printed image: courtesy of H. Schoel; photograph: B.W.). (**6**) When an unknown signal appears, several biofilm structures probably release planktonic bacteria at the same time. The time-gap until the release is unknown and the signals are unknown. The coordination that several planktonic bacteria emerge from the biofilm at the same time is probably due to quorum sensing (cell-to-cell communication) via channels within the biofilm [466]. The released planktonic *Leptospira* spp. may split and progress in different ways: Immune reactions during the “quiet” stage after infection seem to be sufficiently suppressed by the intraocular mechanisms whose aim is to protect the eye from any damage resulting from inflammations (ocular immune privilege [548]); however, contact of a certain amount of *Leptospira* spp. with the uvea (**a**) might cause a uveitis bout. Meanwhile, there are agglutinating antibodies and antigen-presenting cells within the vitreous body. Consequently, some—but not all—*Leptospira* spp. might be eliminated (**c**). The agglutinating antibodies inside the vitreous cavity might enhance the contact of *Leptospira* spp. with each other and, thus, enhance new biofilm formation (**b**). *Leptospira* spp. show a fascinating motility in viscous media [555] and might spread within the vitreous cavity to form biofilms in new places. Endoscopic picture, pars plicata of the ciliary body (photograph: B.W.). (**7**) Acute uveitis attack: blepharospasm, lacrimation, and swollen eyelids (unspecific symptoms). Ophthalmologic examination would reveal signs of acute uveitis (Section 4.1a) and Figures S1–S5). Under anti-inflammatory therapy, there will be a regression of uveitis. Planktonic bacteria might have been eliminated, the ocular immune privilege is restored, and some bacteria start new biofilm formation.

Although leptospires occasionally enter the anterior chamber of the eye, the vitreous, with its gel-like consistency and collagen fibrils, has emerged as the seat of chronic infection and biofilm formation. In morphological examinations of vitreous samples, the ultrastructural formation of a biofilm is recognizable [424,425,426], and stages of biofilm formation in vitreous samples could be demonstrated immunohistologically (single leptospires, microcolonies, larger dense roundish aggregates) [57]. During vitrectomy, only vitreous material is removed, resulting in permanent elimination of the infection [16]. Both the cultural detection of leptospires and detection of the *LipL32* gene by PCR are somewhat more reliable with vitreous samples compared with aqueous humor samples (Section 7.6). A proposed etiopathogenesis of ERU is shown in Figure 2.

To date, however, there is limited or no knowledge of the morphology and matrix components in biofilms of leptospires formed in vivo. The in vivo composition of biofilms is unquestionably different from that of environmental or in vitro biofilms. In the vitreous, collagen fibrils play a role, as do host immune responses over time. It is likely that the composition of the leptospiral biofilm matrix is critically influenced by the host immune system. Among others, inflammatory products, macrophages, lymphocytes, PMNs, plasma cells, fibrin, amyloids, and NETs have been detected in the vitreous of horses suffering from ERU (Section 7.7, Section 7.8, Section 7.9 and Section 7.10), all of which are in contact with the biofilm in vivo and some of which may also be integrated into it. How quickly biofilm forms in the eye and how old biofilm formations in the vitreous can become is still unknown.

An important point in intraocular leptospiral infection is also the presence of agglutinating antibodies in the aqueous and vitreous humor (Section 7.2 and Section 7.3). In addition to the opsonization of motile leptospires, the aggregation or “agglutination” of leptospires will occur in vivo, just as in MAT. In cases of insufficient phagocytosis, the agglutinating antibodies lead to a progressive attachment of the leptospires to each other (the first step of biofilm formation). The antibodies in the vitreous may thus promote, and perhaps even accelerate, biofilm formation. More rarely, MAT results are negative in intraocular samples; however, instead, either anti-*Leptospira* antibodies can be detected by ELISA and/or PCR can be used to detect the *LipL32* gene. The immune response of the host, which exhibits individual differences, could therefore be of crucial importance for the course of the disease. Here, a genetic predisposition could have an influence (Section 1 and Section 3).

The most common infectious uveitis in humans is caused by *Toxoplasma* spp. [399], but Leptospiral uveitis in humans has more in common with ERU. In humans, leptospiral uveitis is also a non-granulomatous uveitis, often occurring months or years after systemic leptospirosis [220], and is relatively easy to diagnose on the basis of the clinical examination, including the characteristic vitreous opacities [327]. In cases of doubt, an aqueous humor analysis is advised. In warm, humid areas and after flooding, leptospiral etiology is thought to account for about 10% of human uveitis cases. Unlike in horses, leptospiral uveitis in humans often has a milder course, and after a few episodes, is self-limiting. In addition, the systemic administration of antibiotics is reported to be therapeutically effective. This may be because the human vitreous volume is only about one-seventh the size of the equine vitreous in volume. To the best of the authors’ knowledge, in humans, there is no hint of autoimmune uveitis following leptospiral uveitis.

In exploring biofilm infections in the vitreous, some of the therapeutic methods used in ERU have to be questioned (Section 5.2). Injections of triamcinolone into the vitreous cavity and the placement of cyclosporine implants should be reserved for “non-ERU uveitis”. It remains to be seen whether intravitreal injections of gentamicin will prove effective in the long term, and whether and how frequently toxic retinal damage and other complications will occur. For example, it could be that only transiently planktonic leptospires are killed and the bacteria in the biofilm remain largely unaffected—especially because clearance in the vitreous cavity does not allow the prolonged presence of gentamicin. Other prolonged anti-inflammatory therapy options are only temporarily helpful. Elimination of the infection could succeed—if at all—only by the gentamicin injection. However, none of these therapies result in the removal of vitreous opacities; thus, the prognosis for preserving or even improving vision is worse than with a carefully performed vitrectomy.

## 11. Conclusions and Perspectives

Chronic leptospiral infection in the vitreous cavity is only possible through the formation of biofilm. Biofilm formation explains many points in the pathogenesis of ERU that have previously been mysterious. Future ex vivo studies of vitrectomy specimens from ERU eyes could provide insights regarding the composition of the biofilm, the interactions of the different fibrils and fibers (physiological vitreous fibrils, fibrin, NETs, amyloid) with the intraocular biofilm and the immune system, and an improvement in the therapy of biofilm-associated infections. It is possible that aspects of the equine in vivo leptospiral biofilm may be transferable to infections with other spirochaetes and could help to better treat chronic Lyme disease, for example, in humans. ERU occurs spontaneously, is a consequence of a natural leptospiral infection, and vitreous samples are a waste product of the best possible therapy (vitrectomy); therefore, further ex vivo investigations are possible without the need for animal experiments.

## Data Availability

Not applicable.

## Supplementary Materials

The following are available online at https://www.mdpi.com/article/10.3390/microorganisms10020387/s1, Supplementary Material S1, Figure S1: Acute uveitis: Corneal haziness in the periphery, beginning corneal vascularization (visible in the dorsal and temporal aspects), miotic pupil. The accompanying conjunctivitis (hyperemia) seems to be less severe after anesthetic eye drops which cause vasoconstriction. Figure S2: Acute uveitis and similar findings similar to Figure 1, but the circular corneal vascularization is more severe. Figure S3: Left: Small amount of fibrin in the anterior chamber, early stage and mild ERU bout. Right: Severe ERU, hypopyon and corneal vascularization. Figure S4: Severe ERU, the same eye is in both pictures. Left: Corneal haziness in the periphery, corneal vascularization, sero-hemorrhagic inflammation, miotic pupil. Right: After one week with rigorous conservative therapy. The corneal opacity and the inflammatory products in the anterior chamber are decreasing, the pupil is dilated about 2/3, and the fundus reflex indicates substantial vitreous haziness. Figure S5: Left: Very small amount of fibrin (arrow) in the anterior chamber which might be missed if the anterior chamber is not examined carefully. It is not possible to know if this was a mild ERU bout or a blunt trauma. Right: Acute ERU bout after a few days of conservative therapy. The amount of fibrin (arrow) decreases, the pupil is more dilated after the frequent administration of atropine ointments, and the fundus reflex indicates a diffuse vitreous haziness. Figure S6: Increasing vitreous haziness in ERU. a: Normal fundus reflex, b–f: Increasing vitreous haziness, f: The orange-red color indicates a high risk for retinal detachment. Figure S7: Normal view of the optic nerve disc with centrifugal vessels of the equine paurangiotic fundus, b–f: Increasing vitreous haziness, it becomes more and more difficult to see details or even the outline the optic nerve disc. Figure S8: Chronic ERU. Left: Subacute uveitis, rubeosis iridis and neovascularization, posterior synechia, cataract formation. Right: Quiet interval, posterior synechia and cataract formation. Figure S9: Left: Atrophy of the globe, posterior synechiae, cataract, and a “third corner” of the eyelids (arrow) as a sequela of the atrophy. Right: Phthtisis after ERU (“end-stage”) with chronic ocular discharge. Figure S10: Chronic ERU. Left: Subacute uveitis, posterior synechia, severe vitreous haziness. Right: Quiet interval, extensive posterior synechia, beginning cataract formation. Figure S11: Chronic ERU. Left: Posterior synechia and cataract formation. Right: posterior and anterior synechia and cataract formation. Anterior synechia lead to the corneal opacities. Figure S12: Chronic ERU and vesicular cataracts subcapsular of the posterior lens capsule. Left: Vesicular cataract in the nasal aspect. Temporally small posterior synechia. Middle: Vesicular cataract (very small “bubbles”), especially in the ventral aspect of the lens. At the “7 o’clock” position and at the “11 o’clock” position are small posterior synechia. In the dorsal aspect are inflammatory products on the posterior lens capsule. Right: Vesicular cataract in the periphery (relatively large or possibly confluent “bubbles”). At the “1 o’clock” position small vitreous floaters. Figure S13: Chronic ERU, quiet interval. Arrowhead: Iris residue on the anterior lens capsule. Arrow: Dense inflammatory products (“floaters”) in the vitreous cavity, very close to the posterior lens capsule. These inflammatory products move (“float”) in the vitreous after blinking and can best be seen and assessed using a hand-held ophthalmoscope. Figure S14: Chronic ERU. Star-shaped retinal folds (arrows) around the optic nerve disc. This degree of retinal detachment means an increased risk for retinal detachment. If vitrectomy is successfully performed and there is no retinal detachment perioperatively, vision might be preserved. Figure S15: Chronic ERU. Left: Large-scale detachment of the retina. This kind of detachment will progress, leading to blindness. Right: Complete retinal detachment. The retina is still fixed around the optic nerve disc, but no longer at the dorsal and lateral aspects of the Ora serrata. Figure S16: Leopard coat pattern uveitis: cataract and posterior lens luxation in the left eye. Figure S17: Uveitis: blood and fibrin in the anterior chamber. These findings may be due to a blunt trauma. Figure S18: Phacogenic uveitis: the protrusion of lens material (arrow) through a circular defect in the anterior lens capsule. In the ventral aspect of the lesion iris, pigment is left after posterior synechia. Figure S19: Chronic iritis, similar to “Fuchs’ heterochromic iritis” in humans: depigmentation in the iris and corneal edema. Figure S20: Uveitis (both eyes affected) accompanying septicemia (*Rhodococcus equi*). In foals younger than 6 months, ERU is extremely unlikely. Figure S21: Medulloepithelioma causing mild and insidious uveitis. Figure S22: Ongoing painful uveitis despite rigorous conservative therapy in a horse with systemic *Micronema deletrix* (syn: *Halicephalobus deletrix*) infection. The nematodes were later histologically detected in the uveal tissue. Figure S23: Uveitis accompanying severe keratitis. The focus must be on the corneal infection. Once the infection is removed, the uveitis will not continue. Figure S24: Septic endophthalmitis: corneal vascularization is much denser than vascularization accompanying ERU. Furthermore, the purulent infection of the inner eye leads to more intense corneal edema as well as another type of cloudiness of the normally transparent media. Furthermore, the horses exhibit fever and a significant disturbance of their general condition. Supplementary Material S2: Notes on the side effects of topically administered atropine in horses. Supplementary Material S3: Notes on the intravitreal injection of gentamicin. Supplementary Material S4, Table S1: Follow-up examination of aqueous samples after vitrectomy: course of anti-*Leptospira* antibody titers (MAT) (unpublished data). Figure S25: MAT titers over time after vitrectomy. In individual horses, an aqueous humor sample could be taken at different times after surgery (e.g., when a fibrinolytic was injected after surgery or after euthanasia due to other underlying diseases). Each arrow represents one eye. The arrows start at the time of surgery and the arrowhead indicates follow-up aqueous humor testing. Supplementary Material S5, Table S2: Literature references for testing intraocular samples from ERU eyes or human uveitis eyes for leptospires (culture, PCR, histology/immunohistochemistry, and electron microscopy). Supplementary Material S6: Results of examinations of intraocular samples from horses suffering from ERU (excerpts from [18]). Table S3: Protein fractions determined by electrophoresis (total protein “TP” and albumin “Alb.”) and calculated (globulins, “Ig”) from serum (S) and vitreous (V) samples and calculation of the Goldmann–Witmer coefficient (GWC) in 46 paired vitreous and serum samples. Results sorted by GWC (decreasing). Table S4: MAT titers in serum (S) and undiluted vitreous samples (V) from horses with ERU and from horses with healthy eyes. Intraocular MAT titers exceed the serum titers by several times. A clear difference between horses suffering from ERU and horses with healthy eyes is only seen when looking at the intraocular samples. Table S5: MAT results with intraocular and serum samples from horses with ERU (ophthalmological findings differentiated) and from horses with healthy eyes. Figure S26: Typical SNAP Lepto results in an ERU horse. Upper picture: SNAP Lepto with undiluted vitreous from an ERU eye. Picture below: SNAP Lepto with serum of the same horse. The sample point was at the “12 o’clock position” and the control point was at the “9 o’clock position”. The point of the vitreous sample is even darker than the control point, indicating a high level of intraocular antibodies. The point of the serum sample shows only a very weak coloring, which is barely visible. (SNAP Lepto is a commercially available rapid ELISA test for the detection of anti-*LipL32* antibodies from IDEXX company, Ludwigsburg, Germany) (photograph: B.W.). Figure S27: Percentages of positive vitreous and serum samples from ERU eyes and ERU horses, respectively, using MAT (titer ≥ 1:100). Figure S28: Positive leptospiral culture results (*n* = 189) with vitreous samples compared with MAT results in serum samples. Percentage of assignment to serogroups. Sometimes MAT was positive (titer ≥ 1:100) for more than one serovar. Figure S29: Positive leptospiral culture results (*n* = 189) with vitreous samples compared with MAT results in the same samples. Percentage of assignment to serogroups. Sometimes MAT was positive (titer ≥ 1:100) for more than one serovar. Supplementary Material S7: Literature references cited in publications on the examination of intraocular specimens from eyes affected with ERU for the calculation of GWC or “C-values”. Figure S30: References for the calculation and interpretation of the Goldmann–Witmer coefficient (GWC) in the literature. Arrows mean “cites”. Red references: calculation of the “C-value” without any additional testing, e.g., for the IgG, albumin, or total protein contents in corresponding intraocular and serum samples. Black references: no calculation of the GWC at all—although cited for the GWC. Blue references: the exact calculation path for correct GWC calculations is not described, but measurements of the IgG-levels in intraocular fluids and serum samples are included in the methods and correct citations for the calculation path are given. Green references: correct calculation paths of GWC are given. Black arrows: citation of references which did not use or describe the GWC at for their work at all. Red arrows: citation of references for calculations of the GWC or “C-value”, respectively, in which the calculation is not described. Dashed arrows: citation of references in which the correct calculation of the GWC is described, but the citing reference ignores the measurement of IgG or another protein fraction anyway. Blue arrows: citation of references in which the methods describe measurements of IgG levels and cite references for the correct calculation paths of the GWC. Green arrows: correct citations for calculation of the GWC. Supplementary Material S8: Ultrastructural and histological examinations of vitreous specimens and the ciliary body. Figure S31: Transmission electron microscopy pictures. A and B: *Leptospira grippotyphosa* (WHO standard strain) were experimentally injected into the vitreous body of a healthy eye of a euthanized horse. Afterwards, the vitreous sample was taken by vitrectomy. A: *Leptospira* spp. seem to arrange themselves along with the vitreous fibers, which would be an excellent starting position for biofilm formation. C and D: Vitreous samples taken during therapeutic vitrectomy from a horse with naturally acquired ERU. *Leptospira* spp. are surrounded by a thick layer of an extracellular osmiophilic matrix which lacks the experimentally injected *Leptospira* spp. (Source: [424], reprint courtesy of Schluetersche Specialized Media GmbH, Hanover, Germany). Figure S32: Vitreous material from an ERU eye prepared for transmission electron microscopy showing phagocytosis of dense round structures. Two smaller structures are inside the phagocyte (numbers 1 and 2); the large one (number 3) probably leads to a “frustrated phagocytosis”. These dense round structures are now suspected to be the leptospiral biofilm. Inside of the phagocyte are also structures which may be parts of *Leptospira* spp. (arrowheads). Bar = 2 µm (photograph courtesy of Kristin Brandes). Figure S33: Ciliary body region of an ERU eye. Congo Red staining and polarized light showing amyloid on the ciliary body. Subsequent immunohistology revealed amyloid A [224]. (Photographs courtesy of Maj-Britt Cielewicz). Figure S34: Dense vitreous opacities (“floaters”). a: In the dorsal aspect of the vitreous chamber, b: Further distributed in the vitreous chamber, c: Membrane-like vitreous opacity mimicking retinal detachment. Bottom: Syringe containing vitreous material (and several “floaters”) obtained in the beginning of vitrectomy. (Photographs: B.W. and H.G.). Figure S35: Amyloid: Vitreous opacities (Supplementary Material S1, Figure S13 and Supplementary Material S8, Figure S34) after Congo Red staining and the presence of green birefringence in polarized light. (Photographs courtesy of Ellen Giving). References [556,557,558,559,560,561,562,563,564,565,566,567,568,569,570,571,572,573,574,575,576] are cited in the Supplementary materials.
