# Supplementary material for: Infectious Uveitis in Horses and New Insights in Its Leptospiral Biofilm-Related Pathogenesis
Source: Microorganisms. 2022 Feb 7;10(2):387. doi: 10.3390/microorganisms10020387 (PMC8875353; doi:10.3390/microorganisms10020387)
Supplement: Supplementary file 1 [file microorganisms-10-00387-s001.zip › microorganisms-1560157-supplementary - proofread.pdf]

microorganisms

MDPI

Supplementary 1

Examples of clinical findings in ERU-eyes. (In acute uveitis, there is often a diffuse corneal opacity, so that the photos sometimes appear slightly blurred.) (photographs: BW and HG).

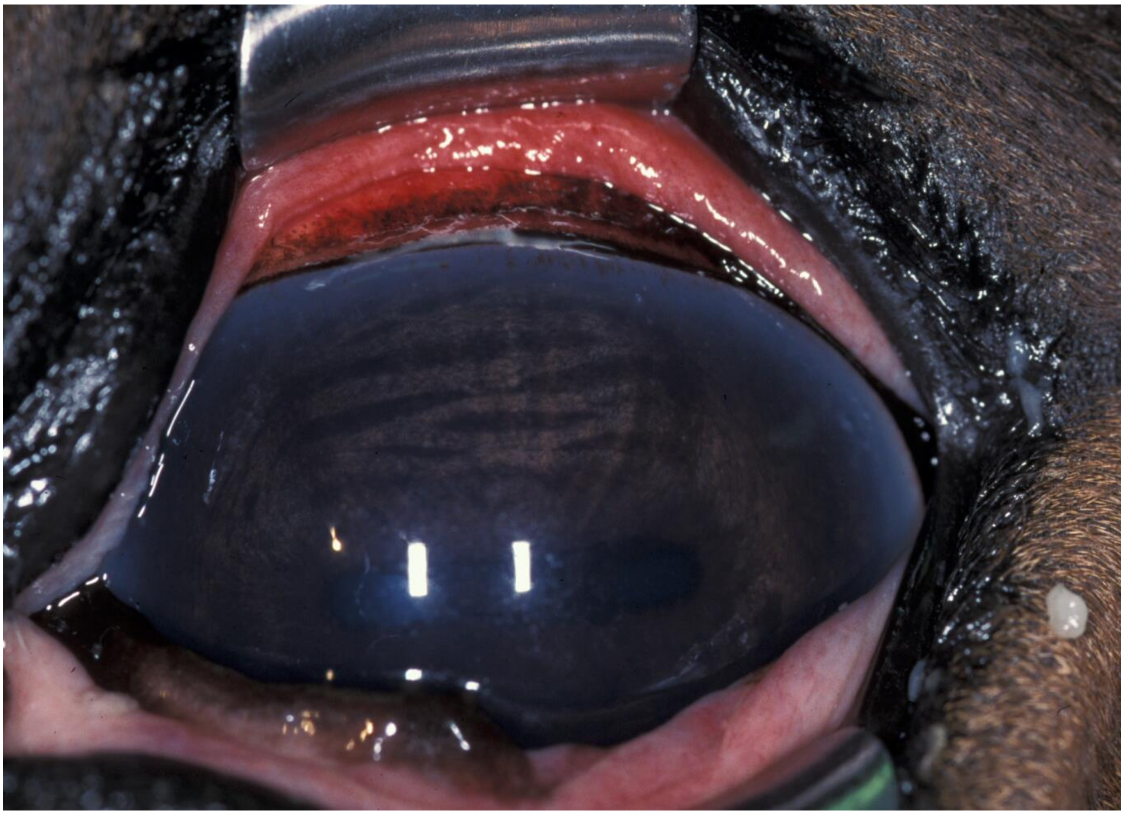

Figure S1. Acute uveitis: Corneal haziness in the periphery, beginning corneal vascularization (visible in the dorsal and temporal aspects), miotic pupil. The accompanying conjunctivitis (hyperemia) seems to be less severe after anesthetic eye drops which cause vasoconstriction.

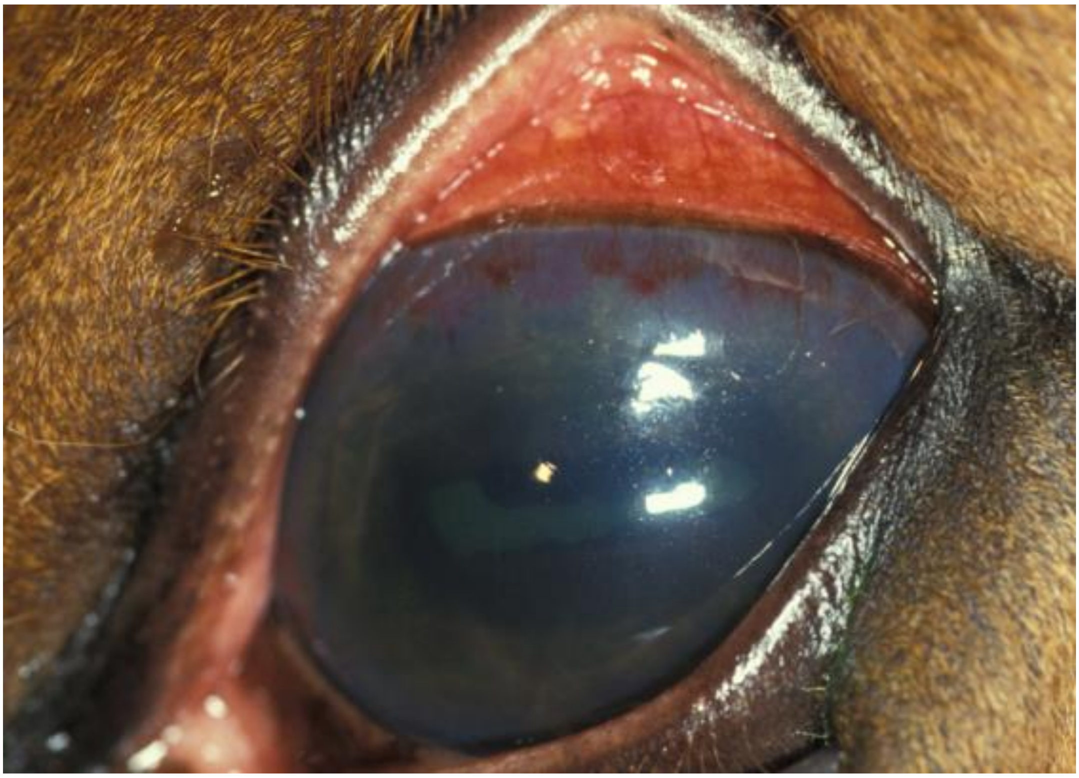

Figure S2. Acute uveitis and similar findings like in figure 1, but the circular corneal vascularization is more severe.

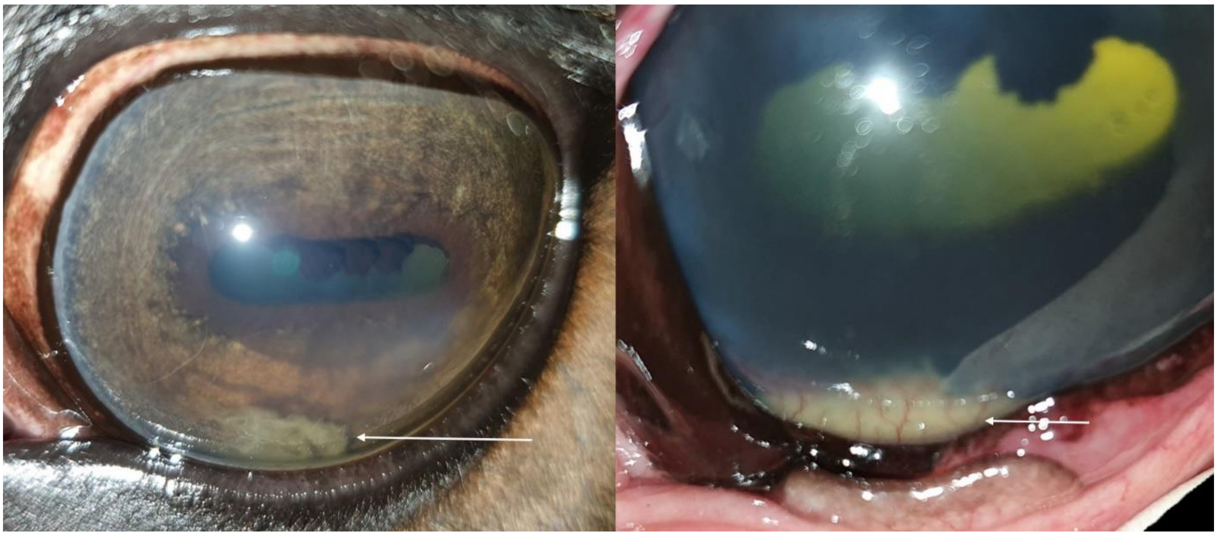

Figure S3. Left: Small amount of fibrin in the anterior chamber, early stage and mild ERU-bout. Right: Severe ERU, hypopyon and corneal vascularization.

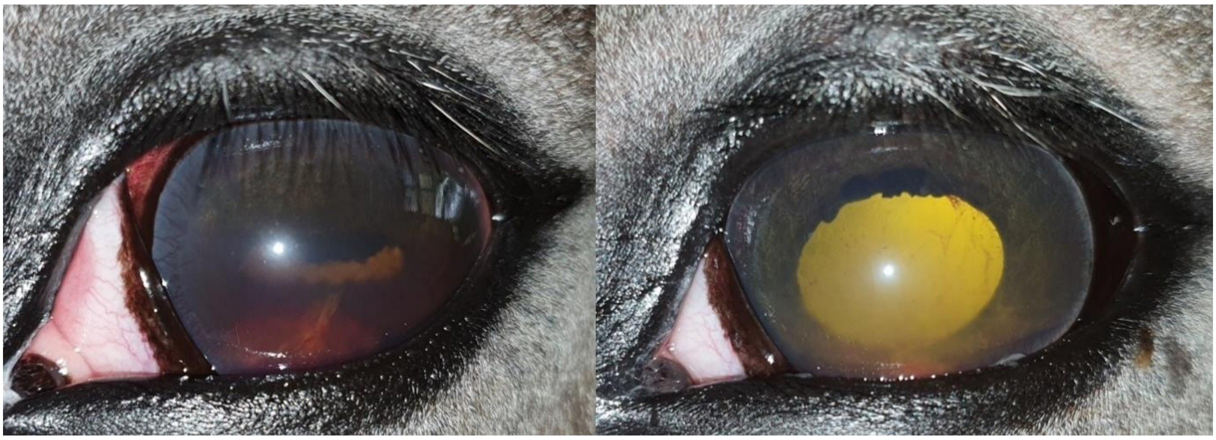

Figure S4. Severe ERU, the same eye is in both pictures. Left: Corneal haziness in the periphery, corneal vascularization, sero-hemorrhagic inflammation, miotic pupil. Right: After one week with rigorous conservative therapy. The corneal opacity and the inflammatory products in the anterior chamber are decreasing, the pupil is dilated about 2/3 and the fundus reflex indicates substantial vitreous haziness.

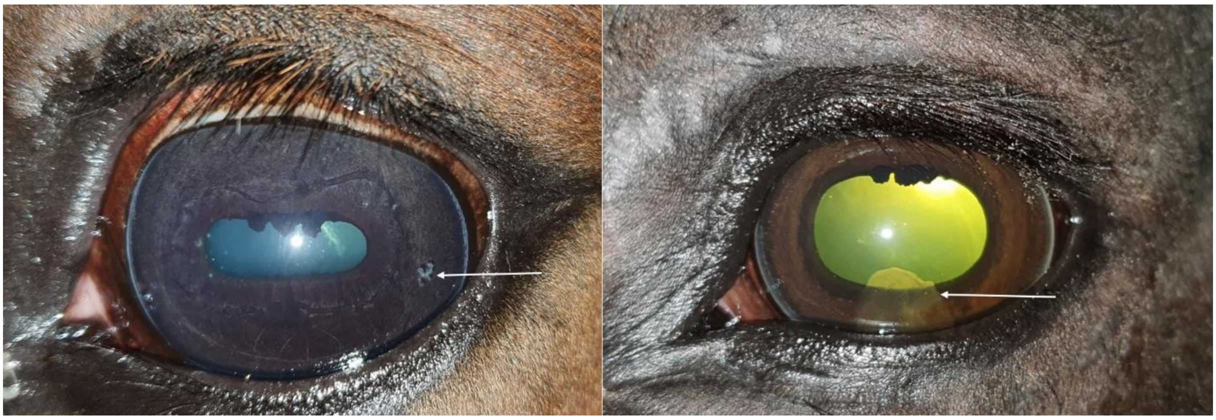

Figure S5. Left: Very small amount of fibrin (arrow) in the anterior chamber which can be seen best when floating but might be missed if the anterior chamber is not examined carefully. It is not possible to know if this was a mild ERU-bout or a blunt trauma. Right: Acute ERU-bout after a few days of conservative Therapy. The amount of fibrin (arrow) decreases, the pupil is more dilated after frequent administration of atropine ointments and the fundus reflex indicates a diffuse vitreous haziness.

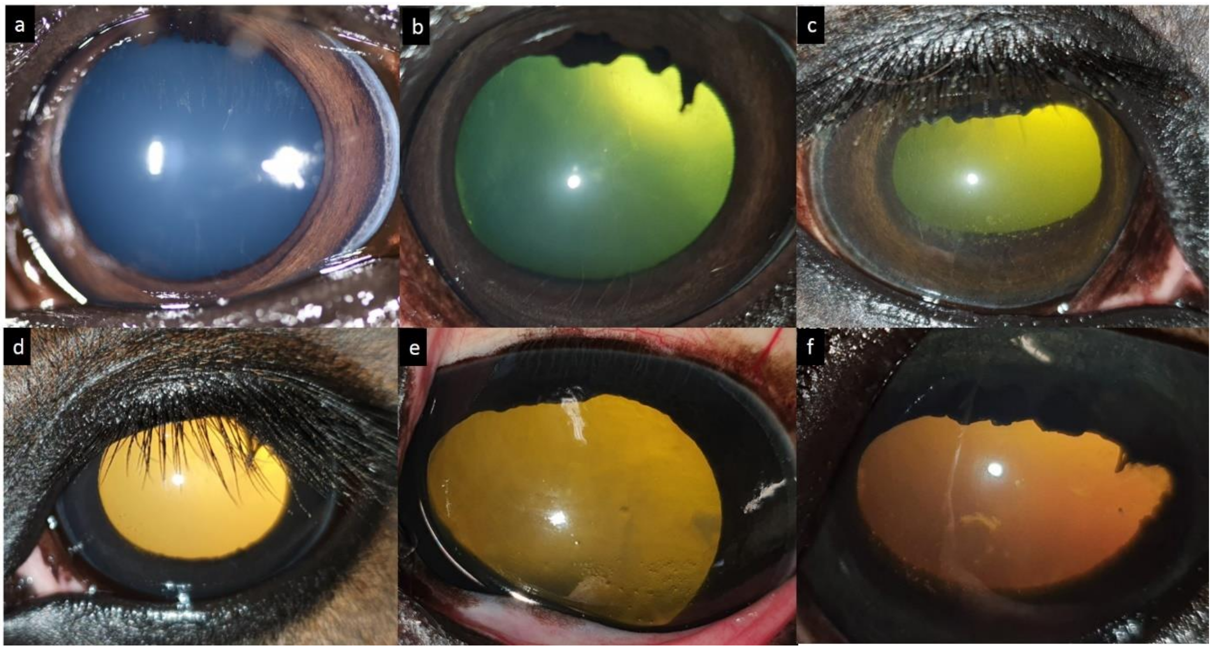

Figure S6. Increasing vitreous haziness in ERU. a: Normal fundus reflex, b – f: Increasing vitreous haziness, f: The orange-red color indicates a high risk for retinal detachment.

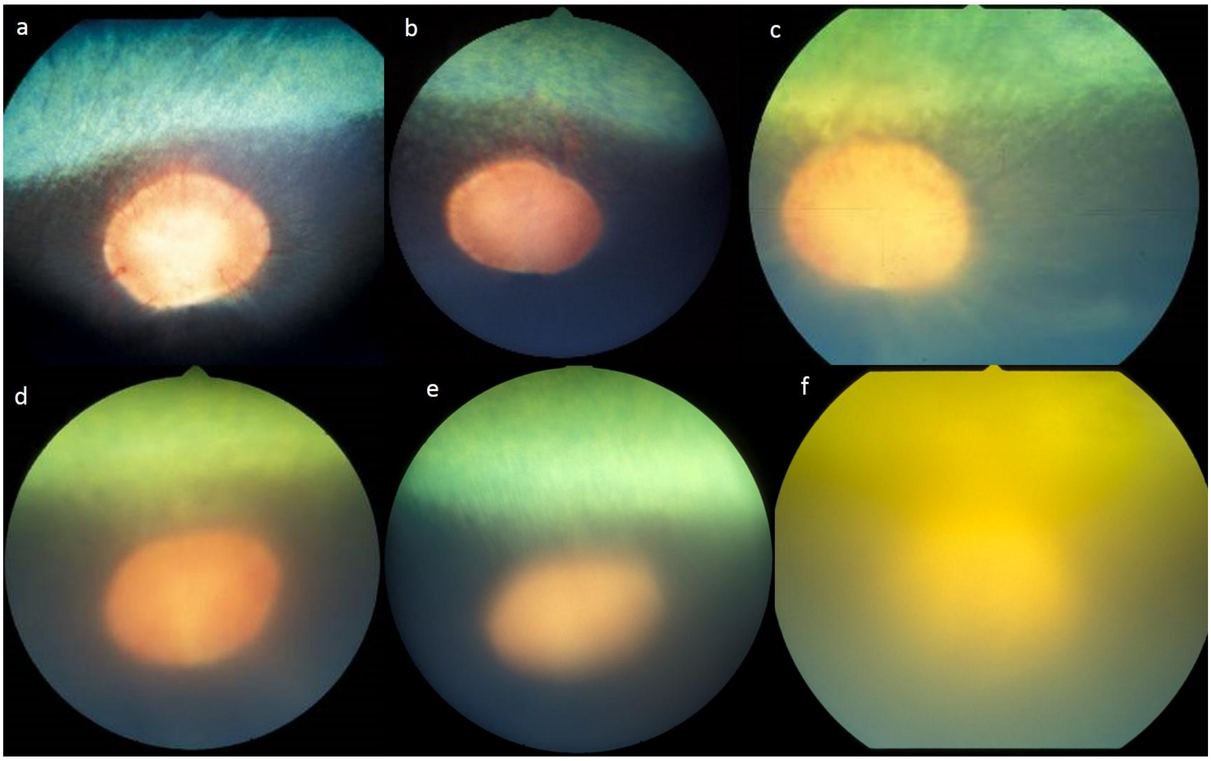

Figure S7. a: Normal view of the optic nerve disc with centrifugal vessels of the equine paurangiotic fundus, b – f: Increasing vitreous haziness, it becomes more and more difficult to see details or even the outline the optic nerve disc.

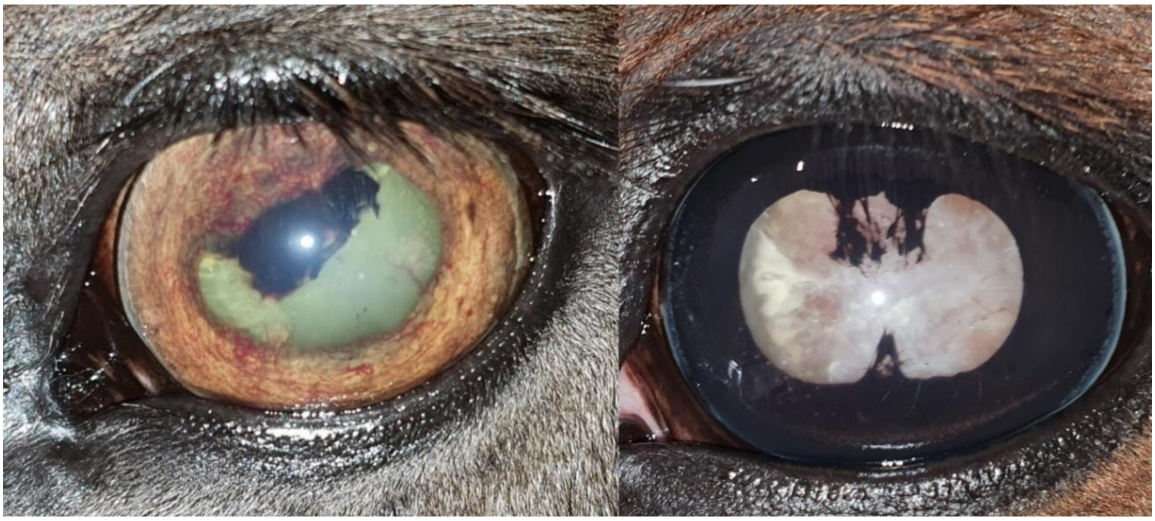

Figure S8. Chronic ERU. Left: Subacute uveitis, rubeosis iridis and neovascularization, posterior synechia, cataract formation. Right: Quiet interval, posterior synechia and cataract formation.

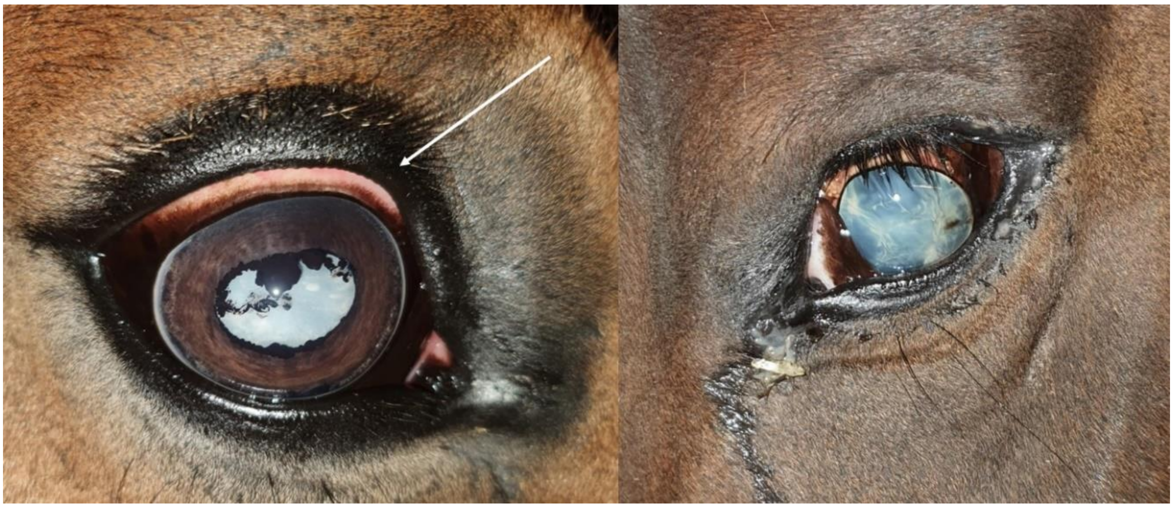

Figure S9. Left: Atrophy of the globe, posterior synechiae, cataract, and a "third corner" of the eyelids (arrow) as a sequela of the atrophy. Right: Phthisis after ERU (= "end-stage") with chronic ocular discharge.

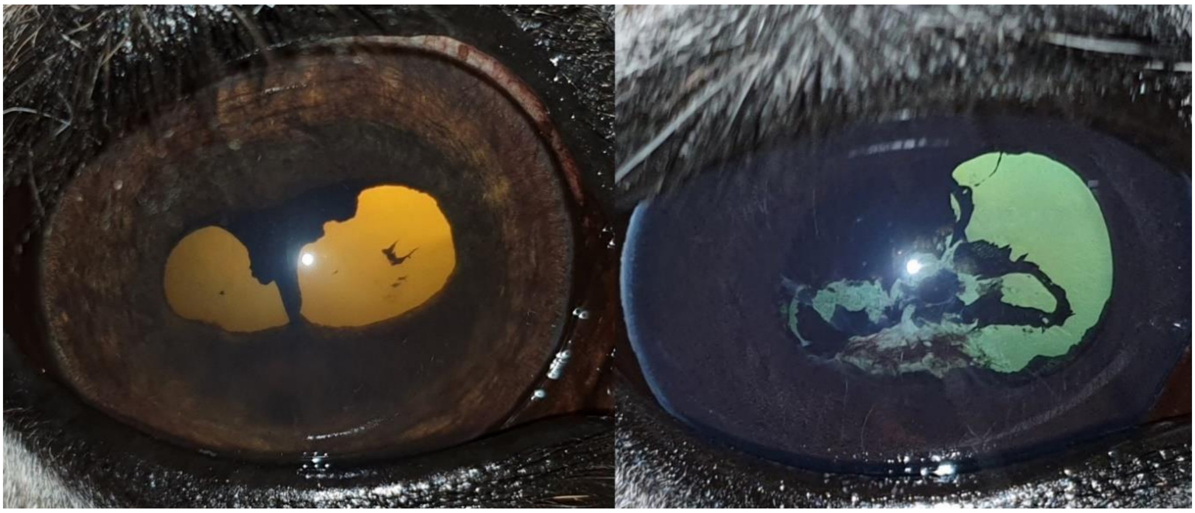

Figure S10. Chronic ERU. Left: Subacute uveitis, posterior synechia, severe vitreous haziness. Right: Quiet interval, extensive posterior synechia, beginning cataract formation.

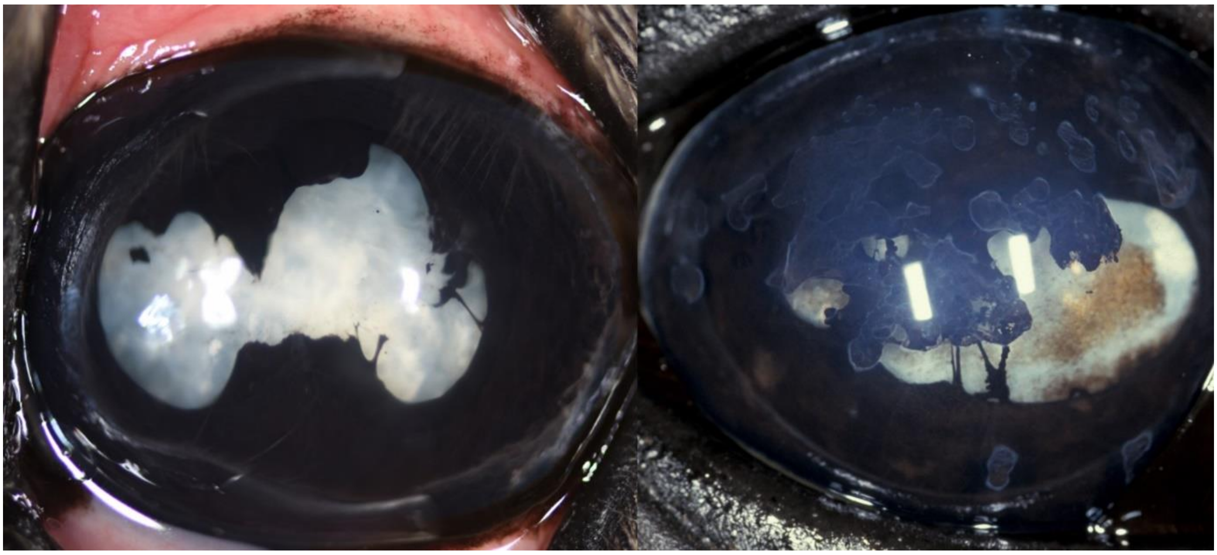

Figure S11. Chronic ERU. Left: Posterior synechia and cataract formation. Right: posterior and anterior synechia and cataract formation. Anterior synechia lead to the corneal opacities.

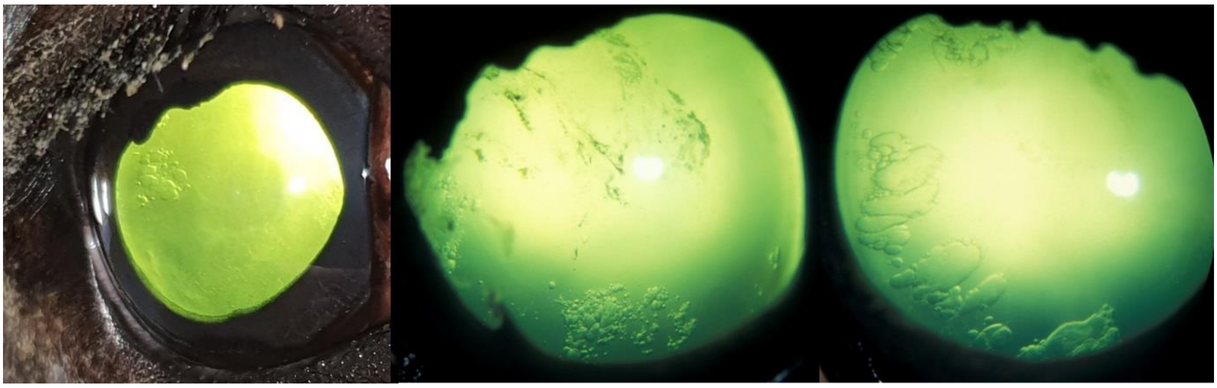

Figure S12. Chronic ERU and vesicular cataracts subcapsular of the posterior lens capsule. Left: Vesicular cataract in the temporal aspect of the lens. Middle: Vesicular cataract (very small "bubbles") especially in the ventral aspect of the lens. At the "7 o'clock"-position and at the "11 o'clock"-position small posterior synechia. In the dorsal aspect inflammatory products on the posterior lens capsule. Right: Vesicular cataract in the periphery (relatively large or possibly confluent "bubbles"). At the "1 o'clock"-position small vitreous floaters close to the lens.

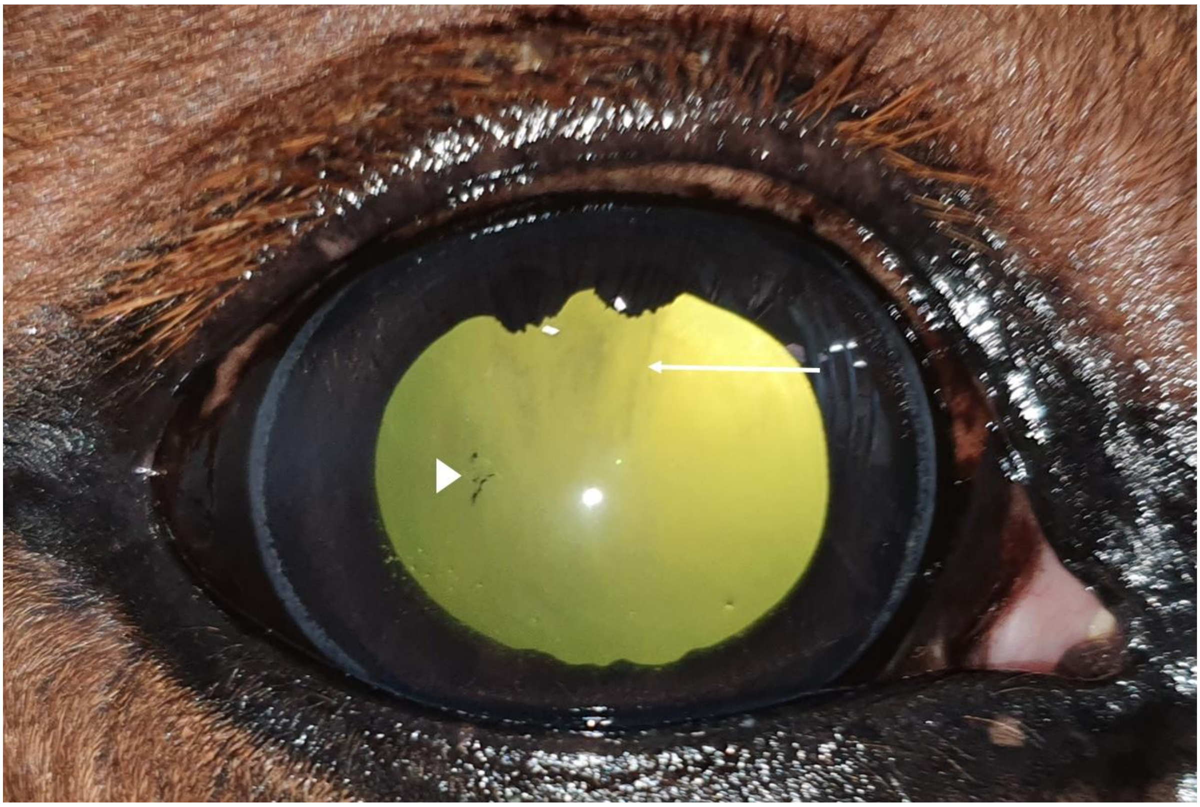

Figure S13. Chronic ERU, quiet interval. Arrowhead: Iris residuae on the anterior lens capsule. Arrow: Dense inflammatory products (“floaters”) in the vitreous cavity, very close to the posterior lens capsule. These inflammatory products move (“float”) in the vitreous after blinking or eye movements and can best be seen and assessed using a hand-held ophthalmoscope.

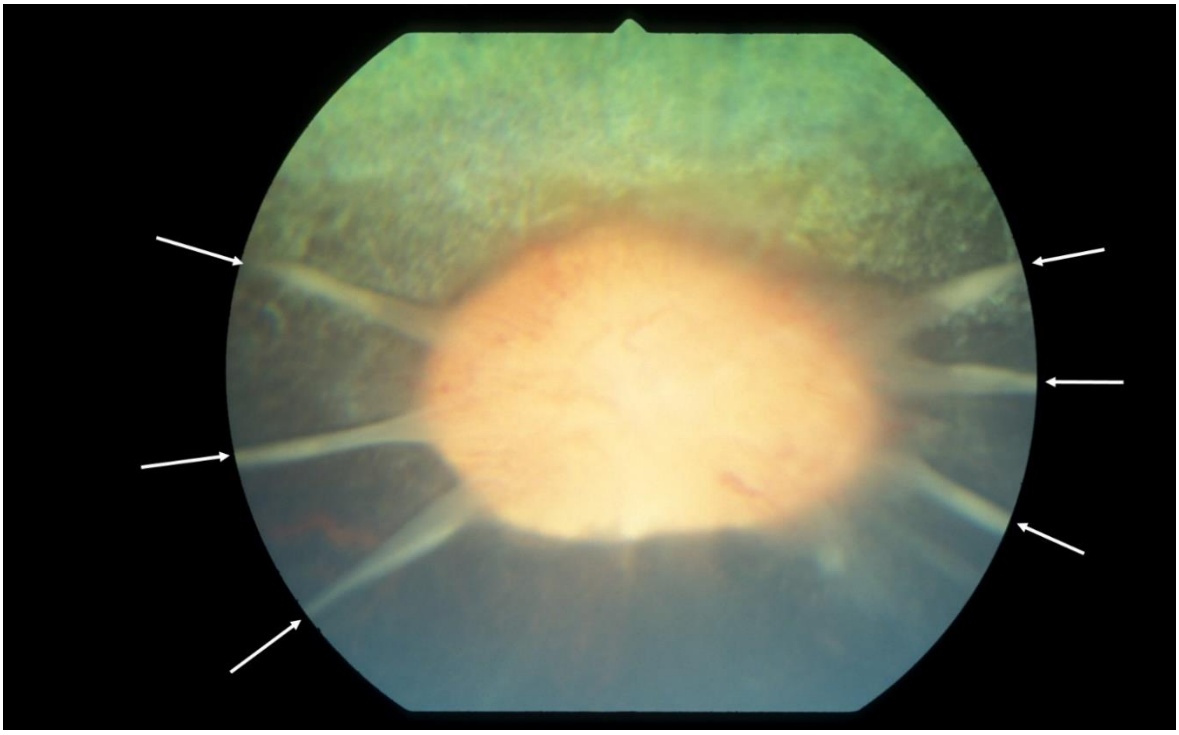

Figure S14. Chronic ERU. Star-shaped retinal folds (arrows) around the optic nerve disc. This degree of retinal detachment means an increased risk for retinal detachment. If vitrectomy is performed very carefully and if there is no retinal detachment intraoperatively, in many times, vision can be preserved. In some patients with such previous findings the retinal folds were less prominent or even gone on reexamination after several months.

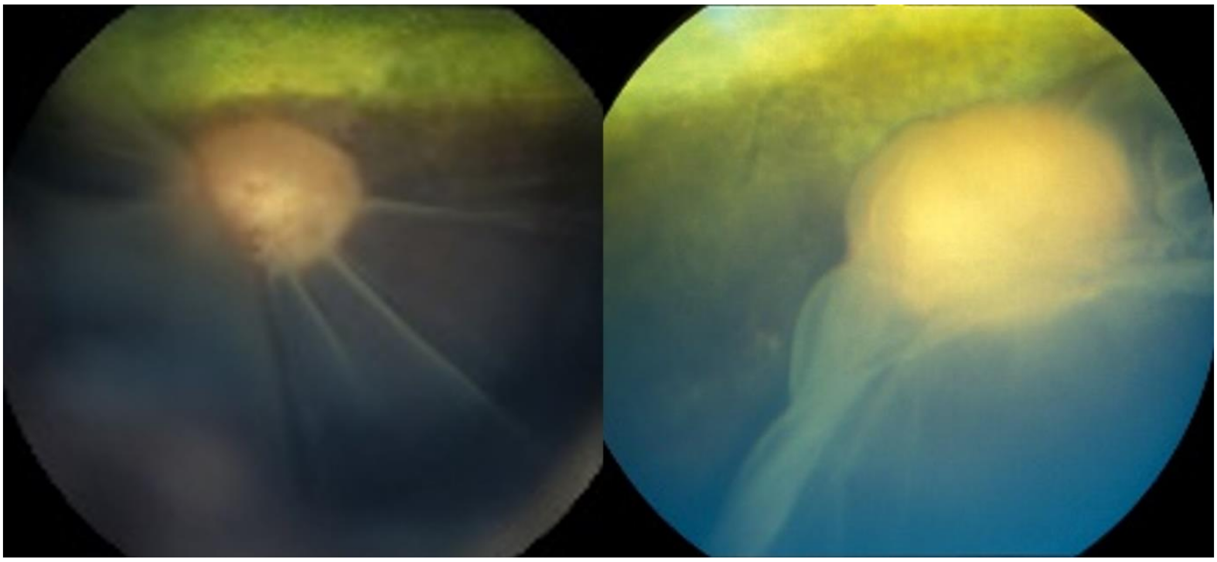

Figure S15. Chronic ERU. Left: Large-scale detachment of the retina. This kind of detachment will progress, leading to blindness. Right: Complete retinal detachment. The retina is still fixed around the optic nerve disc, but no longer at the dorsal and lateral aspects of the Ora serrata.

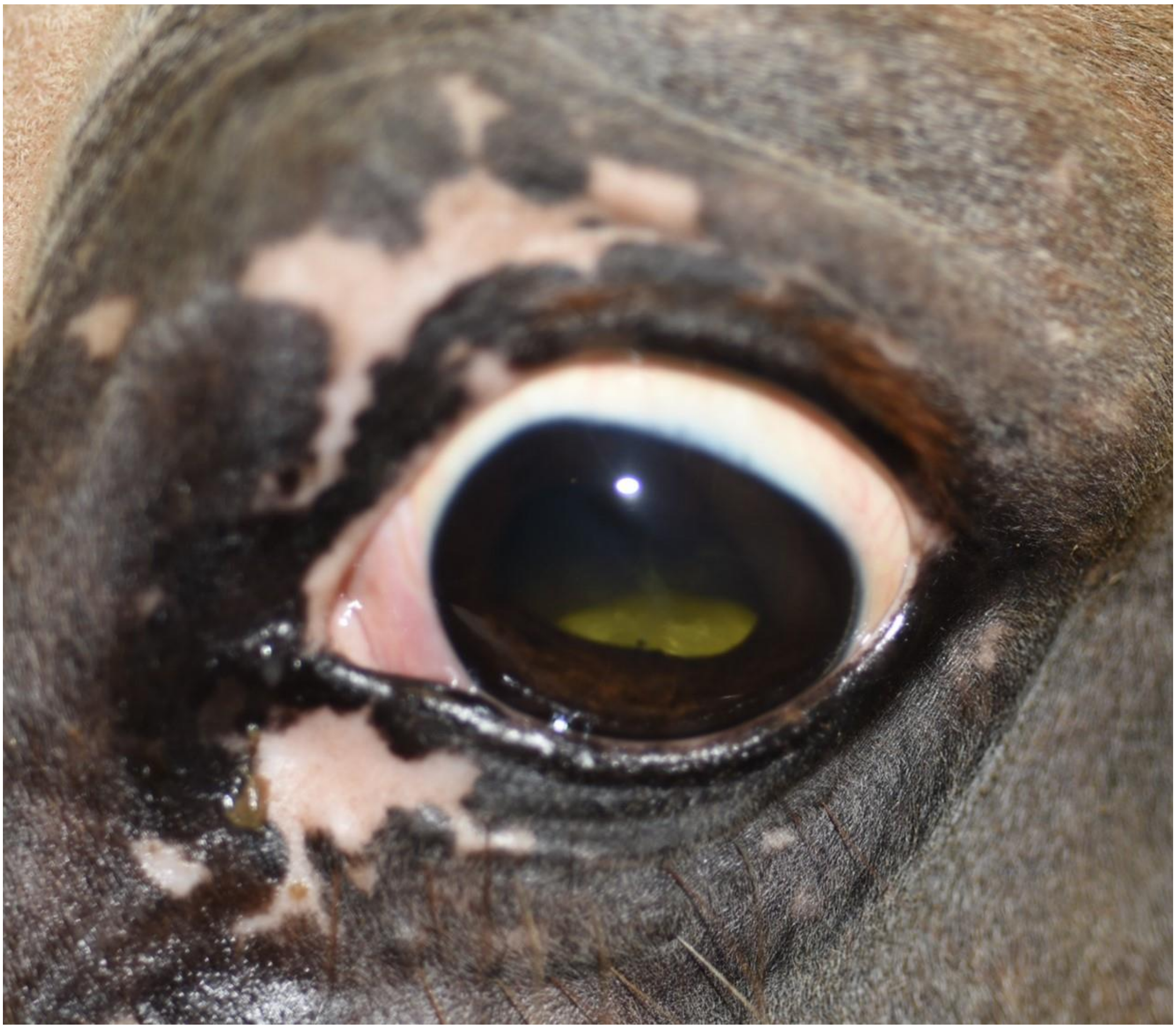

Figure S16. Leopard coat pattern uveitis: Cataract and posterior lens luxation in the left eye.

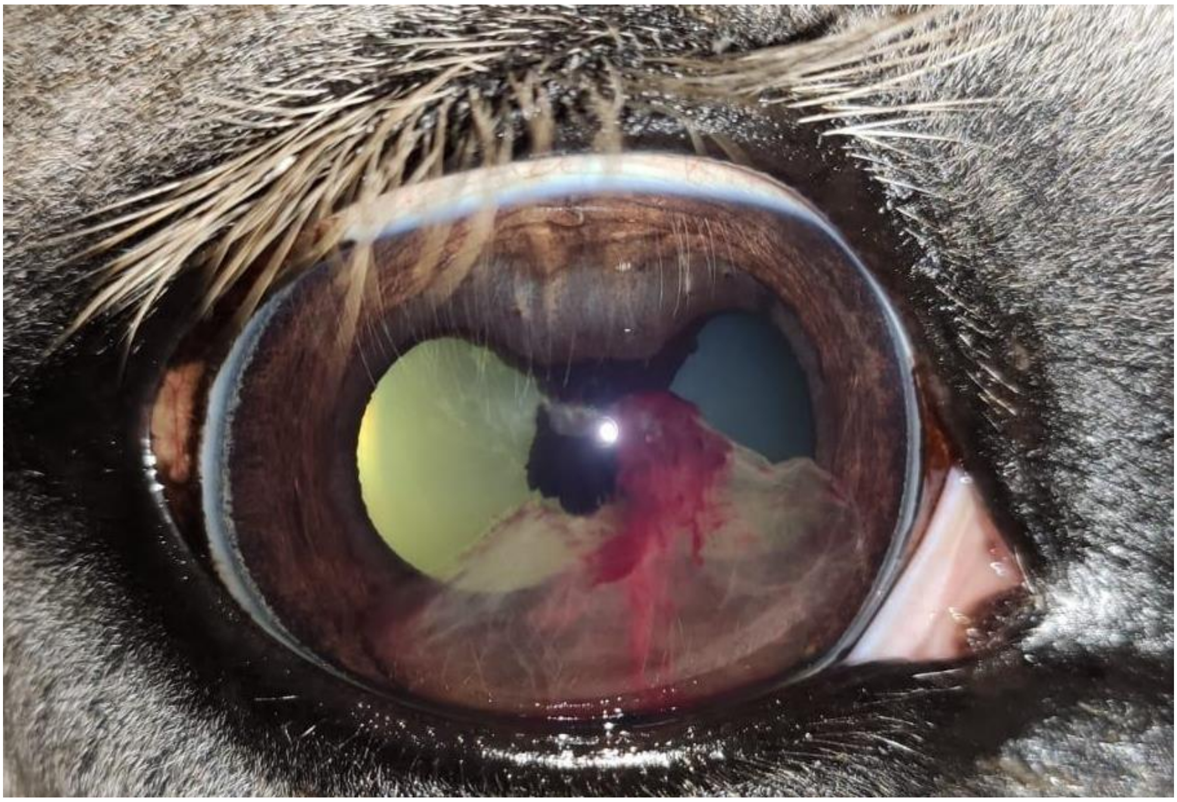

Figure S17. Uveitis: Blood and fibrin in the anterior chamber. These findings can be a result of a blunt trauma.

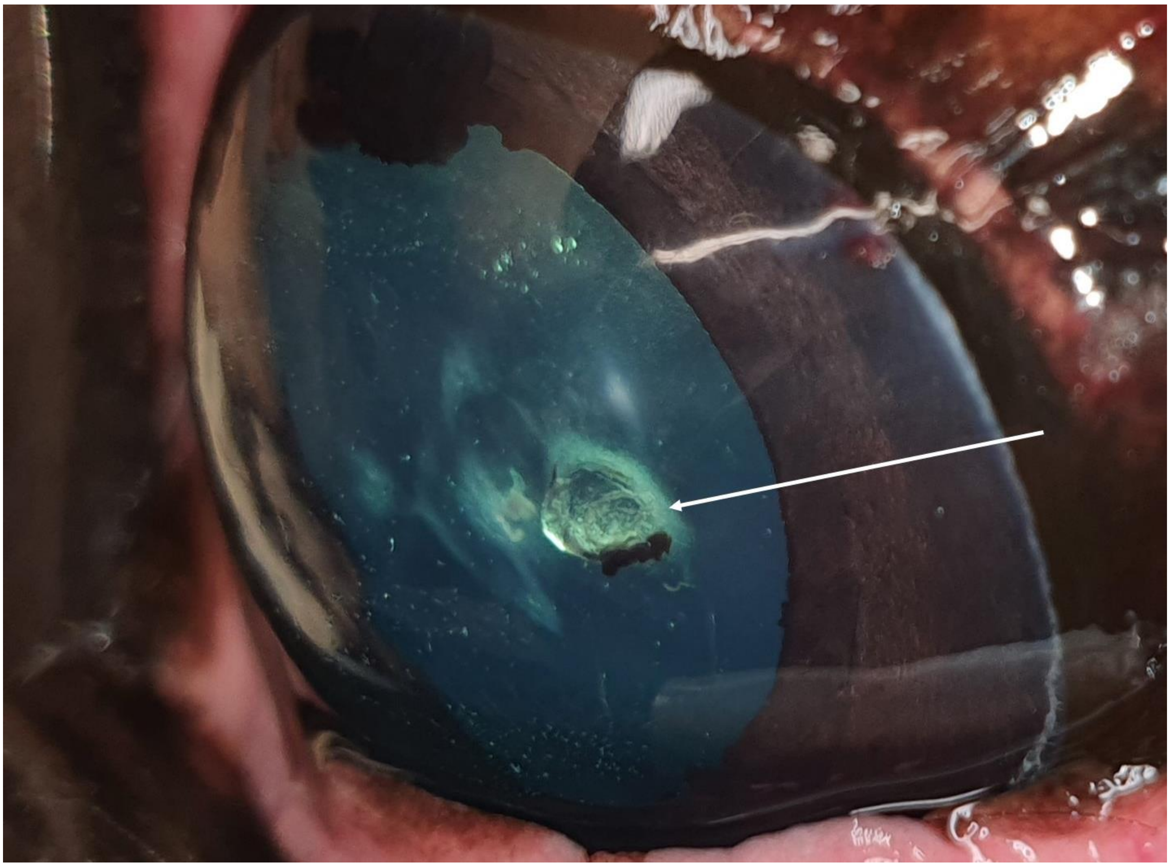

Figure S18. Phacogenic uveitis: protrusion of lens material (arrow) through a circular defect in the anterior lens capsule. In the ventral aspect of the lesion iris pigment is left after posterior synechia.

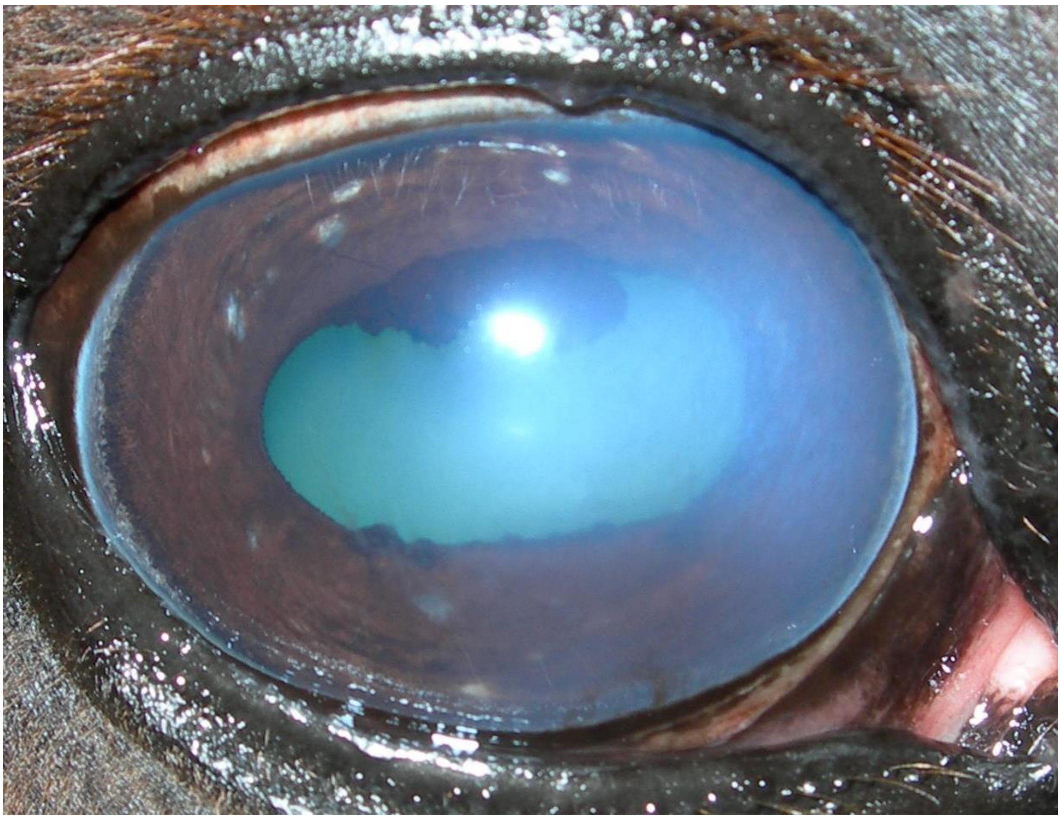

Figure S19. Chronic iritis, similar to “Fuchs’ heterochromic iritis” in humans: Depigmentation in the iris and chronic corneal edema, probably due to endotheliitis.

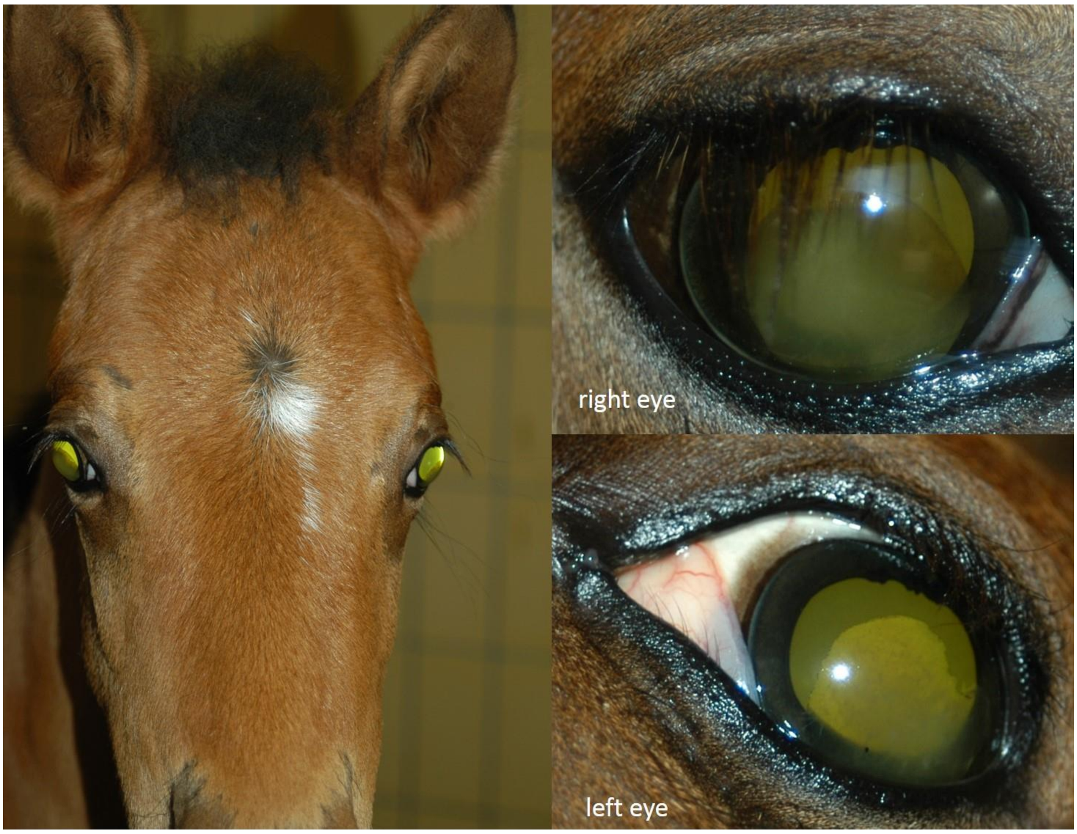

Figure S20. Uveitis (both eyes affected) accompanying septicaemia (*Rhodococcus equi*). In foals younger than 6 months ERU is extremely unlikely.

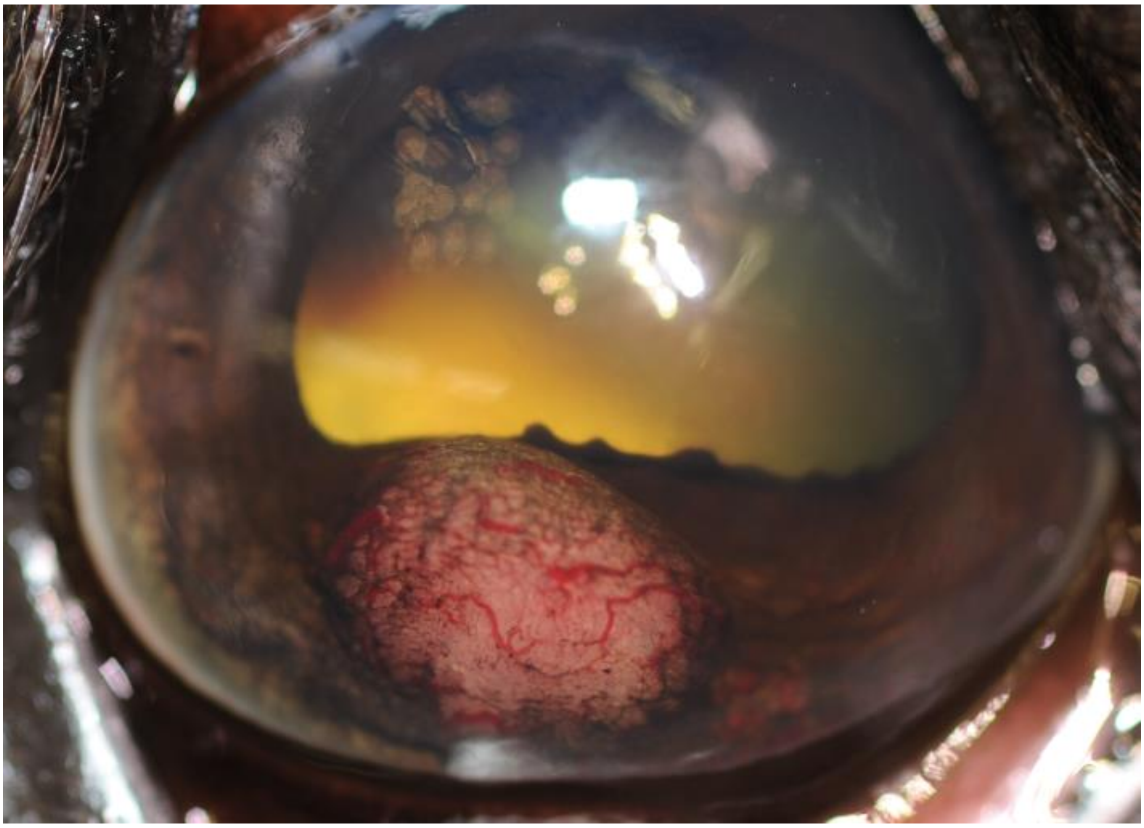

Figure S21. Medulloepithelioma causing mild and insidious uveitis and blindness.

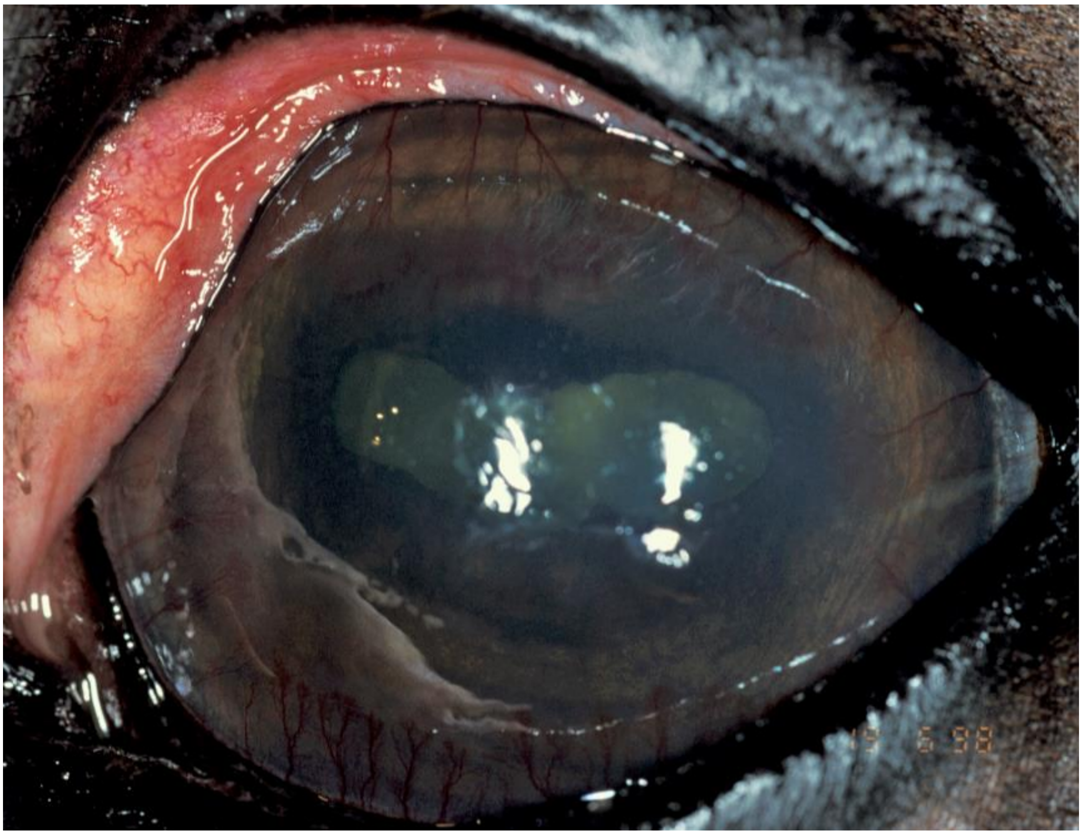

Figure S22. Ongoing painful uveitis despite meticulous conservative therapy in a horse with systemic *Micronema deletrix* (syn: *Halicephalobus deletrix*) infection. The nematodes were later histologically detected in the uveal tissue.

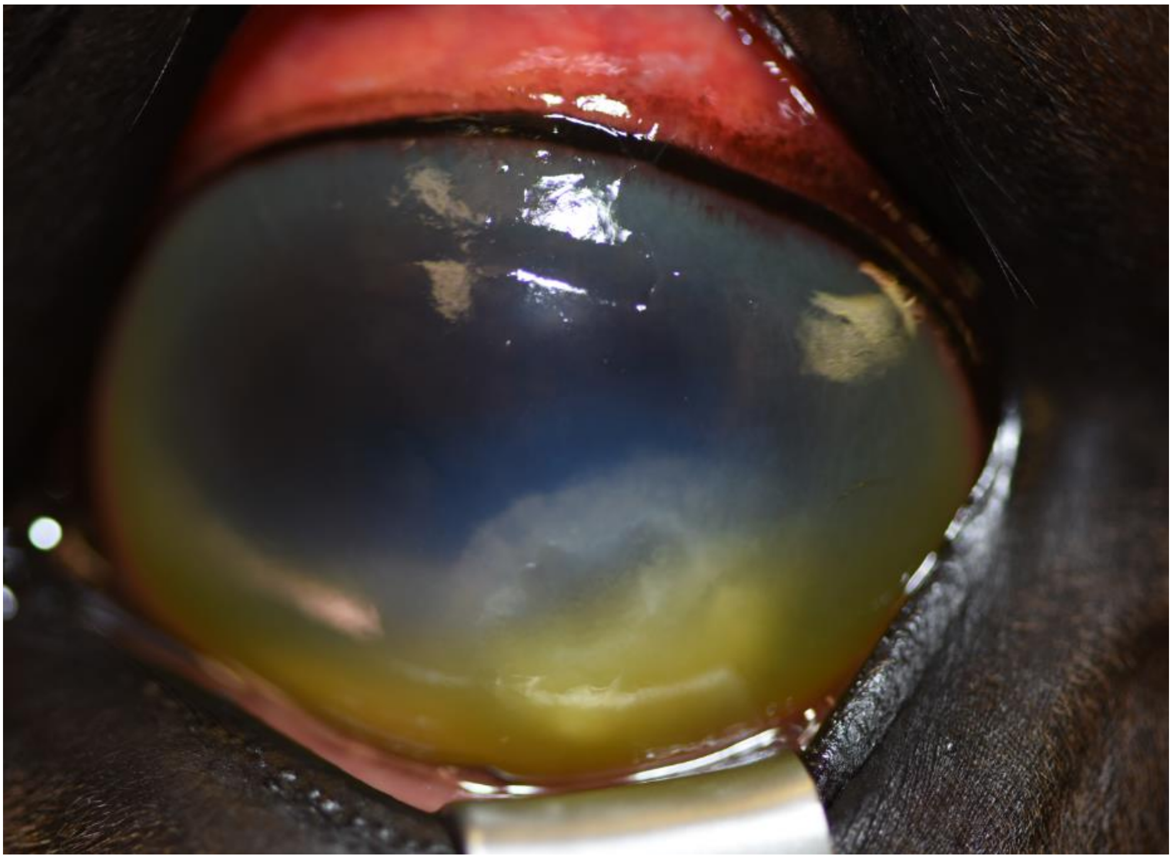

Figure S23. Uveitis accompanying severe keratitis. The main problem is the corneal infection. Once the infection is removed, the uveitis is not going to continue.

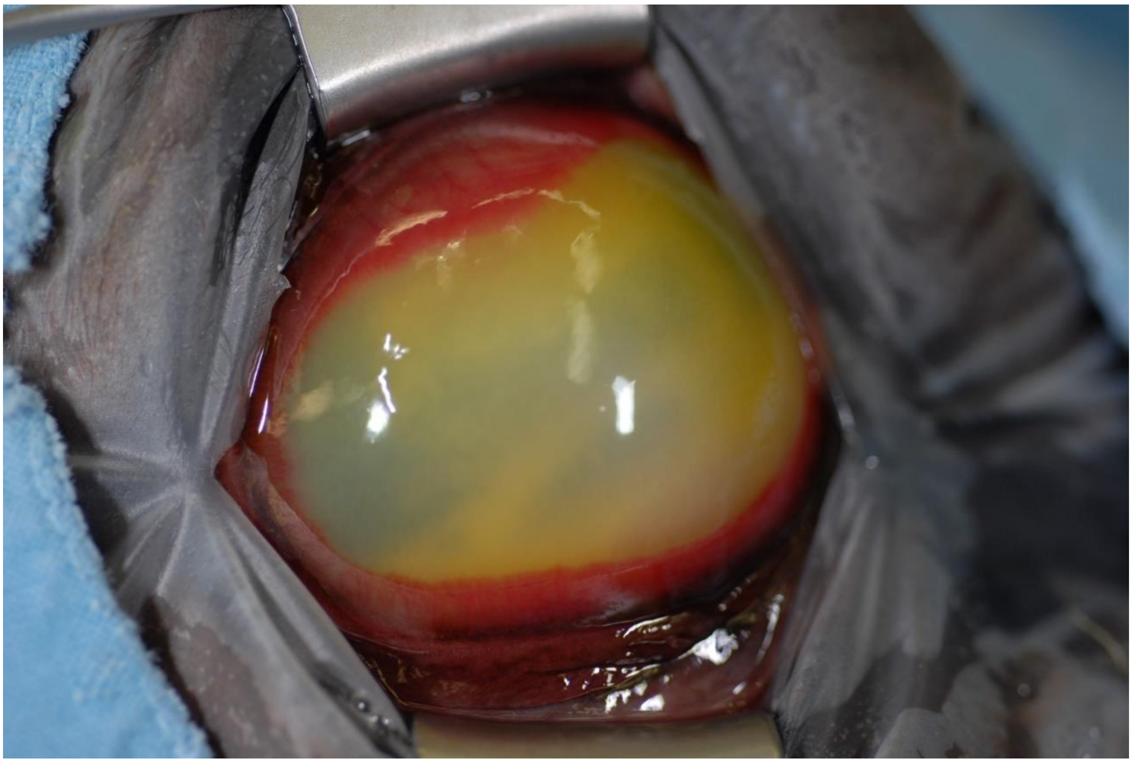

Figure S24. Septic endophthalmitis: The corneal vascularisation is much denser than vascularisation accompanying ERU. Furthermore, the purulent infection of the inner eye leads to more intense corneal edema as well as another type of cloudiness of the normally transparent media. Furthermore, the horses show fever and a significant disturbance of their general condition.

Supplementary 2

Side effects of topically administered atropine in horses.

Topically administered atropine had been considered to be a "dangerous" drug in horses, because there might occur systemic side effects (decreased intestinal motility and colic) attributable to an idiosyncratic response [556]). Shure, all horses under topical atropine treatment should be monitored closely for signs of colic, especially horses which need frequent binocular atropine treatment. Atropine from ocular administered drugs can be absorbed into the systemic circulation, causing typical parasympatholytic drug effects, such as decreased gut motility and decreased intestinal secretion. This can lead to colic with gaseous distensions and constipations ("ileus"). But this would be a toxic effect of accumulated atropine, not an allergic or immunologic reaction. But an idiosyncratic response defined as individual supersensitivity of an individual horse to atropine cannot be excluded. However, it is also known that atropine expedited the intestinal passage of two markers in normal ponies under chronic atropine administration, comparable to prolonged ocular atropine treatment [557].

The impact of side effects of topically administered atropine was put into perspective by a recent study [558], in which no side effects were apparent despite detectable plasma levels of atropine. However, when horses are hospitalized, have less exercise and stimulation, may have been moved from pasture to bay, eat more straw-bedding, may have endured long distance transports, and are stressed, colic may develop regardless of atropine administration (e.g., as a result of constipation and/or meteorism).

The authors have used 1% and 2% atropine ointments and eye drops for decades in tens of thousands of horses in a clinic. Oftentimes atropine was administered topically every hour until the pupil was dilated, sometimes bilaterally. Of course, hospitalized horses sometimes developed colic. Among these horses were eye patients who had been treated with eye drops and ointments containing atropine. However, horses with other diseases that had not received atropine were equally affected by colic. If the feces and consistency of the feces are monitored continuously, as is customary in the clinic, a reduced peristalsis and delayed fecal passage can be detected at an early stage (not only in ophthalmologic patients) and counteracted by administering laxatives. In addition, daily mash feeding can reasonably prevent obstipations. If, exceptionally, a horse develops colic while being treated with atropine, it has to be noted that, if possible, N-butylscopolamine should be avoided (e.g., exclusive administration of metamizole) and laxatives should be given at an early stage, whether or not there is a causal connection to the topical treatment with atropine.

Again: This statement is related to 1 – 2% atropine sulfate eye drops and ointments. Other concentrations as well as different application routes may lead to different observations.

Supplementary 3

Notes on the intravitreal injection of gentamicin.

Experimental studies on gentamicin injections into the vitreous body in rabbits have indicated that the half-life is approximately 3 h [559]. In the rabbit eye, only 0.08 mg could be injected into the vitreous cavity without doing any harm. After injection of 0.1 mg, initial [560] and after injection of 0.2 mg, definite [559–561] morphological changes including pigmentary alterations were seen. In addition, the ERG was still significantly altered 4 weeks after gentamicin injection [559]. After injection of 0.4 mg, the photoreceptors were destroyed and after injection of 0.8 mg, full thickness retinal necrosis was observed [562].

The vitreous body of the rabbit has a volume of about 1.5 mL, that of the human has a volume of about 4 mL [563]. If 0.08 mg can be injected intravitreally in rabbits (= about 0.05 mg/mL vitreous) without causing retinal damage, this would correspond to an injection of 0.21 mg into the human eye (the different flow dynamics in the different sized eyes are disregarded here). Therefore, knowing the experimental studies, it was recommended not to inject more than 0.2 mg (for a vitreous volume of 4 mL, this corresponds to 0.05 mg gentamicin / ml vitreous) intravitreally in humans [561]. This quantity is also not "safe" but should be accepted in view of the indication (purulent endophthalmitis).

At higher doses, macular necrosis has been repeatedly reported after gentamicin injections [564–566]. It is not possible to establish a safe or nontoxic intravitreal dose of gentamicin for human use [567] and it is discussed that gentamicin may have increased toxicity in inflamed eyes [567,568].

In order not to exceed a concentration of 0.05 mg / ml in horses, an injection of 1.4 mg gentamicin / 28 mL equine vitreous would be possible. The recommended dose of 4 mg gentamicin for intravitreal injection in horses [292,294] is nearly three times that and 6 mg [295] is more than four times the maximum recommended concentration. Some authors [294,295] refer to [293] for this high dose, whereas [292] refer to [283]. However, in [283], no intraocular gentamicin injection at all is described, only the addition of 20 mg gentamicin to the 250 mL bottle of infusion solution for irrigation of the vitreous cavity during vitrectomy. The gentamicin concentration in the infusion solution is thus only 0.08 mg / ml to avoid retinal toxicity [191]. This concentration (0.08 mg / ml) has been found to be 100-fold higher in vitro than the minimal inhibitory concentration (MIC) for pathogenic *Leptospira* spp. [372].

Supplementary 4

Table S1. Follow-up examination of aqueous samples after vitrectomy: course of anti-*Leptospira* antibody titers (MAT) in 10 horses (unpublished data).

MAT-titer at the time of vitrectomy (undiluted vitrectomy specimens)	Follow-up examination of intraocular fluids (MAT Titer and time after surgery)
1:1,600	1:400 after one week
1:3,200	1:1,600 after one week
1:51,200	1:3,200 after 3 weeks
1:1,600	1:400 after 4 months
1:3,200	1:100 after 6 months
1:800	negative after ≥ 1 year
1:6,400	negative after ≥ 1 year
1:1,200	negative after ≥ 1 year
1:3,200	negative after ≥ 1 year
1:12,800	negative after ≥ 1 year

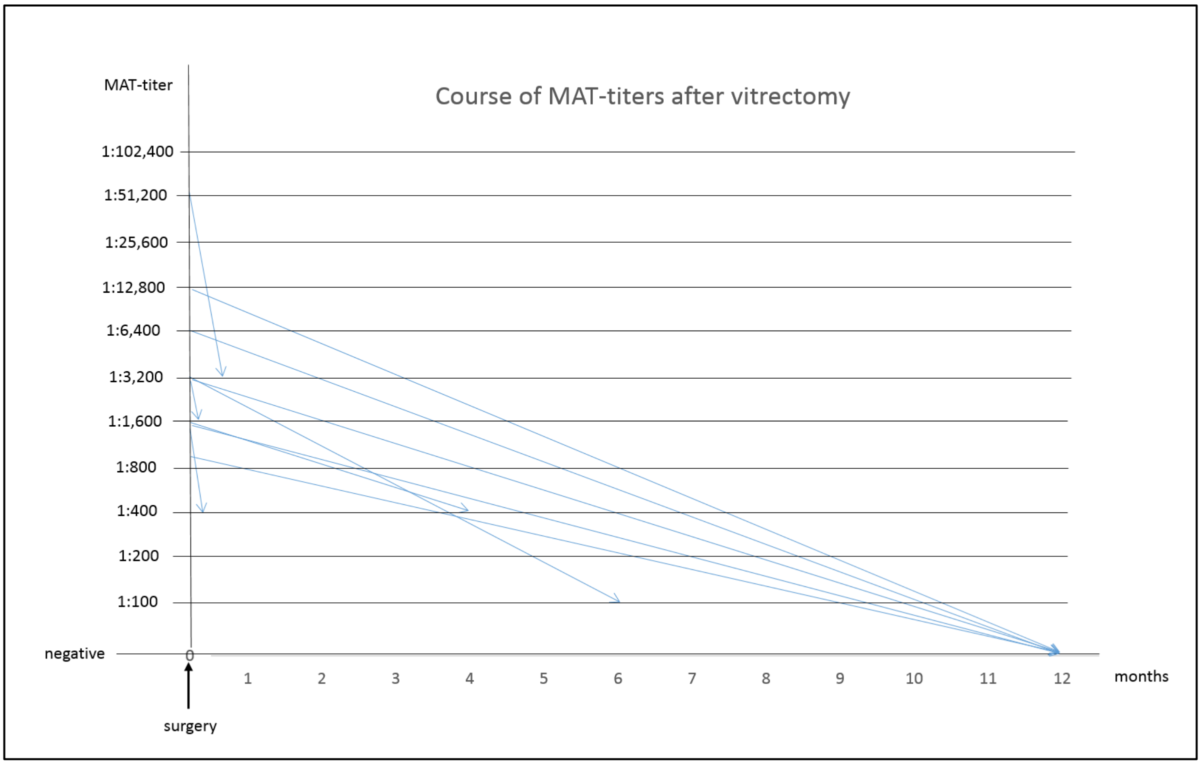

Figure S25. MAT titers over time after vitrectomy. In individual horses an aqueous humor sample could be taken at different times after surgery (e.g., when a fibrinolytic was injected after surgery or after euthanasia due to other underlying diseases). Each arrow represents one eye. The arrows start at the time of surgery and the arrowhead indicates follow-up aqueous humor testing.

Supplementary 5

Table S2. Literature references for testing intraocular samples from ERU-eyes or human uveitis-eyes for leptospires (culture, PCR, histology / immunohistochemistry and electron microscopy). (Empty fields: no information given; time: time of examination during or after systemic leptospirosis; IHC = immunohistochemistry).

Citation	Species	Uveitic Eyes [n]	Sample	Method	Time ≥	*Leptospira* spp.
Kathe 1942 [569]	human	2	aqueous	culture + microscopy	acute leptospiral infection, postmortal	
Pagani 1950 [570]	human		aqueous	animal experiment (Infection of a guinea pig with human aqueous humor, followed by darkfield microscopy of the inner organs of the animal revealing leptospires)		
Alexander et al. 1952 [571]	human	1	aqueous	culture		Alexi
Hartwigk & Stoebbe 1952 [124]	equine	1	aqueous	culture: after 3 days (!) few, weakly moving "leptospiral-like" formations; every 3 days further inoculation, after 8th passage (24 days) typical *Leptospira* spp., later contamination	„chronic" uveitis	MAT: Grippotyphosa 1:8000
			blood	culture: after 36 days single *Leptospira*-like formations, after 48 days numerous *Leptospira* spp.	acute uveitis	MAT: Grippotyphosa 1:2000
Kathe et al. 1952 [116]	(horse, aqueous) - see Hartwigk & Stoebbe 1952 [124]: identical culture, but mentioned "Canicola"					
Cimbal 1952 [572]	human		aqueous	culture + animal experiment	2 years after leptospiral infection	
Okamune & Yamamoto 1954 [573]	human		aqueous		5 years	
Fischer et al. 1955 [574]	human	2	aqueous	culture	2 years	Saxkoebing
Kemenes et al. 1960 [575]	equine	52	vitreous	culture negative<br>47 MAT pos. (90%)		
Williams 1971 [101]	equine		aqueous	culture		Pomona
Gelatt et al. 1977 [131]	equine		aqueous	3 horses culture pos.	2 weeks to 6 months after onset of ERU	3 x Pomona
Merien et al. 1993 [418]	human	1	aqueous	1 x PCR pos.	9 years after leptospirosis	
Merien et al. 1995 [576]	human	4	aqueous	3 x PCR pos.		
Chu et al. 1998 [327]	human	46	aqueous	37 x PCR pos.		
Brem et al. 1998 [373]	equine	43	vitreous (diluted)	4 x culture pos.		3 x serogroup Grippotyphosa, 1 x serogroup Australis
Brem et al. 1999 [374]	equine	130	vitreous (diluted)	35 x culture pos.		31 x serogroup Grippotyphosa<br>4 x serogroup Australis
Faber et al. 2000 [412]	equine	30 ERU-horses	aqueous	30 x PCR pos.<br>(10 horses bilateral pos.)<br>6 x culture pos.<br>(29 control eyes: 1 x PCR pos.)		cultures:<br>4 x Pomona,<br>2 x unidentified
Wollanke et al. 2000 [45]	equine	104	vitreous (undiluted)	41 x culture pos.		32 x serogroup Grippotyphosa,5 x serogroup

		92	vitreous (diluted, gentamicin: 0,08 mg /mL))	6 x culture pos.	Australis, 2 x serogroup Javanica, 1 x serogroup Pomona
		∑ 196	vitreous	47 x culture pos. (5 x MAT neg.)	
Wollanke et al. 2001 [17]	part of samples included in Wollanke et al 2004 [19]				
Wollanke et al. 2002 [18]					
Wollanke et al. 2004 [19]	equine	358	vitreous	189 x culture pos. (53%)	144 x serogroup Grippotyphosa, 25 x serogroup Australis, 9 x serogroup Sejroe, 5 x serogroup Pomona, 4 x serogroup Javanica,
		55	vitreous	39 x PCR pos. (71%)	
Niedermaier et al. 2006 [424]	equine		vitreous	ultrastructural detection of *Leptospira* spp.	
Brandes et al. 2007 [425]	equine		vitreous	ultrastructural detection of *Leptospira* spp.	
Pearce et al. 2007 [370]	equine	10	ocular tissues	2 x IHC ciliary body pos., 0 x PCR pos.	
Gilger et al. 2008 [316]	equine	28 "horses"	aqueous & vitreous sampling after euthanasia	0 x PCR pos.	
Von Borstel et al. 2010 [66]	equine	100	vitreous (diluted)	40 x PCR pos. (9 of these samples were MAT neg.)	
Popp et al. 2013 [196]	equine	33	vitreous	18 x culture pos. within 6 weeks	
		23	vitreous (Enrofloxacin > MIC)	7 x culture pos. within 6 weeks	
Polle et al. 2014 [328]	equine	46	aqueous and / or vitreous	20 x PCR pos.	
		28	aqueous and / or vitreous	6 x culture pos.	4 x serogroup Pomona, 2 x serogroup Grippotyphosa
Baake et al. 2016 [69]	equine	85	vitreous (diluted)	52 x PCR pos.	
		75	vitreous (diluted)	12 x culture pos.	10 x serogroup Grippotyphosa
Dorrego-Keiter et al. 2016 [315]	equine	212	vitreous	34 x culture pos. (16%)	25 x serogroup Grippotyphosa, 2 x serogroup Australis, 1 x serogroup Pomona
Wollanke et al 2017 [35]	equine	120 (105 eyes)	96 vitreous	70 x PCR pos. (73%)	
			24 aqueous	14 x PCR pos. (58%)	
Loibl et al. 2018 [195]	equine	79	vitreous	48 x PCR pos. (61%)	
Wollanke et al. 2018 [199]	equine	11	aqueous	2 x PCR pos. (18%)	
		107	vitreous	81 x PCR pos. (77%)	
		∑ 118	aqueous & vitreous	83 x PCR pos. (70%)	
Geiger 2019 [198]	equine	137	aqueous & vitreous	87 x PCR pos. (64%)	
Sauvage et al. 2019 [369]	equine	16	aquoeus	1 x PCR pos.	
		3	vitreous	0 x PCR pos.	
		47	aqueous & vitreous from the same eyes	8 x AH & VH PCR pos., 2 x AH PCR pos., VH PCR neg., 9 AH PCR neg. & VH PCR pos., 28 AH & VH PCR neg.	

Himebaugh & Gilger 2021 [14]	equine	39	aqueous	16 x PCR pos.
Ackermann et al. 2021 [57]	equine	32 (PCR pos.)	vitreous	IHC, biofilm formation

Supplementary 6

Results of examinations of intraocular samples from horses suffering from ERU. (Excerpts from [18] and Figure S26)

Table S3. Protein fractions determined by electrophoresis (total protein "TP" and albumin "Alb.") and calculated (globulins = "Ig") from serum (S) and vitreous (V) samples and calculation of the Goldmann-Witmer coefficient (GWC) in 46 paired vitreous and serum samples. Results sorted by GWC (decreasing).

serum (g/l)			vitreous (g/l)			Ig (S)/ Ig (V)	1/titer serum	1/titer vitreous	GWC
Ig	Alb.	TP	Ig	Alb.	TP				
30,9	27,5	58,4	2,2	4,9	7,1	14	400	51200	1792
44,9	25,6	70,5	6,9	3,9	10,8	6,5	200	51200	1664
42	30,4	72,4	7,7	3,9	11,8	5,5	100	12800	704
40,1	30,8	70,9	8,9	6,8	15,7	4,5	800	102400	576
44,9	25,6	70,5	6,1	2,7	8,8	7,4	200	12800	473,6
35,9	30,1	66	8,5	7,2	15,7	4,2	400	25600	268,8
36,7	26,3	63	5,3	1,5	6,8	6,9	200	6400	220,8
39,8	31,8	71,6	7,4	5,2	12,6	5,4	100	3200	172,8
29,8	29,7	59,5	0,2	0,1	0,3	149	200	200	149
32,2	31,7	63,9	7,6	6,2	13,8	4,2	400	12800	134,4
37,6	30	67,6	1,2	1,2	2,4	31,3	100	400	125,2
35,9	26,1	62	1,2	0,4	1,6	29,9	100	400	119,6
41,5	29,4	70,9	1,4	0,8	2,2	29,6	800	3200	118,4
41,7	29	70,7	2	1,2	3,2	20,9	400	1600	83,6
34,9	24,8	59,7	7,8	2,2	10	4,5	200	3200	72
41,5	29,4	70,9	2,6	1,8	4,4	16	800	3200	64
39,1	26,6	65,7	2,8	1,1	3,9	14	400	1600	56
38,7	26,2	64,9	5,8	2,9	8,7	6,7	200	1600	53,6
34,6	31,1	65,7	5,8	3,2	9	6	400	3200	48
31,8	28,2	60	1,6	0,5	2,1	19,9	400	800	39,8
36,3	32,8	69,1	1,2	0	1,2	35,1	100	100	35,1
41,7	29	70,7	0,3	0	0,3	139	400	100	34,8
31,7	31,3	63	2	0,6	2,6	15,9	1600	3200	31,7
32,4	27,6	60	0,7	0,1	0,8	46,3	200	100	23,2
39,5	28,6	68,1	7,1	5,3	12,4	5,6	200	800	22,4
30,7	33,1	63,8	3,3	1,8	5,1	9,3	400	800	18,6
35,1	31,2	66,3	4	0,9	4,9	8,8	200	400	17,6
32,6	27,6	60,2	2,1	0,2	2,3	15,5	100	100	15,5
28,3	28,9	57,2	5,8	5,7	11,5	4,9	400	12800	14,7
38,2	29,8	68	5,3	0	5,3	7,2	400	800	14,4
30,4	28,1	58,5	1,2	0,5	1,7	25,3	200	100	12,65
26,7	33,5	60,2	2,6	1,9	4,5	10,3	100	100	10,3
31,7	31,3	63	6,4	3,7	10,1	5	1600	3200	10
39,2	29,5	68,7	1,2	2,6	3,8	32,7	400	100	8,2
28,3	29,7	58	9,7	12,4	22,1	2,9	100	200	5,8
31,6	28,3	59,9	8,9	7,8	16,7	3,6	1600	800	1,8

33,8	27,7	61,5	1	0,1	1,1	33,8	200	-	-
32,5	29,7	62,2	1,3	0,1	1,4	31,2	100	-	-
30,1	27,4	57,5	1,5	0,3	1,8	20,1	-	-	-
35,1	33,2	68,3	1,8	0,3	2,1	19,5	400	-	-
36,7	26,3	63	2,4	0,7	3,1	15,3	200	-	-
38,5	33,4	71,9	2,7	0,9	3,6	14,3	100	-	-
29,3	26,8	56,1	2,6	1,1	3,7	15,2	-	1600	+
25,5	31,1	56,6	4,8	0,6	5,4	5,3	400	-	-
33,3	29,2	62,5	3,4	2,7	6,1	9,8	-	400	+
36,6	31,1	67,7	5,5	0,9	6,4	6,7	-	1600	+

Table S4. MAT-titers in serum (S) and undiluted vitreous samples (V) from horses with ERU and from horses with healthy eyes. Intraocular MAT titers exceed serum titers several times. A clear difference between horses suffering from ERU and horses with healthy eyes is only seen when looking at the intraocular samples.

MAT (reciprocal titer)	S ERU (724 samples)		S healthy eyes (132 samples)		V ERU (426 samples)		V healthy eyes (54 samples)	
	n	[%]	n	[%]	n	[%]	n	[%]
negative	96	13.3	26	19.7	44	10.3	51	94.4
100	149	20.6	28	21.2	24	5.6	3	5.6
200	176	24.3	34	25.8	29	6.8	0	0
400	154	21.3	21	15.9	37	8.7	0	0
800	77	10.6	15	11.4	38	8.9	0	0
1,600	55	7.6	5	3.8	59	13.8	0	0
3,200	12	1.7	1	0.8	70	16.4	0	0
6,400	3	0.4	0	0	27	6.3	0	0
12,800	1	0.1	2	1.5	36	8.5	0	0
25,600	1	0.1	0	0	27	6.3	0	0
51,200	0	0	0	0	20	4.7	0	0
102,400	0	0	0	0	8	1.9	0	0
204,800	0	0	0	0	3	0.7	0	0
409,600	0	0	0	0	1	0.2	0	0
819,200	0	0	0	0	2	0.5	0	0
1.638,400	0	0	0	0	0	0	0	0
3.267,800	0	0	0	0	1	0.2	0	0

Table S5. MAT-results with intraocular and serum samples from horses with ERU (ophthalmological findings differentiated) and from horses with healthy eyes. (pos. = MAT positive 1:100 or higher).

	Serum			Vitreous			Aqueous		
**Ophthalmological Findings**	**n**	**pos. [%]**	**$\bar{x}$ [1:titer]**	**n**	**pos. [%]**	**$\bar{x}$ [1:titer]**	**n**	**pos. [%]**	**$\bar{x}$ [1:titer]**
healthy eyes	132	80	203	54	5	52	26	12	62
ERU	724	87	245	426	90	1680	34	94	2511
ERU – anterior uveitis	73		201	46		566			
ERU – intermediate uveitis	301		246	224		1727			
ERU – panuveitis	239		245	157		2111			
ERU – mild	97		248	73		489			
ERU – moderate	240		214	194		1556			
ERU – severe	283		258	160		3070			
ERU < 1 year	309		241	259		1622			
ERU > 1 year	270		250	183		1213			

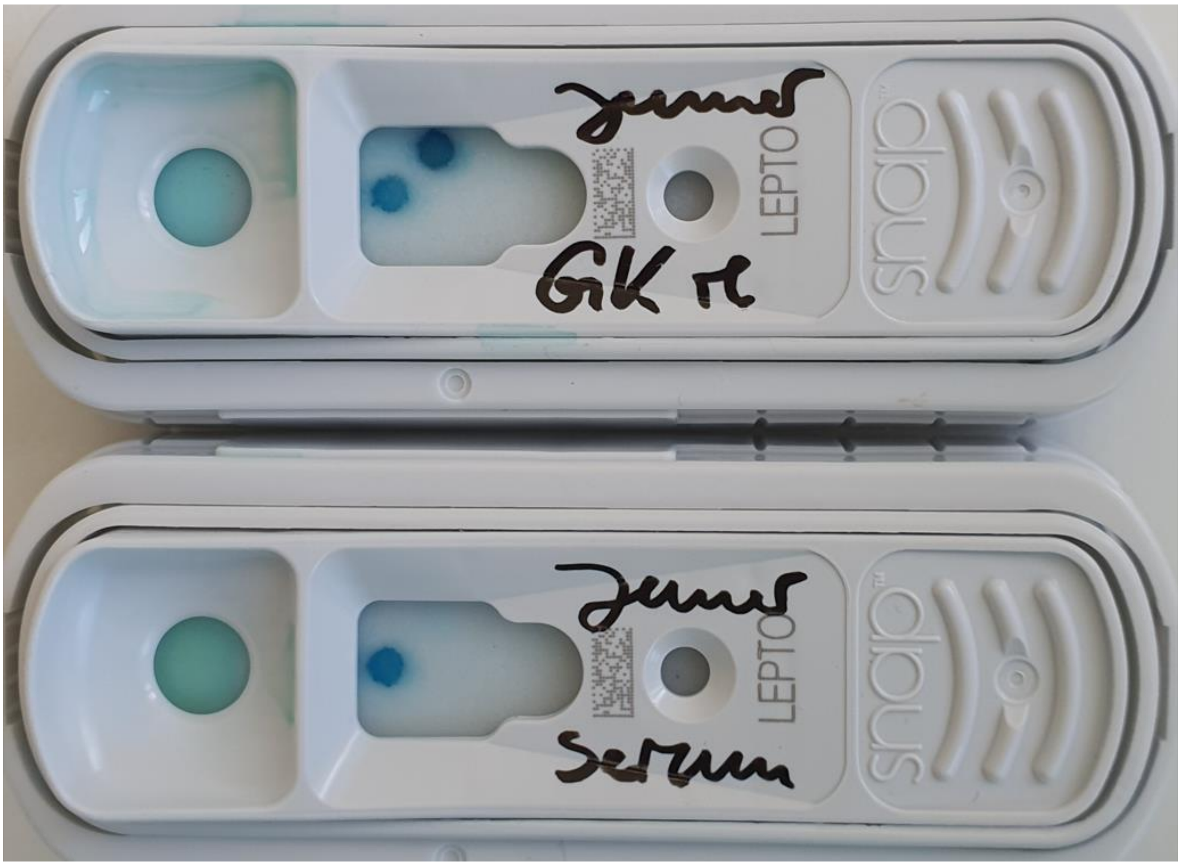

Figure S26. Typical SNAP Lepto-results in an ERU horse. Upper picture: SNAP Lepto with undiluted vitreous from an ERU-eye. Picture below: SNAP Lepto with serum of the same horse. The sample point is at the "12 o'clock-position" and the control point is at the "9 o'clock-position", respectively. The point of the vitreous sample is even much darker than the control point, indicating a high level of intraocular antibodies. The point of the serum sample shows only a very weak coloring which is just barely visible. (SNAP Lepto = commercially available quick ELISA test for detection of anti-*LipL32* antibodies from IDEXX company, Ludwigsburg, Germany). (Photograph: BW).

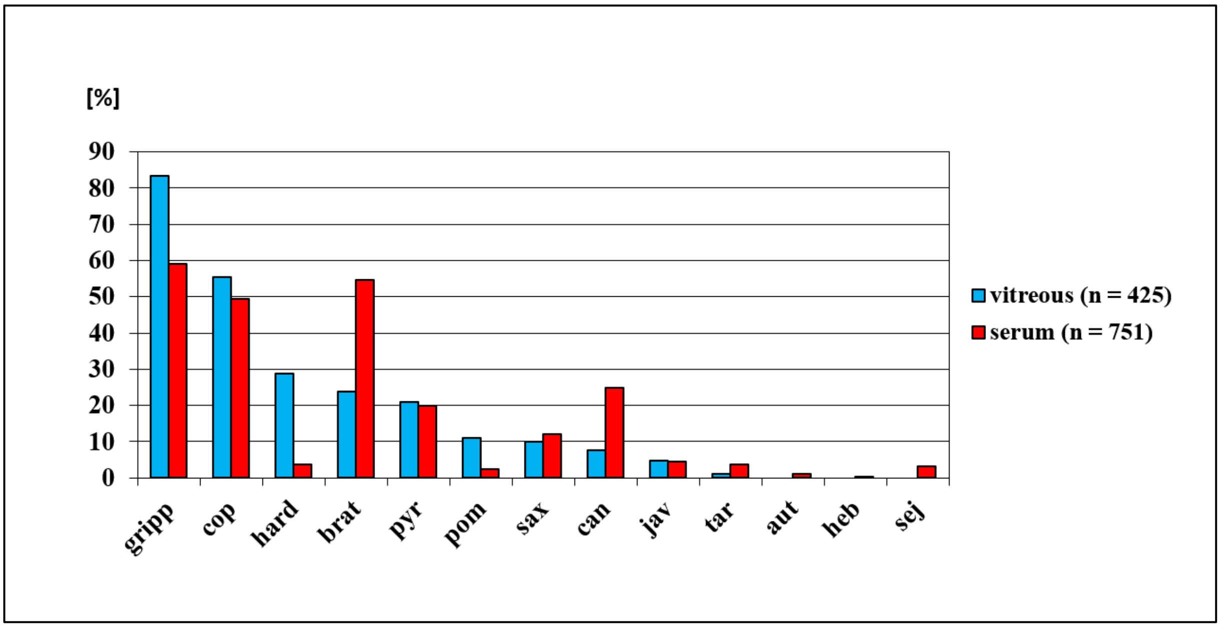

Figure S27. Percentage of positive reacting vitreous and serum samples from ERU-eyes and ERU-horses, respectively, using MAT (titer ≥ 1:100).

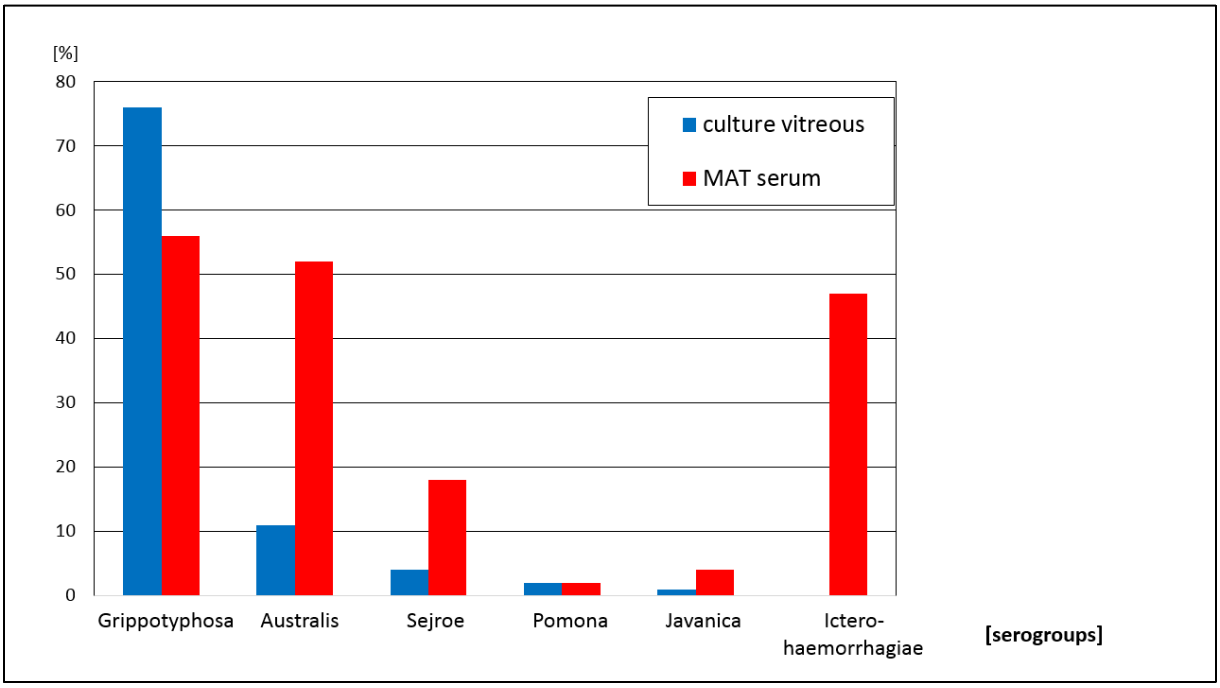

Figure S28. Positive leptospiral culture results (n = 189) with vitreous samples compared to MAT results in serum samples. Percentage of assignment to serogroups. Sometimes MAT was positive (titer ≥ 1:100) for more than one serovar.

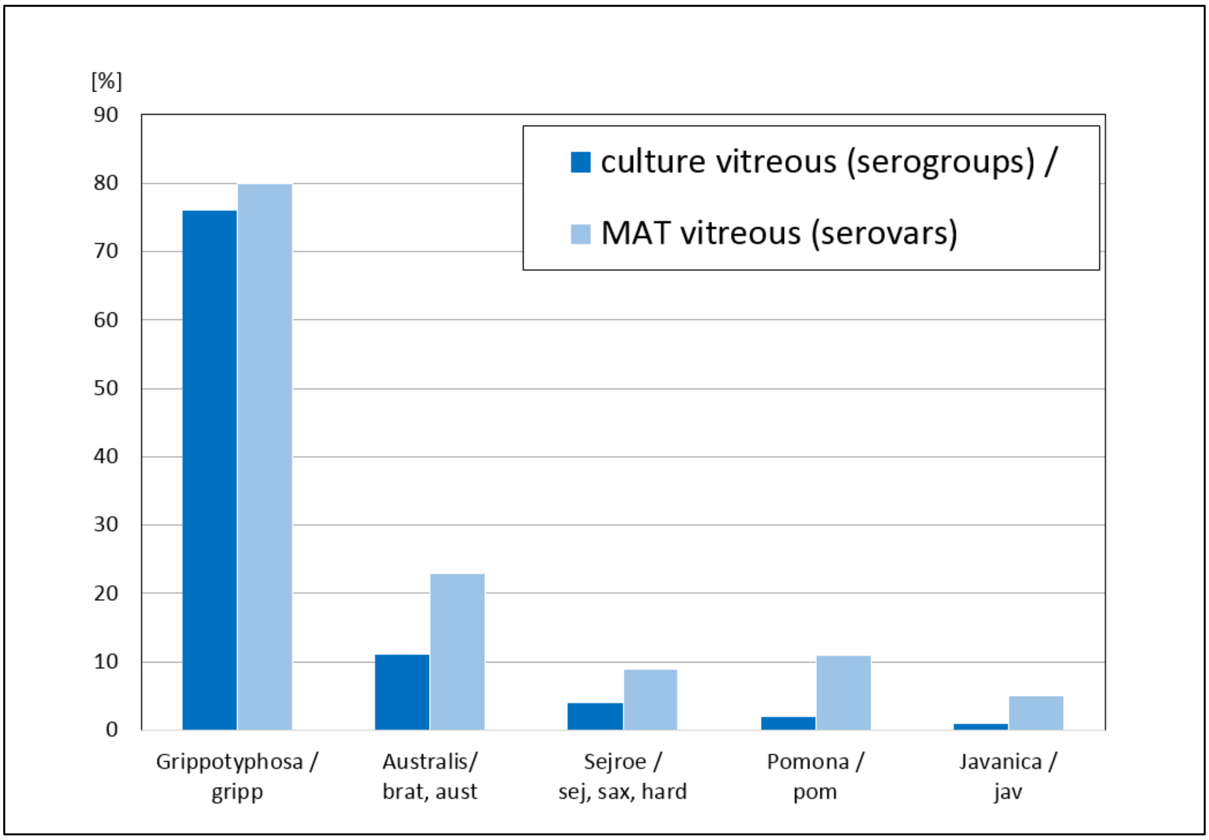

Figure S29. Positive leptospiral culture results (n = 189) with vitreous samples compared to MAT results in the same samples. Percentage of assignment to serogroups. Sometimes MAT was positive (titer ≥ 1:100) for more than one serovar.

Supplementary 7

Literature references cited in publications on the examination of intraocular specimens from eyes affected with ERU for the calculation of GWC or "C-values".

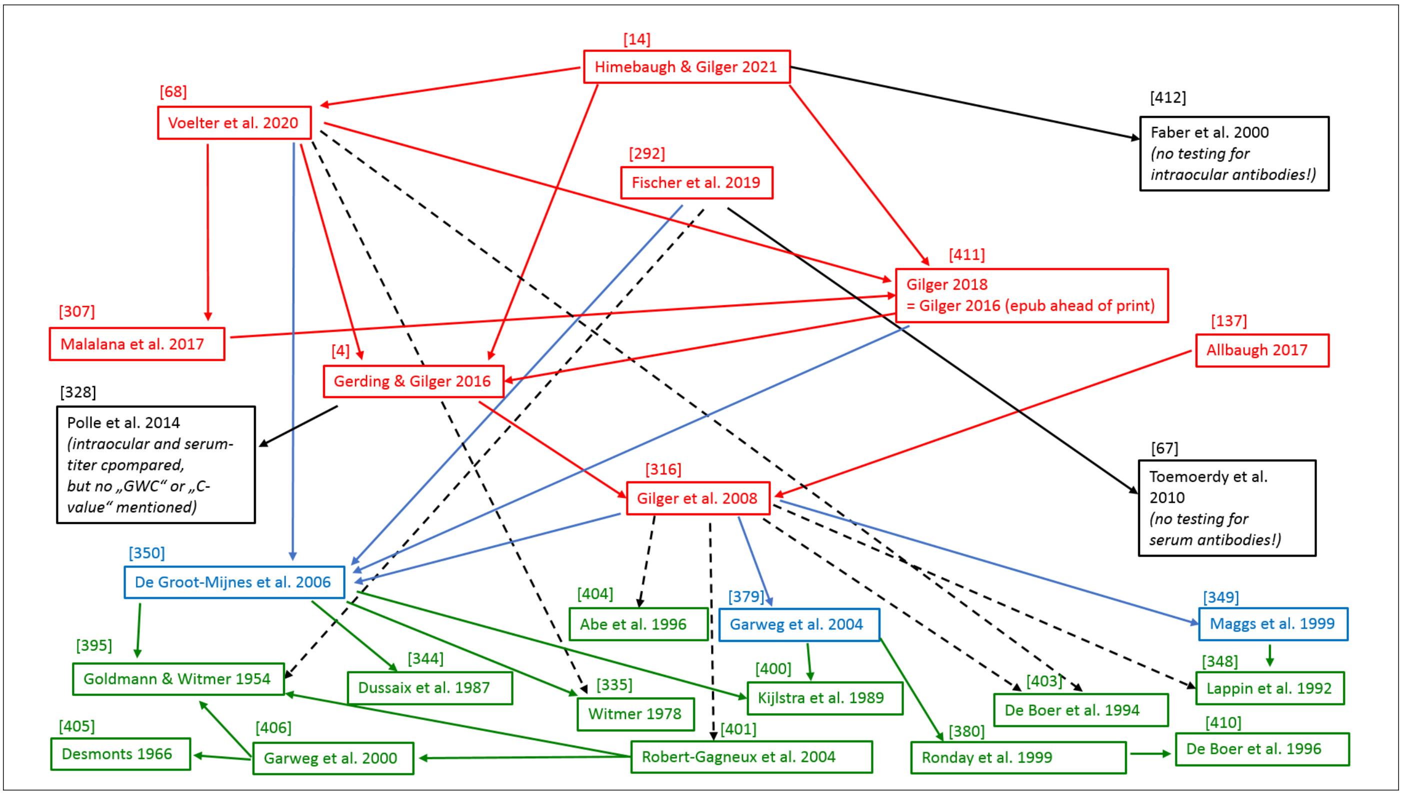

Figure S30. References for calculation and interpretation of the Goldmann-Witmer-Coefficient (GWC) in the literature. Arrows mean "cites". **Red references**: Calculation of "C-value" without any additional testing e.g., for the IgG-, albumin or total protein content in corresponding intraocular and serum samples. Black references**:** No calculation of the GWC at all - although cited for the GWC. **Blue references**: Exact calculation path for correct GWC-calculations is not described, but measurement of the IgG-levels in intraocular fluids and serum samples is included in the methods and correct citations for the calculation of the GWC are given. **Green references**: Correct calculation paths of GWC are given. Black arrows: Citation of references which did not use or describe the GWC for their work at all. **Red arrows**: Citation of references for the calculation of the GWC or "C-value", respectively, in which the calculation is not described. Dashed arrows: Citation of references in which the correct calculation of the GWC is described, but the citing reference ignores the measurement of IgG or another protein fraction anyway. **Blue arrows**: Citation of references in which the methods describe measurement of IgG-levels and cite references for correct calculation paths of the GWC. **Green arrows**: Correct citations for calculation of the GWC.

Supplementary 8

Ultrastructural and histological examinations of vitreous specimens and the ciliary body.

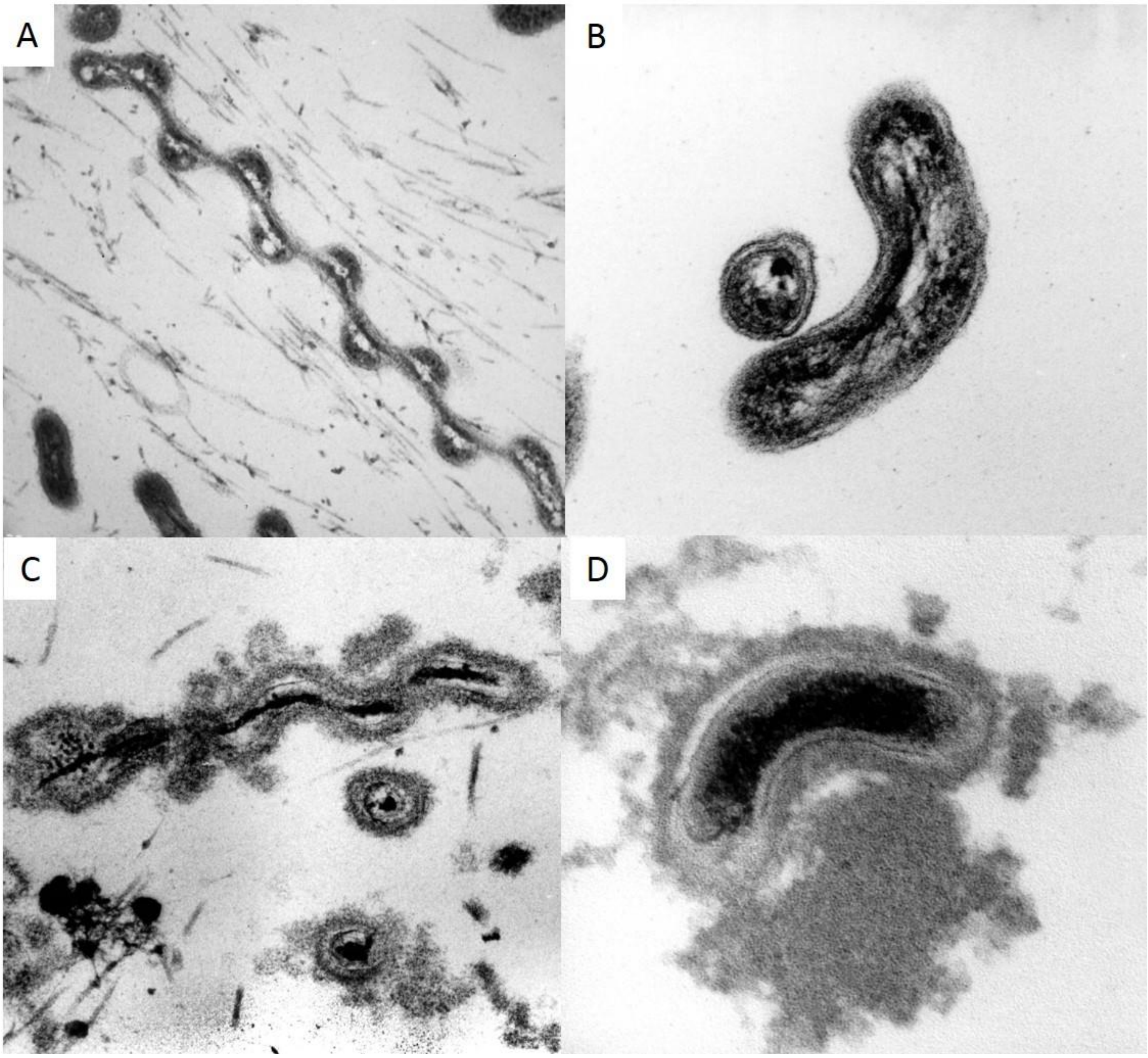

Figure S31. Transmission electron microscopy pictures. A and B: *Leptospira grippotyphosa* (WHO standard strain) were experimentally injected into the vitreous body of a healthy eye of a euthanized horse. Afterwards, the vitreous sample was taken by vitrectomy. A: *Leptospira* spp. seem to arrange themselves along with the vitreous fibers which would be an excellent staring position for biofilm formation. C and D: Vitreous samples taken during therapeutic vitrectomy from a horse with naturally acquired ERU. *Leptospira* spp. are surrounded by a thick layer of an extracellular osmiophilic matrix which lacks the experimentally injected *Leptospira* spp. (Source: [424], reprint courtesy of Schlueterschen Specialized Media GmbH, Hanover, Germany).

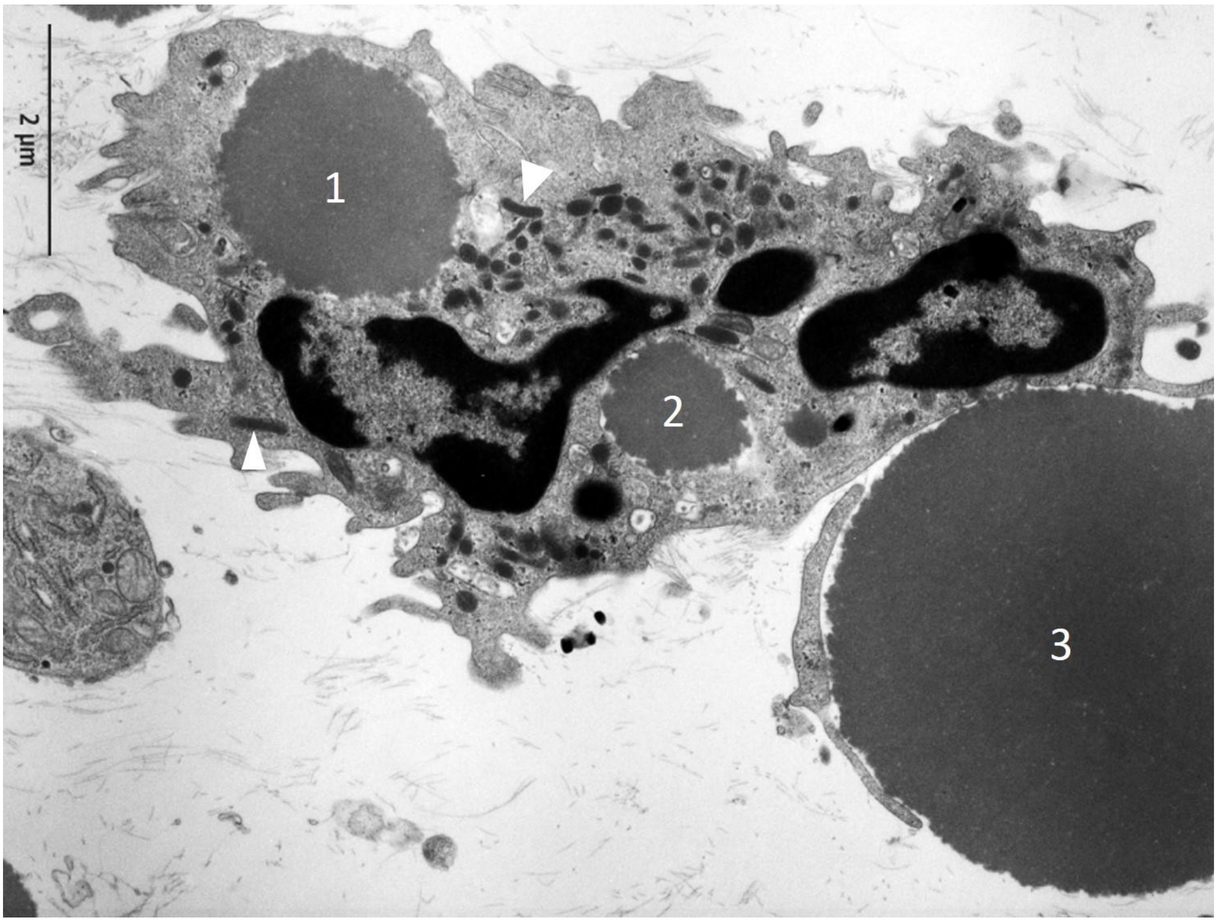

Figure S32. Vitreous material from an ERU-eye prepared for transmission electron microscopy showing phagocytosis of dense round structures. Two smaller ones are inside the phagocyte (numbers 1 and 2), the large one (number 3) probably leads to a "frustrated phagocytosis". These dense round structures are now suspected to be leptospiral biofilm. Inside of the phagocyte are also structures which may be parts of *Leptospira* spp. (arrowheads). Bar = 2 µm (Photograph courtesy of Kristin Brandes).

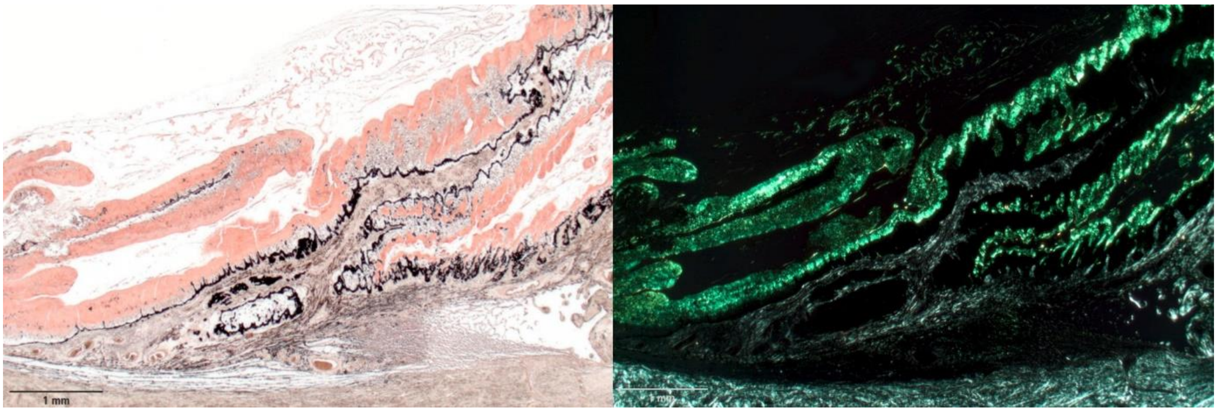

Figure S33. Ciliary body region of an ERU-eye. Congo red staining and polarized light showing amyloid on the ciliary body. Subsequent immunohistology revealed amyloid A [224]. (Photographs courtesy of Maj-Britt Cielewicz).

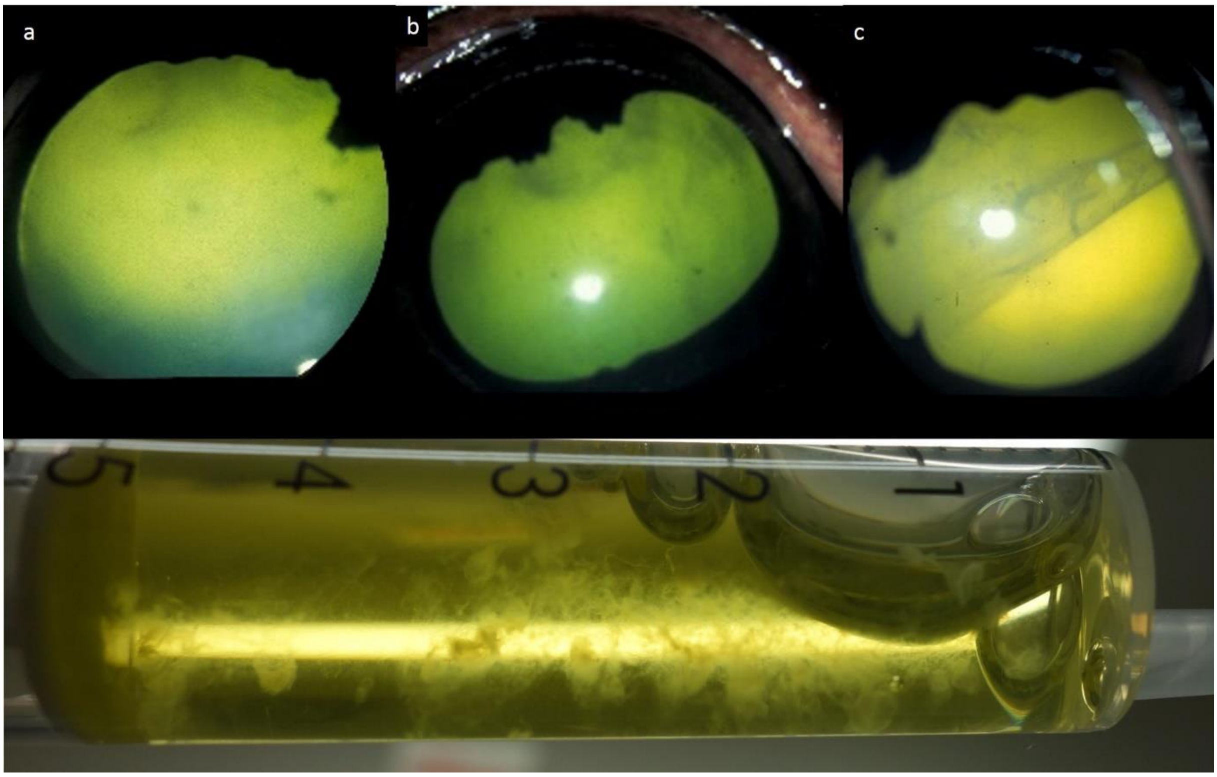

Figure S34. Dens vitreous opacities ("floaters"). a: In the dorsal aspect of the vitreous chamber, b: Further distributed in the vitreous chamber, c: Membrane-like vitreous opacity mimicking retinal detachment. Bottom: Syringe containing vitreous material (and several "floaters") obtained in the beginning of vitrectomy. (Photographs: BW and HG).

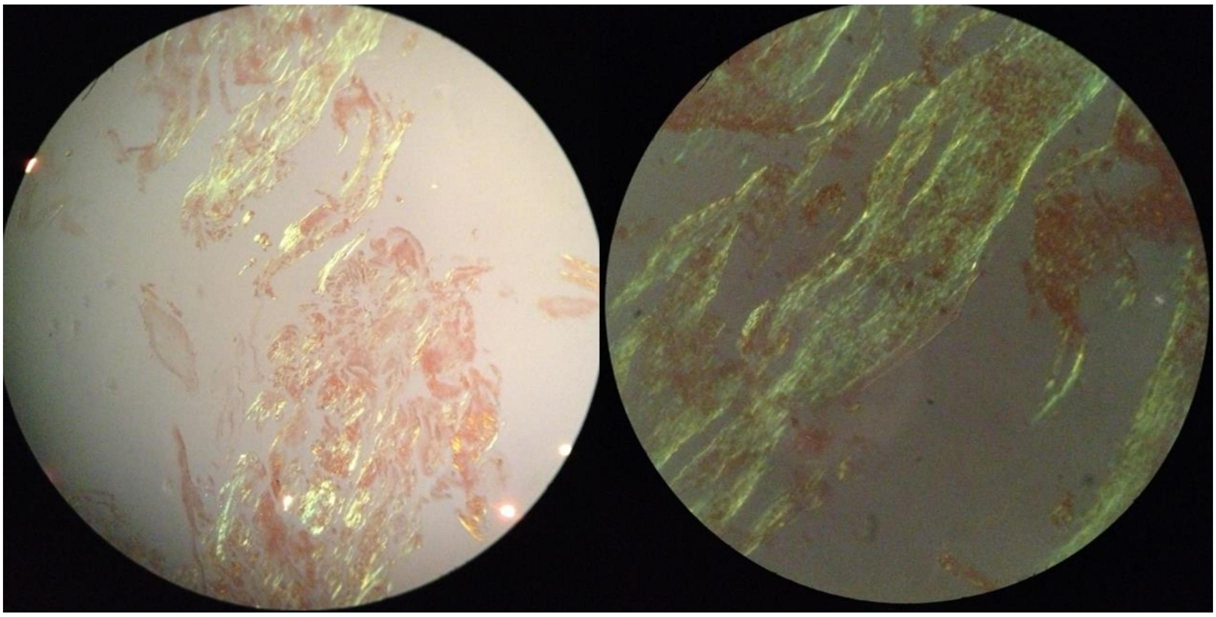

Figure S35. Amyloid: Vitreous opacities (Supplementary 1, Figure S13 and Supplementary 9, Figure S34) after Congo Red staining and the presence of green birefringence in polarized light. (Photographs courtesy of Ellen Giving).

Supplementary Materials: The following are available online at www.mdpi.com/xxx/s1, **Supplementary 1, Figure S1:** Acute uveitis: Corneal haziness in the periphery, beginning corneal vascularization (visible in the dorsal and temporal aspects), miotic pupil. The accompanying conjunctivitis (hyperemia) seems to be less severe after anesthetic eye drops which cause vasoconstriction. **Figure S2:** Acute uveitis and similar findings like in figure 1, but the circular corneal vascularization is more severe. **Figure S3:** Left: Small amount of fibrin in the anterior chamber, early stage and mild ERU-bout. Right: Severe ERU, hypopyon and corneal vascularization. **Figure S4:** Severe ERU, the

same eye is in both pictures. Left: Corneal haziness in the periphery, corneal vascularization, sero-hemorrhagic inflammation, miotic pupil. Right: After one week with rigorous conservative therapy. The corneal opacity and the inflammatory products in the anterior chamber are decreasing, the pupil is dilated about 2/3 and the fundus reflex indicates substantial vitreous haziness. **Figure S5:** Left: Very small amount of fibrin (arrow) in the anterior chamber which might be missed if the anterior chamber is not examined carefully. It is not possible to know if this was a mild ERU-bout or a blunt trauma. Right: Acute ERU-bout after a few days of conservative Therapy. The amount of fibrin (arrow) decreases, the pupil is more dilated after frequent administration of atropine ointments and the fundus reflex indicates a diffuse vitreous haziness. **Figure S6:** Increasing vitreous haziness in ERU. a: Normal fundus reflex, b – f: Increasing vitreous haziness, f: The orange-red color indicates a high risk for retinal detachment. **Figure S7:** Normal view of the optic nerve disc with centrifugal vessels of the equine paurangiotic fundus, b – f: Increasing vitreous haziness, it becomes more and more difficult to see details or even the outline the optic nerve disc. **Figure S8:** Chronic ERU. Left: Subacute uveitis, rubeosis iridis and neovascularization, posterior synechia, cataract formation. Right: Quiet interval, posterior synechia and cataract formation. **Figure S9:** Left: Atrophy of the globe, posterior synechiae, cataract, and a "third corner" of the eyelids (arrow) as a sequela of the atrophy. Right: Phthtisis after ERU (= "end-stage") with chronic ocular discharge. **Figure S10:** Chronic ERU. Left: Subacute uveitis, posterior synechia, severe vitreous haziness. Right: Quiet interval, extensive posterior synechia, beginning cataract formation. **Figure S11:** Chronic ERU. Left: Posterior synechia and cataract formation. Right: posterior and anterior synechia and cataract formation. Anterior synechia lead to the corneal opacities. **Figure S12:** Chronic ERU and vesicular cataracts subcapsular of the posterior lens capsule. Left: Vesicular cataract in the nasal aspect. Temporally small posterior synechia. Middle: Vesicular cataract (very small "bubbles") especially in the ventral aspect of the lens. At the "7 o'clock"-position and at the "11 o'clock"-position small posterior synechia. In the dorsal aspect inflammatory products on the posterior lens capsule. Right: Vesicular cataract in the periphery (relatively large or possibly confluent "bubbles"). At the "1 o'clock"-position small vitreous floaters. **Figure S13:** Chronic ERU, quiet interval. Arrowhead: Iris residuae on the anterior lens capsule. Arrow: Dense inflammatory products ("floaters") in the vitreous cavity, very close to the posterior lens capsule. These inflammatory products move ("float") in the vitreous after blinking and can best be seen and assessed using a hand-held ophthalmoscope. **Figure S14:** Chronic ERU. Star-shaped retinal folds (arrows) around the optic nerve disc. This degree of retinal detachment means an increased risk for retinal detachment. If vitrectomy is successfully performed and there is no retinal detachment perioperatively, vision might be preserved. **Figure S15:** Chronic ERU. Left: Large-scale detachment of the retina. This kind of detachment will progress, leading to blindness. Right: Complete retinal detachment. The retina is still fixed around the optic nerve disc, but no longer at the dorsal and lateral aspects of the Ora serrata. **Figure S16:** Leopard coat pattern uveitis: Cataract and posterior lens luxation in the left eye. **Figure S17:** Uveitis: Blood and fibrin in the anterior chamber. These findings may be due to a blunt trauma. **Figure S18:** Phacogenic uveitis: protrusion of lens material (arrow) through a circular defect in the anterior lens capsule. In the ventral aspect of the lesion iris pigment is left after posterior synechia. **Figure S19**: Chronic iritis, similar to "Fuchs' heterochromic iritis" in humans: Depigmentation in the iris and corneal oedema. **Figure S20:** Uveitis (both eyes affected) accompanying septicaemia (*Rhodococcus equi*). In foals younger than 6 months ERU is extremely unlikely. **Figure S21:** Medulloepithelioma causing mild and insidious uveitis. **Figure S22:** Ongoing painful uveitis despite rigorous conservative therapy in a horse with systemic *Micronema deletrix* (syn: *Halicephalobus deletrix*) infection. The nematodes were later histologically detected in the uveal tissue. **Figure S23:** Uveitis accompanying severe keratitis. The focus must be on the corneal infection. Once the infection is removed, the uveitis is not going to continue. **Figure S24:** Septic endophthalmitis: The corneal vascularisation is much denser than vascularisation accompanying ERU. Furthermore, the purulent infection of the inner eye leads to more intense corneal edema as well as another type of cloudiness of the normally transparent media. Furthermore, the horses show fever and a significant disturbance of their general condition. **Supplementary 2**, Notes on side effects of topically administered atropine in horses. **Supplementary 3**, Notes on the intravitreal injection of gentamicin. **Supplementary 4, Table S1:** Follow-up examination of aqueous samples after vitrectomy: course of anti-*Leptospira* antibodytiters (MAT) (unpublished data). **Figure S25**: MAT titers over time after vitrectomy. In individual horses an aqueous humor sample could be taken at different times after surgery (e.g., when a fibrinolytic was injected after surgery or after euthanasia due to other underlying diseases). Each arrow represents one eye. The arrows start at the time of surgery and the arrowhead indicates follow-up aqueous humor testing. **Supplementary 5, Table S2:** Literature references for testing intraocular samples from ERU-eyes or human uveitis-eyes for leptospires (culture, PCR, histology / immunohistochemistry and electron microscopy).

Supplementary 6, Results of examinations of intraocular samples from horses suffering from ERU (Excerpts from [18]). **Table S3:** Protein fractions determined by electrophoresis (total protein "TP" and albumin "Alb.") and calculated (globulins = "Ig") from serum (S) and vitreous (V) samples and calculation of the Goldmann-Witmer coefficient (GWC) in 46 paired vitreous and serum samples. Results sorted by GWC (decreasing). **Table S4:** MAT-titers in serum (S) and undiluted vitreous samples (V) from horses with ERU and from horses with healthy eyes. Intraocular MAT titers exceed serum titers several times. A clear difference between horses suffering from ERU and horses with healthy eyes is only seen when looking at the intraocular samples. **Table S5:** MAT-results with intraocular and serum samples from horses with ERU (ophthalmological findings differentiated) and from horses with healthy eyes. **Figure S26:** Typical SNAP Lepto-results in an ERU horse. Upper picture: SNAP Lepto with undiluted vitreous from an ERU-eye. Picture below: SNAP Lepto with serum of the same horse. The sample point is at the "12 o'clock-position" and the control point is at the "9 o'clock-position", respectively. The point of the vitreous sample is even much darker than the control point, indicating a high level of intraocular antibodies. The point of the serum sample shows only a very weak coloring which is just barely visible. (SNAP Lepto = commercially available quick ELISA test for detection of anti-*LipL32* antibodies from IDEXX company, Ludwigsburg, Germany) (Photograph: BW). **Figure S27:** Percentage of positive reacting vitreous and serum samples from ERU-eyes and ERU-horses, respectively, using MAT (titer ≥ 1:100). **Figure S28:** Positive leptospiral culture results (n = 189) with vitreous samples compared to MAT results in serum samples. Percentage of assignment to serogroups. Sometimes MAT was positive (titer ≥ 1:100) for more than one serovar. **Figure S29:** Positive leptospiral culture results (n = 189) with vitreous samples compared to MAT results in the same samples. Percentage of assignment to serogroups. Sometimes MAT was positive (titer ≥ 1:100) for more than one serovar. **Supplementary 7**, Literature references cited in publications on the examination of intraocular specimens from eyes affected with ERU for the calculation of GWC or "C-values". **Figure S30:** References for calculation and interpretation of the Goldmann-Witmer-Coefficient (GWC) in the literature. Arrows mean "cites". Red references: Calculation of "C-value" without any additional testing e.g., for the IgG-, albumin or total protein content in corresponding intraocular and serum samples. Black references: No calculation of the GWC at all - although cited for the GWC. Blue references: Exact calculation path for correct GWC-calculations is not described, but measurement of the IgG-levels in intraocular fluids and serum samples is included in the methods and correct citations for the calculation path are given. Green references: Correct calculation paths of GWC are given. Black arrows: Citation of references which did not use or describe the GWC at for their work at all. Red arrows: Citation of references for the calculation of the GWC or "C-value", respectively, in which the calculation is not described. Dashed arrows: Citation of references in which the correct calculation of the GWC is described, but the citing reference ignores the measurement of IgG or another protein fraction anyway. Blue arrows: Citation of references in which the methods describe measurement of IgG-levels and cite references for correct calculation paths of the GWC. Green arrows: Correct citations for calculation of the GWC. **Supplementary 8**, Ultrastructural and histological examinations of vitreous specimens and the ciliary body. **Figure S31:** Transmission electron microscopy pictures. A and B: *Leptospira grippotyphosa* (WHO standard strain) were experimentally injected into the vitreous body of a healthy eye of a euthanized horse. Afterwards, the vitreous sample was taken by vitrectomy. A: *Leptospira* spp. seem to arrange themselves along with the vitreous fibers which would be an excellent staring position for biofilm formation. C and D: Vitreous samples taken during therapeutic vitrectomy from a horse with naturally acquired ERU. *Leptospira* spp. are surrounded by a thick layer of an extracellular osmiophilic matrix which lacks the experimentally injected *Leptospira* spp. (Source: [424], reprint courtesy of Schluetersche Specialized Media GmbH, Hanover, Germany). **Figure S32:** Vitreous material from an ERU-eye prepared for transmission electron microscopy showing phagocytosis of dense round structures. Two smaller ones are inside the phagocyte (numbers 1 and 2), the large one (number 3) probably leads to a "frustrated phagocytosis". These dense round structures are now suspected to be leptospiral biofilm. Inside of the phagocyte are also structures which may be parts of *Leptospira* spp. (arrowheads). Bar = 2 µm (Photograph courtesy of Kristin Brandes). **Figure S33:** Ciliary body region of an ERU-eye. Congo red staining and polarized light showing amyloid on the ciliary body. Subsequent immunohistology revealed amyloid A [224]. (Photographs courtesy of Maj-Britt Cielewicz). **Figure S34:** Dens vitreous opacities ("floaters"). a: In the dorsal aspect of the vitreous chamber, b: Further distributed in the vitreous chamber, c: Membrane-like vitreous opacity mimicking retinal detachment. Bottom: Syringe containing vitreous material (and several "floaters") obtained in the beginning of vitrectomy. (Photographs: BW and HG). **Figure S35:** Amyloid: Vitreous opacities (Supplementary 1, Figure S13 and Supplementary 9, Figure S34) after Congo Red staining and the presence of green birefringence in polarized light. (Photographs courtesy of Ellen Giving).
